# Natural products shape microbial ecology and disease in the cystic fibrosis airway

**DOI:** 10.1039/d6np00067c

**Published:** 2026-10-21

**Authors:** Niladri Bhowmik, Reed M. Stubbendieck

**Affiliations:** a Department of Microbiology and Molecular Genetics, Oklahoma State University Stillwater OK 74078 USA stubbendieck@okstate.edu

## Abstract

Covering up to 2026

Cystic fibrosis (CF) is a life-shortening autosomal recessive genetic disorder caused by mutation in the gene encoding the CF transmembrane conductance regulator (CFTR). Dysfunction in CFTR leads to the accumulation of thick and dehydrated mucus in the respiratory tract, which serves as a substrate for a complex microbiota that includes commensal oral and respiratory bacteria and opportunistic pathogenic bacteria and fungi. During chronic infections, these microbes persist in the airways and contribute to progressive lung tissue damage, ultimately leading to respiratory failure and premature death. Within this environment, microbes synthesize a diverse repertoire of natural products, including antimicrobials, siderophores and other metallophores, signaling molecules, toxins, and other virulence factors. In this review, we thoroughly discuss natural products produced by members of the CF airway microbiota, primarily the bacterial pathogens *Burkholderia* spp., *Pseudomonas aeruginosa*, and *Staphylococcus aureus* and the fungal pathogens *Aspergillus fumigatus* and *Scedosporium* spp. When known, we describe the biosynthesis and roles of these natural products in microbial persistence, interspecies interactions, and virulence in the CF airway. When these roles are unknown, we infer potential roles based on evidence from the literature in different disease contexts. Finally, we briefly highlight natural products produced by the CF microbiota as novel sources for developing new antimicrobials and as drug targets to treat disease.

## Abstract 2

This review highlights how microbial natural product antibiotics, siderophores, signaling molecules, and virulence factors shape ecology and disease within the airways of people with cystic fibrosis.

## Graphical abstract

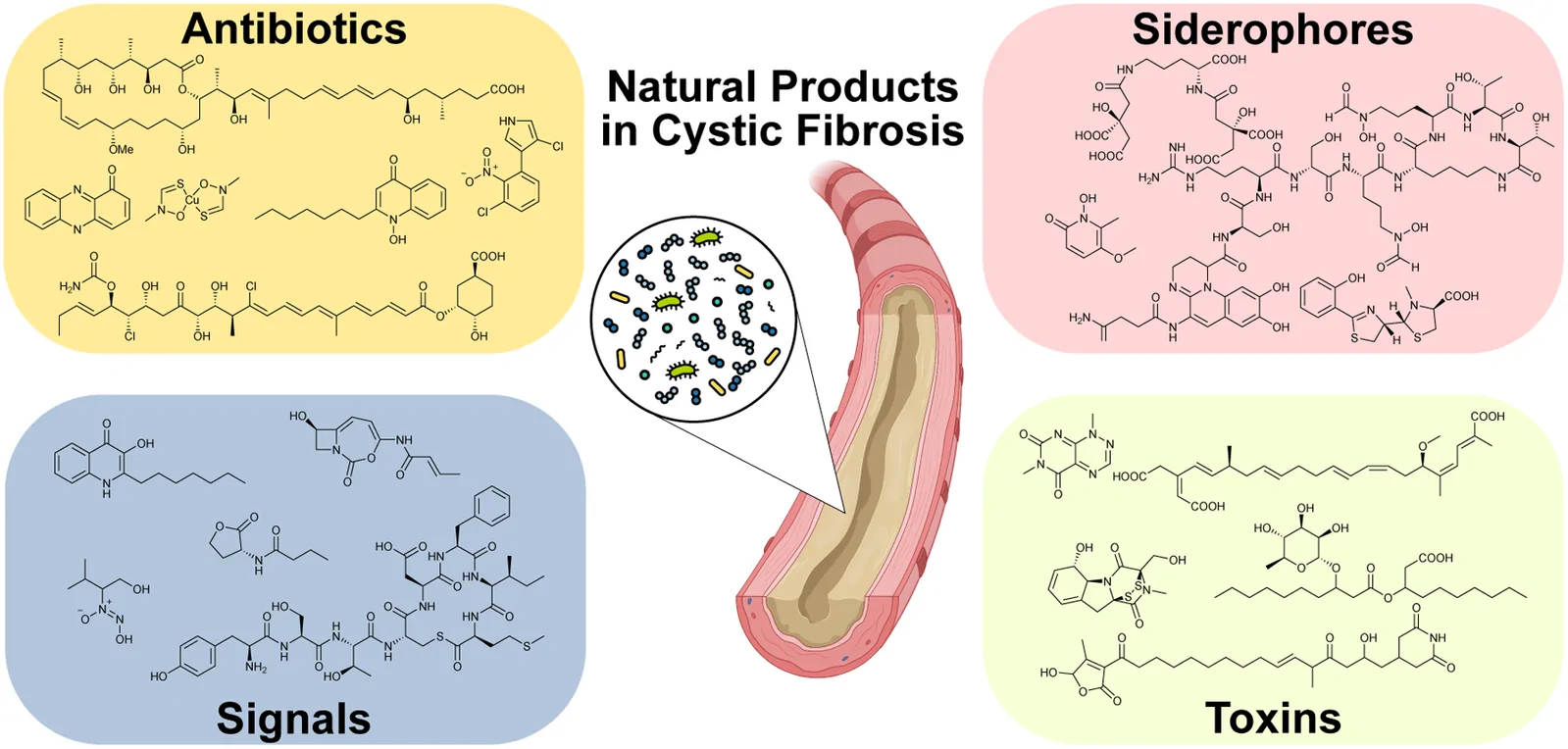

## 1. Introduction

As of 2025, approximately 188 000 individuals worldwide are living with cystic fibrosis (CF).^1^ CF is caused by mutations in the *CF transmembrane conductance regulator* (CFTR) gene, which lead to the accumulation of dehydrated, viscous mucus in the airways.^2,3^ This mucus provides a niche in which pathogens establish chronic infections that result in respiratory exacerbations, lung failure, and death.^4^ Despite recent advances in treatment, CF remains a life-shortening disease with substantial morbidity and mortality, reflected in a median age at death of 37 years.^5^ CFTR modulators have transformed care for many people with CF (pwCF), but approximately 10% of patients cannot access or tolerate these therapies. Further, chronic polymicrobial infection can persist in pwCF taking modulators.^6–8^

The composition of the airway microbiota varies across pwCF and over the lifespan, but *Pseudomonas aeruginosa*, *Staphylococcus aureus*, and *Aspergillus fumigatus* are among the most prominent and well-characterized pathogens that colonize the airways.^9–13^ Rarer pathogens, including members of the genera *Burkholderia*, *Achromobacter*, *Scedosporium*, *Pandoraea*, and *Pantoea*, have emerged more recently as opportunistic colonizers of the lower respiratory tract in pwCF.^8,9,14–16^

To colonize the lower airways, microbes must attach to the epithelial surface, accommodate or evade the host immune system, cope with decreased access to freely available nutrients, and interact with other resident microbes.^17,18^ As one means to mediate these processes, microbes produce bioactive natural products.^19^ These compounds include antibiotics, siderophores, signaling molecules, and toxins that influence how microbes interact with each other, acquire nutrients, and damage host tissues.^19,20^ Within the context of CF, most known natural products are produced by a relatively small number of pathogens (*e.g.*, *Aspergillus fumigatus*, *Burkholderia* spp., *Pseudomonas aeruginosa*, and *Staphylococcus aureus*), which we review below. However, the known biosynthetic repertoire of even these well-characterized pathogens is almost certainly incomplete.

Several lines of evidence indicate that natural product production by CF pathogens is relevant *in vivo*. For instance, natural products produced by *P. aeruginosa* have been detected directly in sputum from pwCF, often at high micromolar concentrations.^21^ Furthermore, disrupting the biosynthetic pathways for these natural products often attenuates colonization of the airways, sensitizes the pathogens to the immune system, or reduces virulence.^22–25^ While some of these compounds have been established as critical for pathogen colonization or virulence in the airways of pwCF, the functions and roles of most CF natural products remain unknown.

Beyond these well-characterized pathogens, genome sequencing of CF-associated isolates has revealed numerous natural product biosynthetic gene clusters (BGCs) whose cognate products remain unknown.^26^ The CF airway is therefore not only a setting in which known natural products can be assigned biological functions, but also a reservoir for natural product discovery. Identifying these natural products, and linking individual molecules to pathogen persistence and virulence, will be necessary for a fuller understanding of polymicrobial infection in CF and may also reveal new opportunities to develop therapeutics that disrupt compounds critical for colonization or harness the antibiotics these microbes synthesize.^20,27^

## 2. Natural products produced by the cystic fibrosis airway microbiota

Below, we describe the known natural products isolated from members of the CF microbiota, detected in sputum *ex vivo*, or whose BGCs are encoded in the genomes of microbes isolated from pwCF. For natural products that are well-characterized, we summarize the literature on their biosynthesis and biological activity. For more recently described natural products or those without extensive literature, we provide a detailed overview of their discovery and what is known and unknown about their potential roles in the CF airway. We have grouped these natural products into four broad categories: (1) antibiotics and antifungals, (2) siderophores and other metallophores, (3) signaling molecules, and (4) toxins and other potential virulence factors. These categories are used only for organizational clarity, and several natural products could reasonably fit into more than one of the above categories, which we will note as needed. Within each category, the natural products we highlight are organized alphabetically.

### 2.1. Antibiotics and antifungals

Structurally diverse natural products with antibiotic and/or antifungal activity are produced by CF pathogens (Fig. 1). These compounds mediate interspecies competition between different members of the microbiota and may help their producers to establish themselves and maintain persistence in the CF airway, ultimately shaping the composition of the microbiota. Here, we discuss natural products with described antibiotic and/or antifungal activity produced by the CF microbiota, their biosynthesis, mechanisms of action, and minimal inhibitory concentrations (MICs) against relevant organisms, when known. For ease of comparison, we have converted MIC values into micromolar concentrations.

**Fig. 1:**
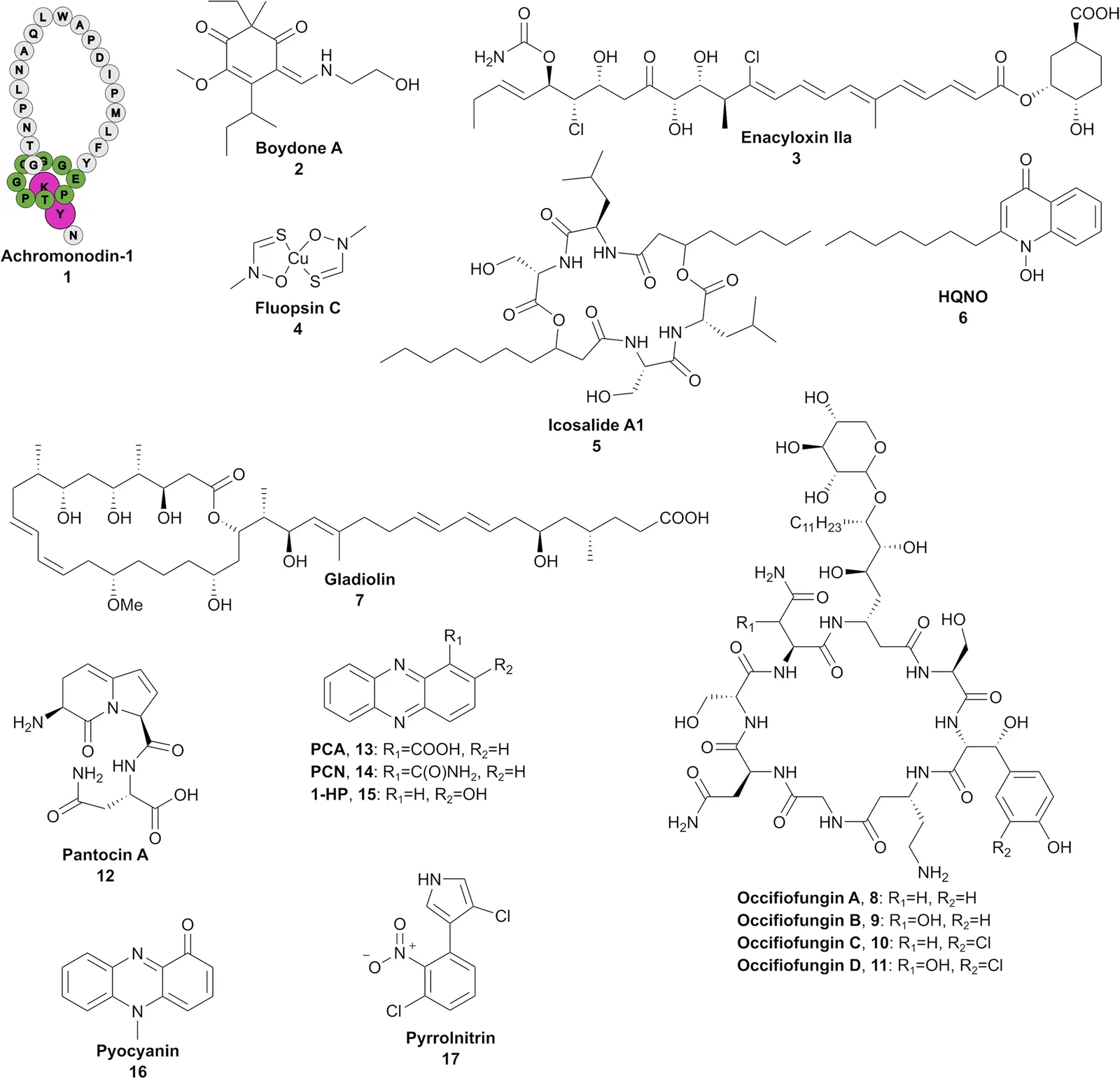
Structures of antibiotic and antifungal natural products produced by CF pathogens. Structure of 1 adapted from Carson *et al.*^28^ Each circle in 1 represents a single amino acid, with the corresponding one-letter amino acid code indicated. Loop and tail residues are shown in grey, ring residues in green, and steric lock residues in pink.

#### 2.1.1. Achromonodin-1

The genomes of multiple CF sputum isolates of *Achromobacter xylosoxidans* encode a lasso peptide-type ribosomally synthesized and post-translationally modified peptide (RiPP) BGC designated *acr1A*.^28^ This BGC contains genes for the predicted precursor peptide Acr1A, a protease Acr1B, a cyclase Acr1C for installation of the lasso peptide isopeptide bond, and the ATP-binding cassette exporter Acr1D.^28^ Carson *et al.* isolated Achromonodin-1 (1) by heterologously expressing the *acr1A* BGC in *Escherichia coli*, placing *acr1A* under an IPTG-inducible promoter and *acr1BCD* under constitutive promoters. Addition of IPTG led to accumulation of achromonodin-1 in the culture supernatant.^28^ 2D NMR revealed that the structure of achromonodin-1 is a characteristic lasso peptide with rotaxane structure, featuring an exceptionally large loop of 20 amino acid residues and a short tail of 2 amino acid residues. This compound contains an eight-membered macrolactam ring formed by an isopeptide bond between Gly-1 and Glu-8, with Lys-28 and Tyr-29 serving as steric lock residues that maintain the threaded conformation of the peptide.^28^

Achromonodin-1 resembles the lasso peptide ubonodin, which was recently characterized from *Burkholderia ubonensis*.^29^ Ubonodin exhibits antibiotic activity against members of the *Burkholderia cepacia* complex (*Bcc*) and inhibits RNA polymerase *in vitro*.^29^ Similarly, achromonodin-1 inhibits transcription in a dose-dependent manner and possess antibiotic activity against the closely related strain *Achromobacter pulmonis* (MIC: 1.3 µM), but was inactive against *Achromobacter animicus* and *Achromobacter insolitus*.^28^ Lasso peptides tend to possess narrow–spectrum activity against bacteria that are closely related to the producing organism.^30^ In the case of achromonodin-1, this activity spectrum is likely driven by its inability to access RNA polymerase, as an *E. coli imp4213* mutant with a more permeable outer membrane was inhibited by this lasso peptide (MIC: 2.5 µM).^28^ Multiple species of *Achromobacter* are known to infect the CF airway.^14^ It is possible that achromonodin-1 is used by *A. xylosoxidans* to compete with these other species. The achromonodin-1 receptor in *A. pulmonis* is not currently known, making predictions on the activity spectrum not feasible at this time.^28^

#### 2.1.2. Boydone A

Given widespread antagonistic interactions between *A. fumigatus*, *P. aeruginosa*, and other bacteria in the airways of pwCF, it was thought that the emerging fungal pathogen *Scedosporium* may also produce antibiotics to compete against bacteria in this environment. Staerck *et al.* found that culture supernatant from a CF sputum isolate of *Scedosporium boydii* exhibited antibiotic activity against *S. aureus*, including eight methicillin-resistant (MRSA) strains, *Staphylococcus epidermidis*, *Streptococcus agalactiae* and *Streptococcus pyogenes*.^16^ Through bioactivity guided fractionation and structural characterization, the active compound was determined to be the six-membered cyclic polyketide (PK) boydone A (2).^16^ The MIC of this compound against these bacteria was not reported, though the bacterial supernatants produced zones of inhibition ≥20 mm on agar plate assays. Boydone A was previously isolated from *Pseudallescheria boydii* (syn. *S. boydii*) growing on decaying leaves of *Sesuvium portulacastrum*, but antibiotic activity was not reported and the compound was inactive against A549 lung cancer cells.^31^ Additional structurally similar PKs have been isolated from other fungi, but antibiotic activity has not been reported for these compounds.^16^ The role of boydone A in the CF airway is unknown, as most prior studies have focused on the antifungal effect of CF bacteria on *Scedosporium* spp.^32^

#### 2.1.3. Enacyloxin IIa

Enaclyoxin IIa (3) is a polyene first identified in a CF isolate of *Burkholderia ambifaria* while screening of members of the *Bcc* for antimicrobial activity against CF pathogens.^33^ The molecule is a product of an unusual modular polyketide synthase (PKS) BGC named *Bamb* that includes both *cis*- and *trans*-acyltransferase (AT) subunits.^33^ The biosynthesis of this compound is typical for a type I PKS, with the exception that only two of the ten modules contain acyltransferase (AT) domains. Thus, it is thought that these two AT domains act in *trans* to load malonyl-CoA onto the acyl carrier protein (ACP) domains in the other modules.^33^ Further deviating from the norm, the final module does not contain a thioesterase (TE) domain. However, this module contains a non-elongating ketosynthase (KS^0^) domain that transfers the fully assembled PK chain onto the peptidyl carrier protein (PCP) domain of Bamb_5917.^34^ Subsequently, the condensation-like domain of the non-ribosomal peptide synthetase (NRPS) Bamb_5915 catalyzes condensation of the thioester with (1*S*,3*R*,4*S*)-3,4-dihydroxycyclohexane carboxylic acid, which releases the PK chain. Several post-PKS tailoring steps that involve two chlorination events and one carboxylation event then yield the final products enacyloxin IIa and its isomer *iso*-enacyloxin II (Fig. 2).^33^

**Fig. 2:**
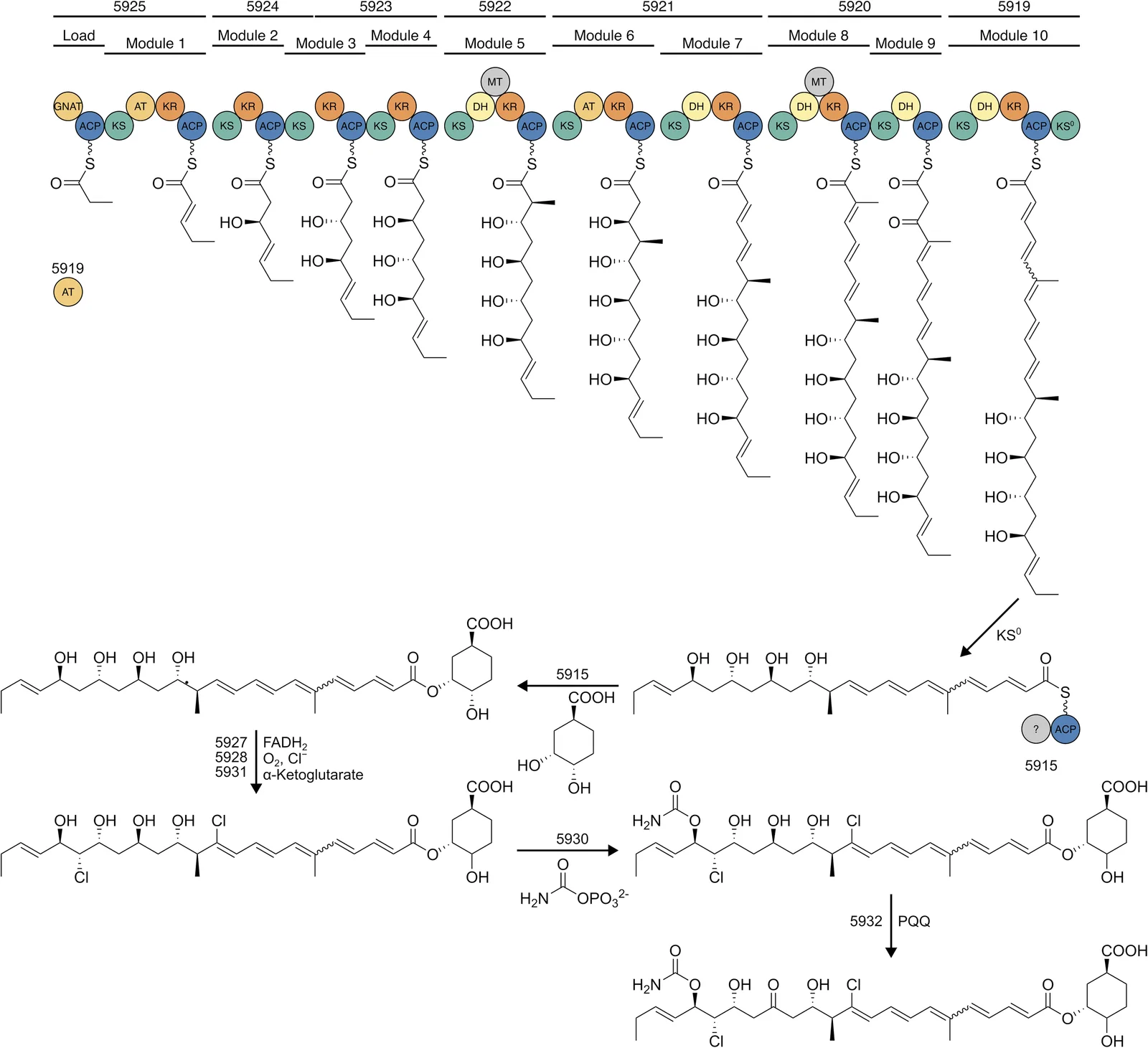
Proposed pathway for enacyloxin IIa biosynthesis. Domain and module organization of the enacyloxin hybrid *cis*-AT/*trans*-AT PKS showing the proposed structure of each ACP-bound intermediate. Proteins and modules are labeled. The proposed mechanisms for post-PKS tailoring reactions are shown below the assembly line. Domain abbreviations: ACP, acyl carrier protein; AT, acyltransferase; DH, dehydratase; GNAT, GCN5-related *N*-acetyltransferase-like domain; KR, ketoreductase; KS, ketosynthase; KS^0^, acyl carrier protein transacylase; MT, methyltransferase. Based on a figure from ref. 33 under the terms of the CC BY 3.0 license.

Enaclyoxin IIa is known to inhibit translation by binding to a pocket in elongation factor-Tu and interfering with GTP hydrolysis and release from the ribosome.^35^ Enacyloxin IIa is active against members of the *Bcc*, including *B. multivorans* (MIC: 9.3 µM) and *B. dolosa* (MIC: 10.7 µM), and *Acinetobacter baumannii* (MIC: 4.3 µM). Similarly, *iso*-enacyloxin IIa is also active, albeit at higher concentrations, against *B. multivorans* (MIC: 20 µM), *B. dolosa* (MIC: 21.3 µM), and *A. baumannii* (MIC: 10 µM).^33^ Furthermore, enacyloxin IIa has been shown to inhibit the sexually transmitted pathogen *Neisseria gonorrhoeae*.^36^ Although, *N. gonorrhoeae* is not found in the airways of pwCF, oropharyngeal *Neisseria* spp. are commonly found in this environment.^37^ This suggests that enaclyoxin IIa could influence colonization of pathogens and oropharyngeal bacteria in the CF airway.

#### 2.1.4. Fluopsin C

Transcriptional profiling of *P. aeruginosa* under copper stress revealed induction of a putative BGC, which contains genes encoding the FlcA methyltransferase, the adenylosuccinate lyases FlcB and FlcC, and two enzymes with heme oxygenase domains called FlcD and FlcE.^38^ By comparing the metabolomes of wild-type *P. aeruginosa* and *flc* mutants cultured under high copper concentrations, the product of this BGC was identified and determined to be fluopsin C (4).^39^ This copper-containing natural product was first identified in environmental isolates of *Pseudomonas* and *Streptomyces*,^39^ but the biosynthesis was not known. Recently, Patteson *et al.* determined that FlcB conjugates l-cysteine with fumarate, which functions as a protecting group. FlcE then catalyzes both an oxidative decarboxylation and *N*-hydroxylation of l-cysteine.^39^ Subsequently, FlcD catalyzes excision of 2-C from the intermediate as formate and FlcC catalyzes C–S bond cleavage to yield a thiohydroxamate and regenerate fumarate.^39^ FlcA then methylates the intermediate using a methyl group from l-methionine *via* a *S*-adenosyl-methionine (SAM)-dependent mechanism to yield the apo fluopsin product.^39^ Finally, two molecules of fluopsin bind a copper atom to produce fluopsin C (Fig. 3).^39^

**Fig. 3:**
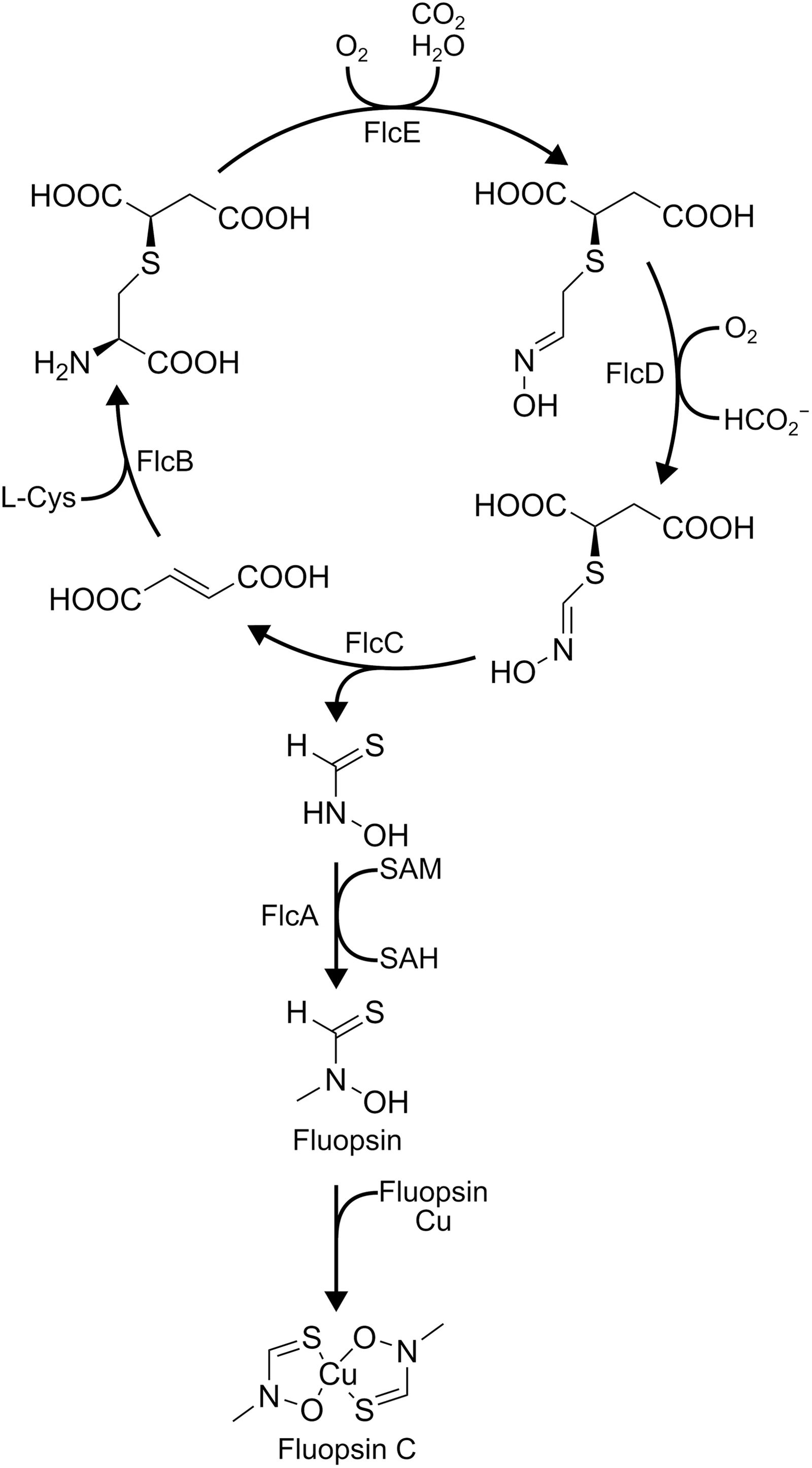
Proposed pathway for fluopsin C biosynthesis. Based on a figure from ref. 39 with permission of the American Association for the Advancement of Science.

Strikingly, the *flc* BGC is conserved in >99% of genomes from clinical and environmental *P. aeruginosa* isolates,^39^ suggesting strong selection for production of this compound, perhaps in mediating interactions with the host or other microbes. For instance, it has been known since the 1970s that fluopsin C functions as a broad-spectrum antimicrobial.^39^ This compound is active against several environmental and pathogenic bacterial species found in the airways of pwCF, including multidrug-resistant *A. baumannii* (MIC: 21.3 to 85.3 µM), *Bacillus subtilis* (MIC: 1.3 to 2.7 µM), *Klebsiella pneumoniae* (MIC: 21.3 µM) and *S. aureus* (MIC: 5.2 to 21.3 µM), but is inactive against *P. aeruginosa* (MIC >300 µM).^39^ Fluopsin C is bactericidal against both Gram-positive and Gram-negative bacteria, and causes thickening and compaction of the cell wall and cytoplasmic membrane detachment.^40,41^ Moreover, fluopsin C inhibits *S. aureus* biofilms (biofilm MIC: 12.8 to 102.5 µM),^40^ which reflects the likely growth mode of this pathogen in the airways. Further, fluopsin C is active against multidrug-resistant *K. pneumoniae* in a murine sepsis model, though it exhibited systematic toxicity (LD_50_ > 4 mg kg^−1^) and moderate hepatotoxicity *in vivo* and exerts cytotoxic (≥8.2 µM) and hemolytic effects (≥8.2 µM) *in vitro*.^40,41^ This compound also exhibits strong antifungal activity against planktonic and biofilm cultures of the yeast pathogens *Candida albicans* (MIC: 6.4 µM) and *Candida auris* (MIC: 3.2 µM),^42,43^ which can also infect the airways of pwCF.^44^ It is not known how fluopsin C influences the composition of the CF airway microbiota. However, during coculture between *P. aeruginosa* and *A. fumigatus* on synthetic CF sputum medium, the *flc* BGC is downregulated.^45^

#### 2.1.5. Icosalide A1

Icosalide A1 (5) was first identified as a lipopeptide antibiotic produced by the fungus *Aureobasidium* spp. and was later rediscovered in a *Burkholderia gladioli* endosymbiont of the beetle *Lagria villosa*.^46,47^ It is likely that this compound was actually produced by a *B. gladioli* symbiont of *Aureobasidium* spp.^47,48^ More recently, production of icosalide A1 was also confirmed in a *B. gladioli* isolate from a pwCF.^48^ This compound is produced by a large four-module NRPS called IcoA. Notably, IcoA contains starter unit condensation (*C*_S_) domains in both its first and its third module, indicating a non-canonical biosynthetic pathway.^47^ The starter unit *C*_S_ domain in module one loads an activated β-hydroxy octanoic acid onto the NRPS, followed by standard extension using an l-leucine residue. A dual condensation/epimerization domain in module 2 then catalyzes epimerization of the *N*-acyl-leucinyl thioester and extension with l-serine. The *C*_S_ domain in module three is thought to independently load an activated β-hydroxy decanoic acid onto the peptidyl carrier protein (PCP)-bound l-seryl. Subsequently, condensation of the two *N*-acylated intermediates results in formation of a depsipeptide, which is then extended with an l-leucine residue. Finally, the thioesterase domain in module four catalyzes product release *via* an intramolecular cyclization resulting from nucleophilic attack of the β-hydroxy octanoyl moiety on the peptidyl thioester to yield the bislactone icosalide A1 (Fig. 4).^47,48^ This compound is reported to be weakly active against *Enterococcus faecalis* and *Streptococcus pneumoniae*, but specific MIC values are not reported. Moreover, icosalide A1 weakly inhibits *Enterococcus faecium* (MIC: 22.4 µM), and *Streptococcus pyogenes* (MIC: 11.2 to 22.4 µM), the epimerization of the l-leucine is critical for activity, as the structural analogues icosalide A2 and B, which only contain l-amino acids are inactive against these pathogens.^46^ Oral streptococci are common inhabitants of the airways of pwCF, and there is increasing evidence that *Enterococcus* can colonize this environment as well.^49,50^ Perhaps *B. gladioli* uses icosalide A1 to compete against enterococci and streptococci in the CF airway.^47^

**Fig. 4:**
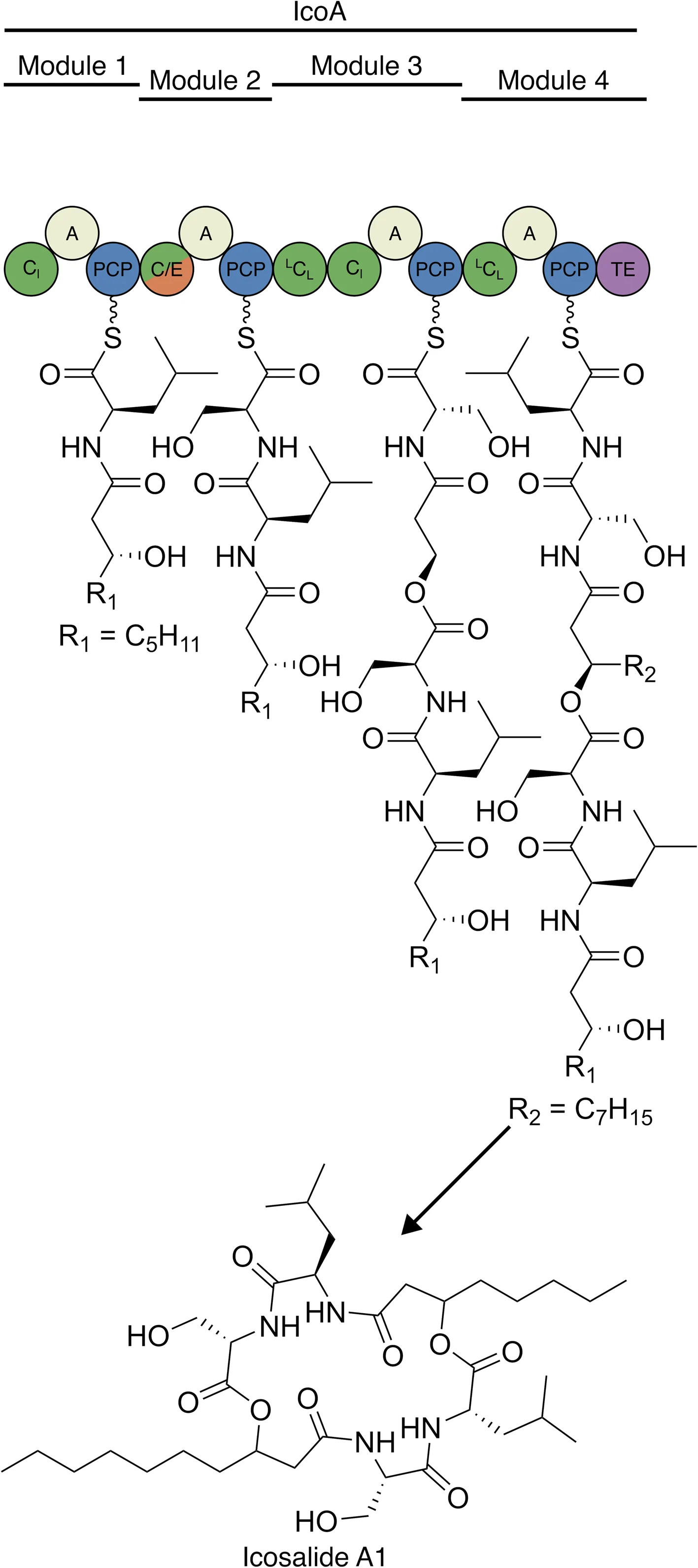
Proposed pathway for icosalide A1 biosynthesis. Domain and module organization of the icosalide A1 NRPS showing the proposed structure of each PCP-bound intermediate. Proteins and modules are labeled. Domain abbreviations: A, adenylation; C/E; dual condensation/epimerization domain; ^L^C_L_, condensation domain linking an l-amino acid to a peptide ending with an l-amino acid; C_l_, chain initiating condensation; PCP, peptidyl-carrier protein. Based on a figure from ref. 48 under the terms of the CC BY-NC 3.0 license.

#### 2.1.6. 2-Heptyl-4-hydroxyquinoline *N*-oxide

2-Heptyl-4-hydroxyquinoline-*N*-oxide (HQNO) (6) was first identified in *P. aeruginosa* culture filtrate that antagonized streptomycin activity and inhibition of bacterial cytochrome-dependent respiration.^51^ HQNO was later identified as an anti-staphylococcal factor from the sputum sample of pwCF.^52^ This natural product is derived from the biosynthetic pathway for production of *Pseudomonas* quinolone signal (PQS) and its precursor 2-heptyl-4(1*H*)-quinolone.^53^ We will describe the biosynthesis of these natural products below (see 2.3.4 below). In addition to functioning as intraspecies signaling molecules (see 2.3.4 below), intermediates in the PQS pathway mediate microbe–microbe and host–microbe interactions. HQNO contains a quinoline ring, a hydroxyl group, an *N*-oxide, and a heptyl group, which enable this compound to bind to the quinone-binding site of respiratory enzymes.^54^

HQNO facilitates a myriad of interactions between *P. aeruginosa* and *S. aureus*. For instance, HQNO inhibits oxidation of cytochrome *b*_1_ and reduction of cytochrome *a*_2_, which are essential components of the electron transport chain critical for aerobic respiration in *S. aureus*.^55^ HQNO binds to the quinone reduction site of cytochrome *bc*_1_ complex and inhibits the oxidase activity of cytochrome *bo*_3_, *bd*, and the type II NADH-quinone oxidoreductase, which together inhibits electron transfer and respiration.^56,57^ Recently, Filkins *et al.* determined that during prolonged coculture production of PQS, along with the siderophores pyochelin (see 2.2.8 below) and pyoverdine (see 2.2.9 below), by *P. aeruginosa* kills *S. aureus*.^58^ In this case, HQNO poisons the electron transport chain, and the siderophores scavenge iron that is essential for cytochrome function. HQNO also activates the SrrAB two-component system, resulting in a shift in *S. aureus* from aerobic metabolism toward fermentative metabolism. As a byproduct of fermentation, *S. aureus* releases lactate, which *P. aeruginosa* consumes preferentially over glucose.^58,59^ Consequently, over time, decreased fermentation efficiency and continued competition with *P. aeruginosa* lead to the death of *S. aureus* and potentially its reduced abundance in the airways of pwCF.^58^ Subinhibitory concentrations of HQNO can increase *S. aureus* persistence by promoting the growth of small colony variants that are better able to survive in the presence of *P. aeruginosa*,^58,60^ or induce production of antioxidant pigment staphyloxanthin (see 2.4.9 below). Furthermore, HQNO represses the activity of the SaeRS two-component system, which is involved in the production of numerous *S. aureus* virulence genes.^61^ The airways of younger pwCF are colonized primarily by *S. aureus*, and *P. aeruginosa* is only present at low relative abundance. In contrast, *P. aeruginosa* often colonizes the airways of older pwCF.^62,63^ It is thought that production of HQNO by *P. aeruginosa* may in part explain the inverse associations between these two pathogens in the CF airway.^52^

HQNO may also exert antibiotic activity against other members of the CF lung microbiota.^52,54,55^ To persist in this environment, other bacteria have evolved mechanisms to detoxify HQNO. For instance, *Mycobacteroides abscessus* produces an *O*-methyltransferase that detoxifies HQNO by targeting the *N*-hydroxy group and converting the compound to the less potent 2-heptyl-1-methoxy-4(1*H*)-quinolone.^56,57^*O*-Methyltransferases produced by mycobacteria may provide protection against the toxic effects of HQNO in the airways of pwCF.

#### 2.1.7. Gladiolin

During a screen of *Burkholderia* isolates, Song *et al.* determined that a *B. gladioli* isolate from a pwCF, produces broad-spectrum antimicrobial activity against Gram-positive bacteria and yeasts, including *C. albicans*.^64^ Through a bioactivity guided purification approach, the active PK macrolide gladiolin (7) was identified. Gladiolin is synthesized by the *gbd rans*-AT PKS.^64^ The biosynthesis of gladiolin is typical for a *trans*-AT PKS, but the compound features two unusual β-methyl branches that are predicted to be installed on the PK backbone using a hydroxymethylglutaryl-CoA synthase/enoyl-CoA hydratase cassette consisting of GbnFH, GbnIJ, and GbnRS (Fig. 5).^64^

**Fig. 5:**
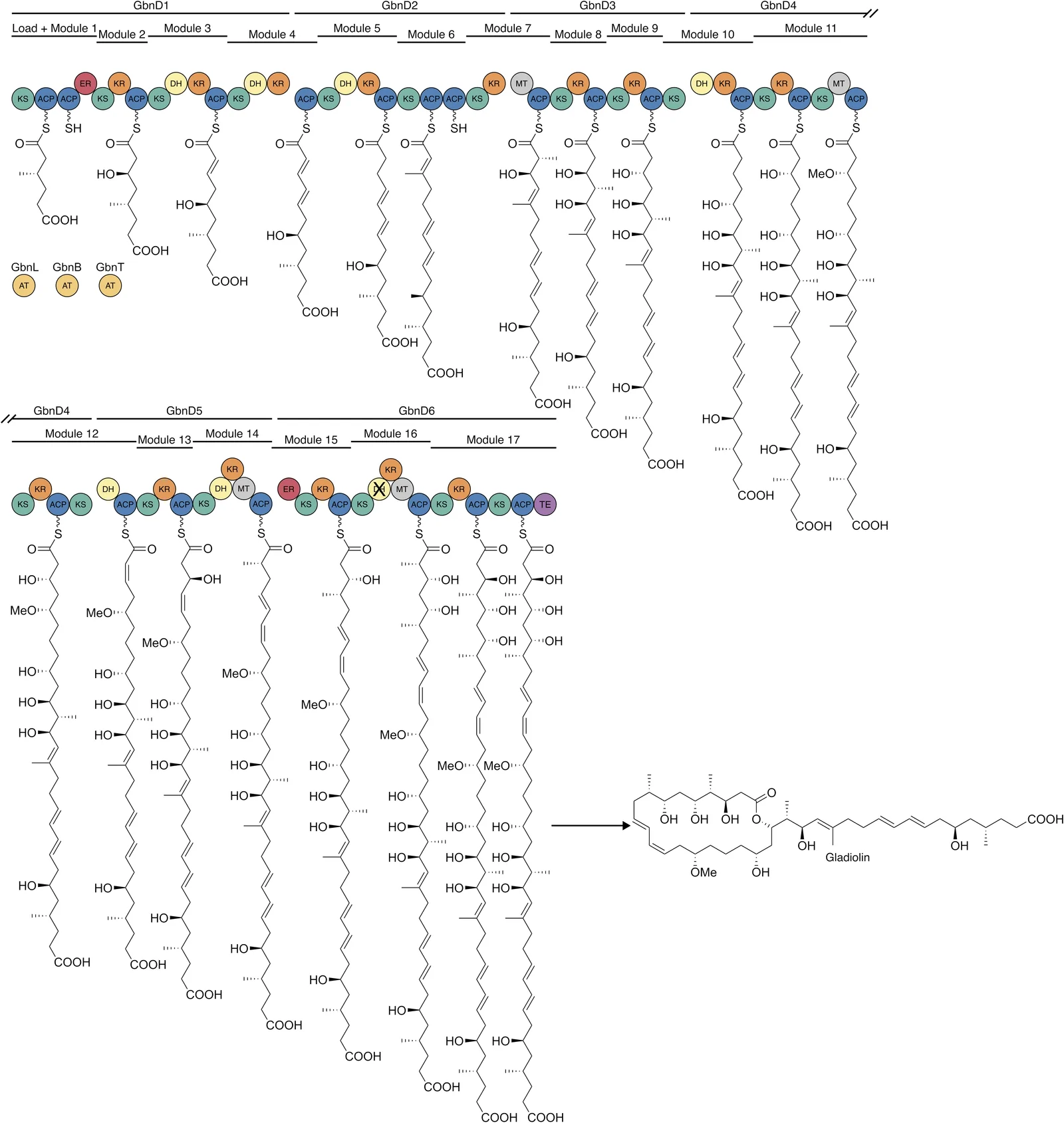
Proposed pathway for gladiolin biosynthesis. Domain and module organization of the gladiolin *trans*-AT PKS showing the proposed structure of each ACP-bound intermediate. Proteins and modules are labeled. KS12, KS14, and KS20 are predicted to be non-elongating. Domains that are predicted to be inactivated are marked with an X. Domain abbreviations: ACP, acyl carrier protein; AT, acyltransferase; DH, dehydratase; ER, enoyl reductase; KR, ketoreductase; KS; MT, methyltransferase; TE, thioesterase. Based on a figure from ref. 64 under the terms of the CC BY 4.0 license.

Gladiolin is not active against Gram-negative bacteria, but does exhibit activity against *N. gonorrhoeae* (MIC: 1.3 to 2.6 µM) *S. aureus* (MIC: 10.3 µM) and *C. albicans* (MIC: 5.1 µM).^36,64^ Gladiolin is structurally similar to etnangien, a natural product previously characterized from the myxobacterium *Sorangium cellulosum*.^65^ Etnangien is active against Gram-positive bacteria, including *Mycobacterium* spp. (MIC: 0.14 to 1.2 µM) and *S. aureus* (MIC: 1.2 µM). This compound directly inhibits RNA polymerases *in vitro*, suggesting that the cell envelope may act as a barrier in Gram-negative bacteria and fungi.^66,67^ Like etnangien, gladiolin inhibits RNA polymerase and is active against *Mycobacterium tuberculosis*, including isoniazid- and rifampicin-resistant strains (MIC: 0.52 to 3.0 µM).^64^ Etnangien is unstable due to the presence of a labile hexaene-containing side chain attached to the C-21 of the macrolide core, which is inactive without this moiety.^66^ Gladiolin, in contrast, contains a shorter and more saturated side chain that presumably makes it more stable than etnangien.^64^

There is an inverse correlation between *B. gladioli* and *Candida* in the airways of pwCF.^68,69^ In a follow-up study gladiolin did not possess activity against *C. albicans,* in contrast to the first report. However, exposure to the compound inhibited hyphal growth, shifting *C. albicans* to the yeast form.^70^ Simm *et al.* determined that gladiolin synergizes with the ergosterol-binding antifungal PK amphotericin B against *C. albicans in vitro* and in an *in vivo* model system for ventilator-associated pneumonia.^70^ Gladiolin interacts with yeast membrane lipids to enhance amphotericin B binding and exacerbates fungal membrane damage.^70^ Though there are no known producers of amphotericin B in the airways of pwCF, it is possible that gladiolin may synergize with another membrane-targeting natural product produced by a member of the CF airway microbiota to inhibit *C. albicans* or other fungi in this environment.

#### 2.1.8. Occidiofungin

Occidiofungins (8 through 11) are cyclic glycopeptides that were originally isolated from an environmental strain of *Burkholderia contaminans*.^71,72^ The hybrid PKS-NRPS *ocf* (sometimes called *occ*) BGC responsible for production of occidiofungin is found in multiple members of the *Bcc*.^72,73^ The biosynthesis of occidiofungin begins with production of a unique C18 β-hydroxy fatty amino acid, which is generated by the PKS-like OcfH, followed by β-hydroxylation and addition of an amino group by OcfI and OcfG, respectively.^74^ This lipid serves as the starter unit for assembly of an octapeptide scaffold *via* the canonical NRPS OcfABCDE.^74,75^ This NRPS incorporates the non-proteinogenic amino acids 2,4-diaminobutyric acid and β-hydroxytyrosine, which may also be halogenated to produce additional variants of the compound.^74^ Following macrocyclization and release from the assembly line, OcfC installs a xylose residue to the compound to yield the occidiofungins (Fig. 6).^75^

**Fig. 6:**
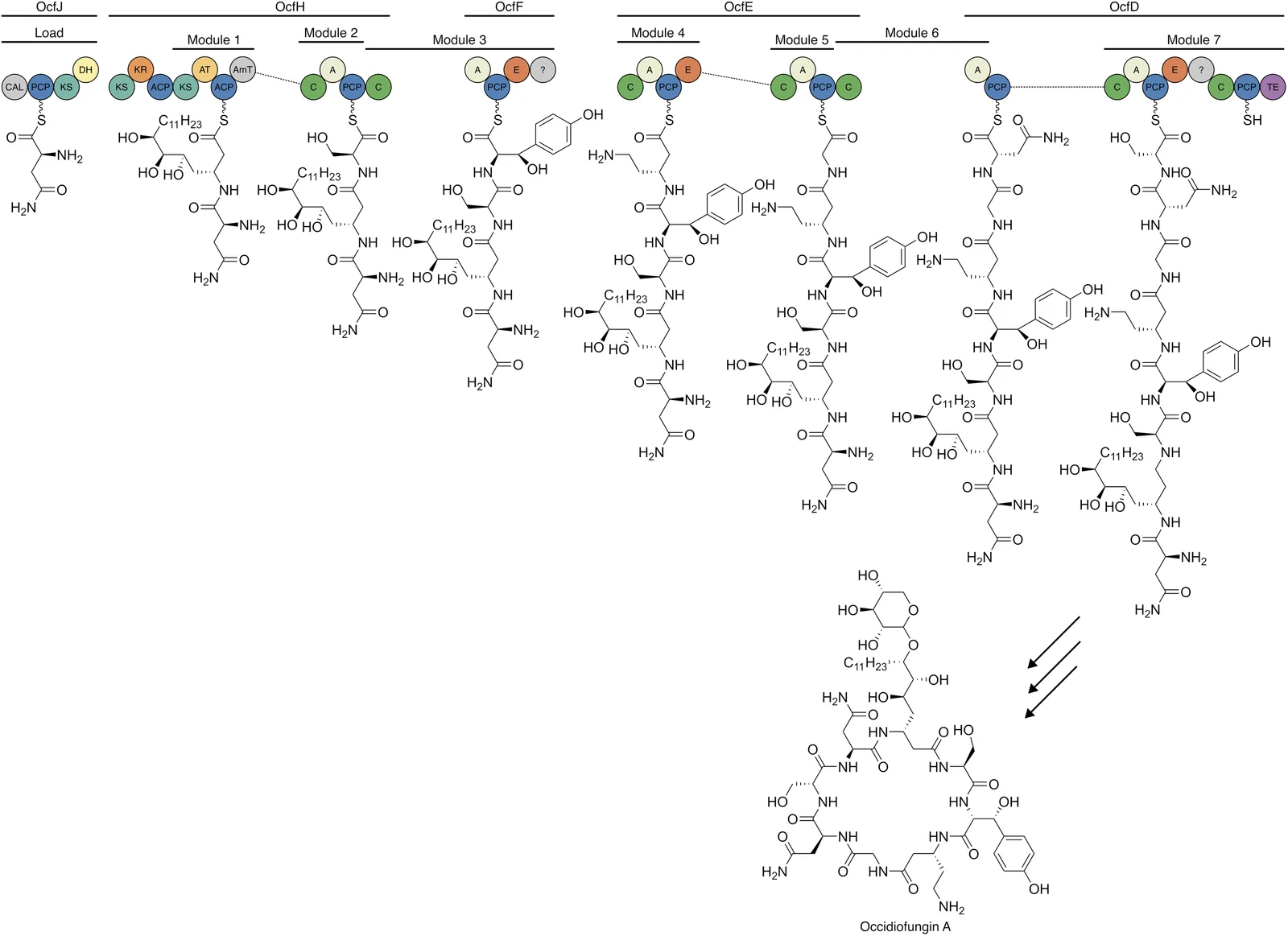
Proposed pathway for occidiofungin A biosynthesis. Domain and module organization of the occidiofungin hybrid PKS-NRPS showing the proposed structure of each ACP/PCP-bound intermediate. Proteins and modules are labeled. Dashes indicate modules that are part of the same polypeptide. The PKS domains in OcfJ are thought to be inactive. The final product depicted is occidiofungin A. Other variants are also produced. Domain abbreviations: A, adenylation; ACP, acyl carrier protein; AT, acyltransferase; AmT, aminotransferase; CAL, co-enzyme A ligase; C, condensation; DH, dehydratase; E, epimerization; KR, ketoreductase; KS; PCP, peptidyl carrier protein; TE, thioesterase.

Occidiofungin A (8) is active against several filamentous fungi and yeast, including *Aspergillus* spp. (MIC: 1.7 to 3.3 µM), *Candida* spp. (MIC: 0.4 to 0.8 µM), *Fusarium* spp. (MIC: 3.3 µM), *Geotrichum candidum* (MIC: 6.7 µM), and *Mucor* spp. (MIC: 3.3 to 6.7 µM).^71,72,76,77^ This compound targets actin, inhibits endocytosis, nuclear segregation, and hyphal formation, ultimately leading to apoptosis and cell death.^73,78^

Though the *ocf* BGC is not found commonly in human pathogenic *Burkholderia* spp., it was recently detected within the genomes of two isolates of *B. contaminans* from blood culture and sputum of a pwCF in Argentina.^74,79^ Notably, *B. contaminans* is more frequently isolated from pwCF in Ibero-American countries, and its role in the CF airway is not well-understood.^79,80^

#### 2.1.9. *Pantoea* natural products 1 (pantocin A) and 2

While screening a collection of *Pantoea* isolates for antibacterial activity, Robinson *et al.* found that strain *Pantoea agglomerans* Tx10, isolated from the sputum of a pwCF, exhibited broad-spectrum inhibition against multiple bacteria, including several members of the CF microbiota.^15^ Transposon mutants of *P. agglomerans* that lost activity against *Staphylococcus hemolyticus* had disruptions in a putative six gene BGC designated *pnp2* for *Pantoea* Natural Product 2 (PNP-2). This BGC encodes a putative arylamine *N*-acetyl transferase Pnp2a, a serine hydrolase Pnp2b, a major facilitator superfamily transporter Pnp2c, a *p*-aminobenzoate *N*-oxygenase Pnp2d, a ferredoxin reductase Pnp2e, and an ATP-grasp protein Pnp2f. Based on these predicted gene functions, PNP-2 may include a *p*-nitrobenzoate starter unit like aureothin from *Streptomyces thioluteus*.^81^ However, the structure of PNP-2 and its mechanism of action are yet to be determined.^15^

In addition to PNP-2, Robinson *et al.* determined that *P. agglomerans* Tx10 also produces an antibiotic called pantocin A (also known as *Pantoea* Natural Product 1) (12), which was first discovered in a plant-associated strain of *P. agglomerans*.^15,82^ Pantocin A is an unusually small three amino acid RiPP that inhibits histidine biosynthesis.^82^ After the thirty amino acid precursor peptide PaaP is synthesized by the ribosome, PaaA performs a double cyclization-condensation and decarboxylation between two glutamate residues in the core peptide to form a bicyclic core.^83^ PaaB is an α-ketoglutarate-dependent iron oxidase capable of performing a two-electron dehydrogenation, which may function alongside PaaA or may be involved in another downstream biosynthetic step. Though the protease that cleaves the modified core peptide from the leader and follower peptides is unknown, it is known that PaaC is an efflux pump required for export and self-resistance to the mature pantocin A.^83^ Using a combination of disruption mutants, Robinson *et al.* suggest that PNP-2 is more broadly active, inhibiting most *E. coli* and other enteric pathogens and *Staphylococcus* spp., while pantocin inhibits subsets of *Enterobacter* and *Pantoea* strains and is active against some enteric pathogens, including *Erwinia amylovora*.^15^ Enteric bacteria are sometimes found in the airways of pwCF, but whether these bacteria are inhibited *in vivo* by *P. agglomerans* is not known.

#### 2.1.10. Phenazines

Phenazines are nitrogen-containing heterocyclic natural products that are redox-active and are produced by a myriad of bacteria, including *Pseudomonas* spp. and *Burkholderia* spp.^84^ These natural products have been reviewed extensively.^84–87^ Therefore, we will focus our discussion on their biosynthesis and the potential roles specific phenazines play in the airways of pwCF.

Phenazines are produced using a conserved BGC designated as *phz*.^88^ In *P. aeruginosa*, two homologous BGCs, *phz1* and *phz2*, each contribute to phenazine production.^88,89^ Biosynthesis begins when PhzE uses l-glutamine as an amine donor to convert chorismic acid to 2-amino-2-deoxyisochorismic acid (ADIC).^90^ The isochorismatase PhzD hydrolyzes ADIC to form *trans*-2,3-dihydro-3-hydroxyanthranilic (DHHA).^91^ Subsequently, the isomerase PhzF tautomerizes and rearranges DHHA to form 6-amino-5-oxocyclohex-2-ene-1-carboxylic acid (AOCHC).^91,92^ PhzA and/or PhzB catalyze head-to-head condensation of two molecules of AOCHC, which forms the phenazine scaffold hexahydrophenazine-1,6-dicarboxylic acid (HHPDC).^92,93^ Subsequent oxidation, involving a flavin-dependent enzyme PhzG and other spontaneous reactions, generates phenazine-1,6-dicarboxylic acid (PDC).^84^ This intermediate undergoes decarboxylation to yield phenazine-1-carboxylic acid (PCA) (13).^94,95^ PCA serves as a central precursor for several phenazine derivatives,^85^ including phenazine-1-carboxamide (PCN) (14), 1-hydroxyphenazine (1-HP) (15), 2-hydroxyphenazine, and pyocyanin (16) (Fig. 7).^89,96^

**Fig. 7:**
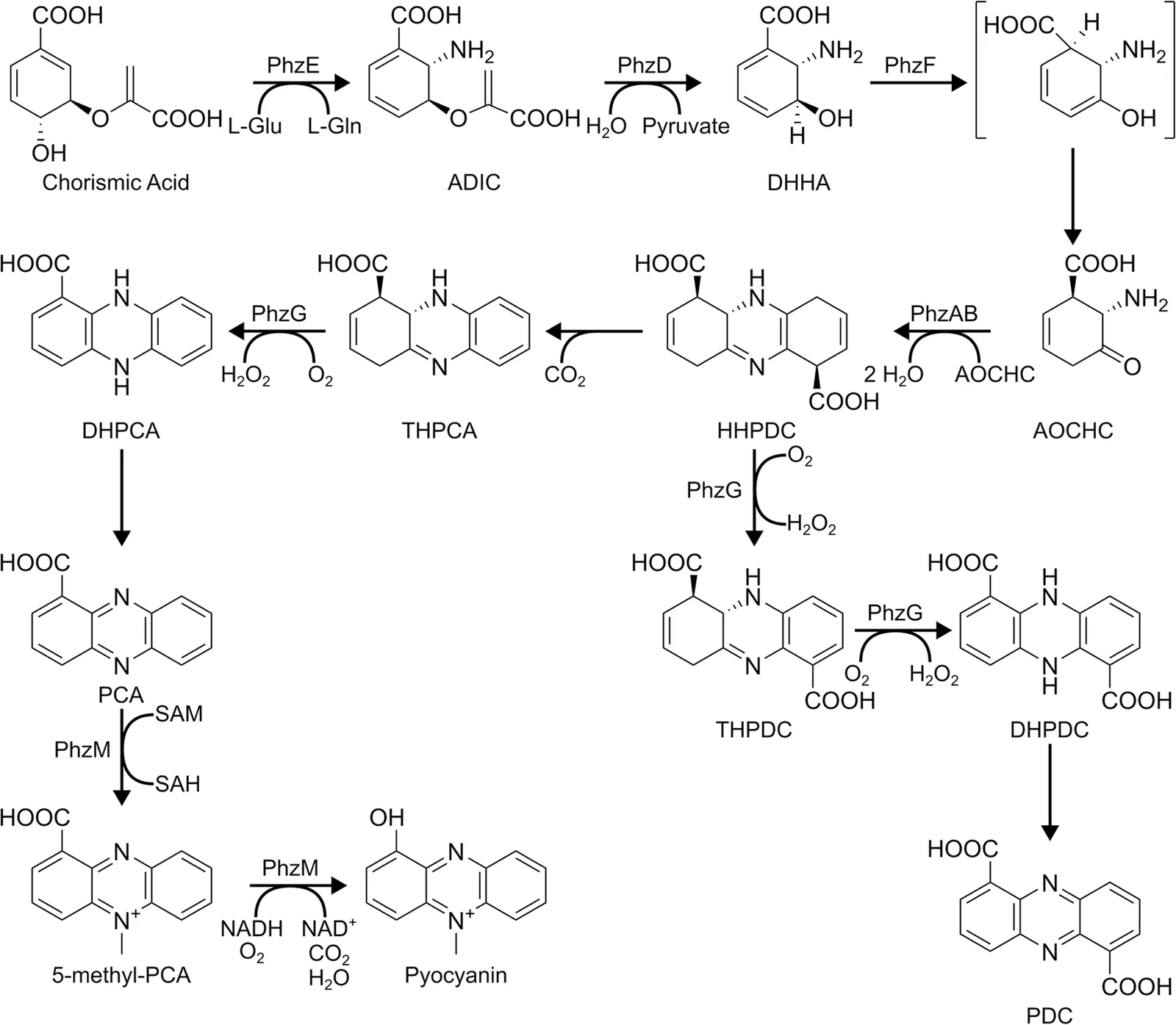
Proposed pathways for biosynthesis of various phenazines by *P. aeruginosa*. Short-lived intermediates are shown in brackets.

All phenazine derivatives are known to exhibit weak antibiotic activity, which may contribute to *P. aeruginosa* persistence and dominance in the airways of pwCF.^97,98^ For instance, pyocyanin inhibits several bacteria and fungi.^99,100^ Pyocyanin exhibits broad activity against several strains of *S. aureus* and several Gram negative bacteria, including *Acinetobacter* spp., *Enterobacter cloacae*, *E. coli*, *H. influenzae*, and *K. pneumoniae*.^98^*Micrococcus luteus* is among most susceptible Gram-positive bacteria against pyocyanin (MIC: 9.5 µM).^99^ 1-HP is the most active phenazine against *A. fumigatus* (MIC: 125 µM) compared to phenazine-1-carboxamide (MIC: 250 to 500 µM) and pyocyanin (MIC: 2000 µM). The mechanism of action of the phenazines involves the production of reactive oxygen species (ROS).^84,101^ Many of these effects may be physiologically relevant, as phenazine in sputa from pwCF have been reported to reach concentrations of approximately 150 µM.^102^

When cultured on airway epithelial cells, *P. aeruginosa* produces phenazines that are reduced *via* oxidation of glutathione and NADH, leading to increase in oxidative stress levels.^87^ The reduced phenazine reacts with free oxygen in the lungs to generate ROS *via* redox cycling.^87^ Production of hydrogen peroxide (H_2_O_2_) causes oxidative stress and cell damage, leading to cell death.^103^ Along with pyocyanin and PCN, PCA and 1-HP, are also capable of inducing ROS production that inhibits the growth of bacteria and fungi.^104,105^ For instance, the pyocyanin biosynthetic precursor, 5-methyl-PCA inhibits *C. albicans* biofilm formation and hyphal growth in a coculture assay.^106,107^ But similar to the effect of phenazines on *A. fumigatus*, the compounds have different biological effects on *C. albicans* depending on the concentration. At high concentrations (>350 µM), phenazines inhibit the growth of *C. albicans*.^105,107^ However, pyocyanin (10 µM) and phenazine-1-carboxylate (60 µM) alter the *C. albicans* respiratory activity resulting in inhibition of colony wrinkling and reduced oxygen exposure.^108^ The MICs of different phenazines against *A. fumigatus* correlate with their redox potentials and the MIC changes with pH.^104^ 1-HP not only facilitate ROS production, but it also chelates iron, thus limiting the metal required for the growth of the fungi which may contribute to its enhanced activity against *A. fumigatus*.^104^ However, subinhibitory concentrations of pyocyanin, PCN and PCA may promotes iron acquisition by fungi, as these compounds reduce insoluble ferric iron to ferrous iron.^104^

Thus, phenazines serve as key factors in microbial competition, giving *P. aeruginosa* a distinct advantage over competing microbes. Beyond their antibiotic activity, phenazines also promote *P. aeruginosa* biofilm formation and iron acquisition. Furthermore, phenazines function as signaling molecules that influence quorum sensing.^109^ Collectively, phenazines enhances the ability of *P. aeruginosa* in the CF airway and promote its persistence and survival.

#### 2.1.11. Pyrrolnitrin

Pyrrolnitrin (17) is a phenylpyrrole antibiotic that was first described in *Pseudomonas pyrrocinia*, but is also produced by *Burkholderia* spp.^110–112^ The *prn* BGC has been identified in *B. ambifaria*, *B. cepacia*, and *B. cenocepacia* isolates from the CF airway, and production of the molecule has been confirmed.^112^ Pyrrolnitrin biosynthesis proceeds *via* PrnA, which performs an FAD-dependent chlorination of l-tryptophan to produce 7-chloro-l-tryptophan, followed by PrnB-dependent decarboxylation and ring rearrangement.^108,113^ Subsequently, PrnC installs a second chlorine atom onto the pyrrolic intermediate *via* FAD-dependent halogenation.^114^ Finally, PrnD catalyzes oxidation of the arylamine group to a nitro group, which yields pyrrolnitrin (Fig. 8).^115^

**Fig. 8:**
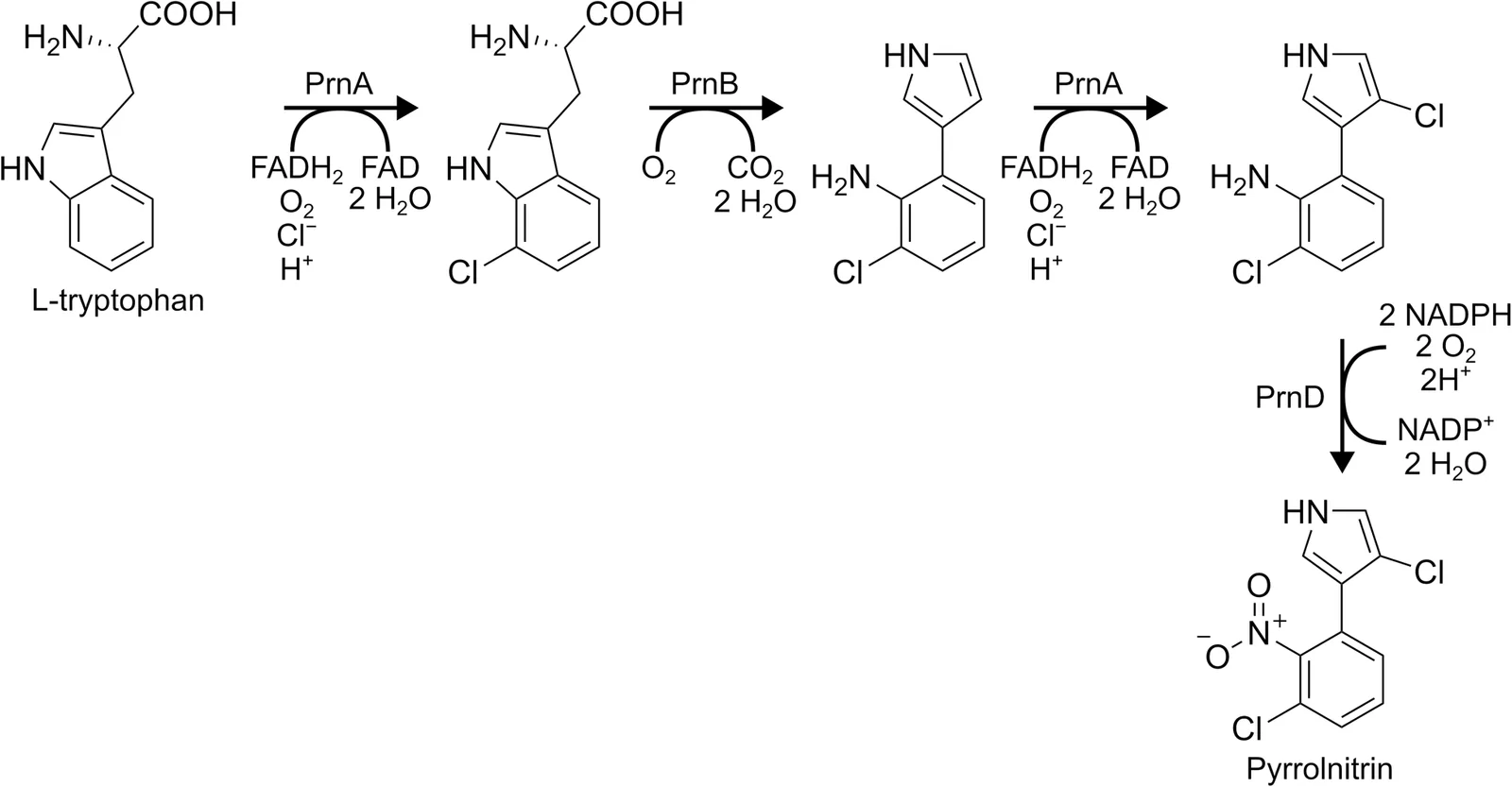
Pathway for biosynthesis of pyrrolnitrin.

Pyrrolnitrin broadly inhibits the electron transport chain in diverse fungi,^112^ blocking electron flow from NADH dehydrogenase (complex I) to succinate dehydrogenase (complex II), preventing reduction of ubiquinone (coenzyme Q) and arresting respiration.^116,117^ This compound also affects various growth stages of fungi *Sclerotinia sclerotiorum*, including apothecial formation, ascospore germination and mycelial growth. There are several other reported instances where pyrrolnitrin suppresses the mycelial growth of the fungi *Fusarium graminearum* and inhibits the spore formation in *A. fumigatus* and *Aspergillus niger*.^118,119^ Though an early report indicated that pyrrolnitrin is not an effective systematic antifungal,^120^ this compound could be active within the airways of pwCF. In addition to its antifungal activity, the molecule is weakly active against the Gram-positive bacteria *S. aureus* (MIC: 195 µM), *B. subtilis* (48.6 µM) and *Mycobacterium* spp. (389 µM), and the Gram-negative bacterium *Salmonella enterica* (MIC: 292 µM).^118,121,122^ Beyond antibiotic activity, pyrrolnitrin could be involved in virulence, as cytotoxic activity has been reported against human liver cancer (IC_50_: 58.4 µM) and glial cells (IC_50_: 97.3 µM).^118^ However, it is not currently known if pyrrolnitrin is active against airway epithelial cells.

### 2.2. Siderophores and other metallophores

As one means to acquire scarce iron from the environment, microbes produce natural products called siderophores. These natural products are released in an iron-free form (also called the desferri-form) from the cell and act as high-affinity iron chelators. The producer will typically also produce a transporter that recognizes the iron-bound (also called the ferri-form) of the natural product and imports it back into the cell. In addition to siderophores, microbes also produce other metallophore natural products that facilitate uptake of other minerals (*e.g.*, copper, nickel, and zinc) that sequester these metals from their environment. To survive in the CF airway, microbes produce structurally diverse siderophores and other metallophores (Fig. 9). Here, we describe known siderophores and other metallophores produced by the CF microbiota and their roles in establishing chronic colonization of the lung.

**Fig. 9:**
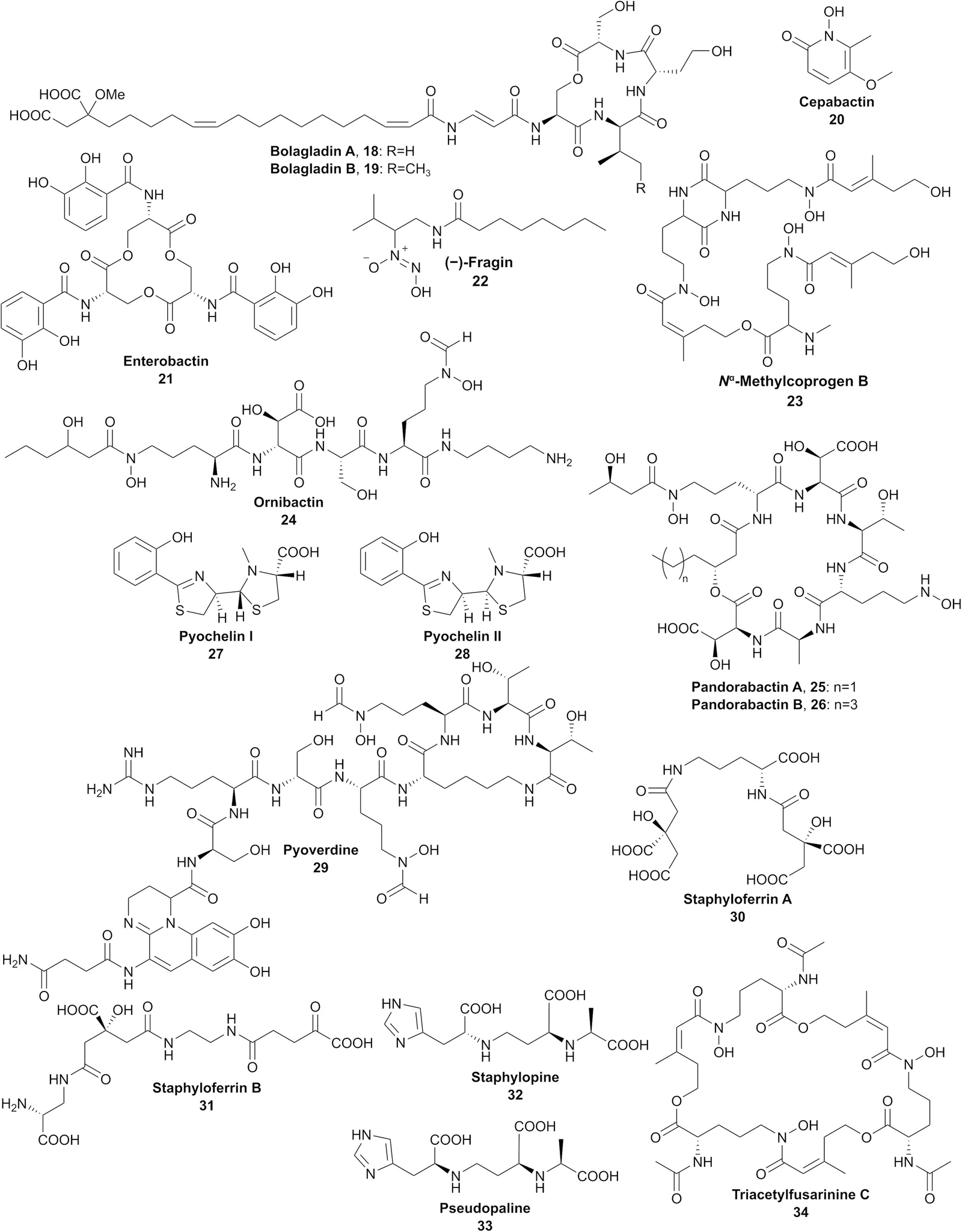
Structures of siderophores and other metallophores produced by the CF microbiota. Structure 29 is pyoverdine E, but other pyoverdine variants are produced by *Pseudomonas* spp.

#### 2.2.1. Bolagladin A and B

When Dashti *et al.* cultured a gladiolin (see 2.1.7 above) biosynthesis mutant of *B. gladioli* on minimal medium they discovered two novel compounds, which they named bolagladin A (18) and B (19) for their bolaamphiphilic properties.^123,124^ These natural products consist of a depsitetrapeptide core and a rare dehydro-β-alanine (Dba) residue that connects the core to an unusual citrate-derived fatty acid.^124^ The *bol* BGC is responsible for the biosynthesis of these compounds and includes a multimodular NRPS named BolH and a citrate synthase homologue named BolR. The biosynthesis of the bolagladins is thought to proceed first through assembly of a citryl-CoA starter unit followed by elongation by a β-ketoacyl synthase III, thought to be either BolM or BolP, followed by two desaturation events. BolO is predicted to adenylate the β-carboxyl group of l-aspartic acid, which is then transferred onto the freestanding acyl carrier protein BolC. This intermediate undergoes PLP-dependent decarboxylation and subsequent tailoring reactions associated with attachment of the citrate-derived lipid. The thioester intermediate is then desaturated by BolQ to generate the Dba-derived linkage. Subsequently, the NRPS BolH installs the tetradepsipeptide core to yield the bolagladins (Fig. 10).^123,124^

**Fig. 10:**
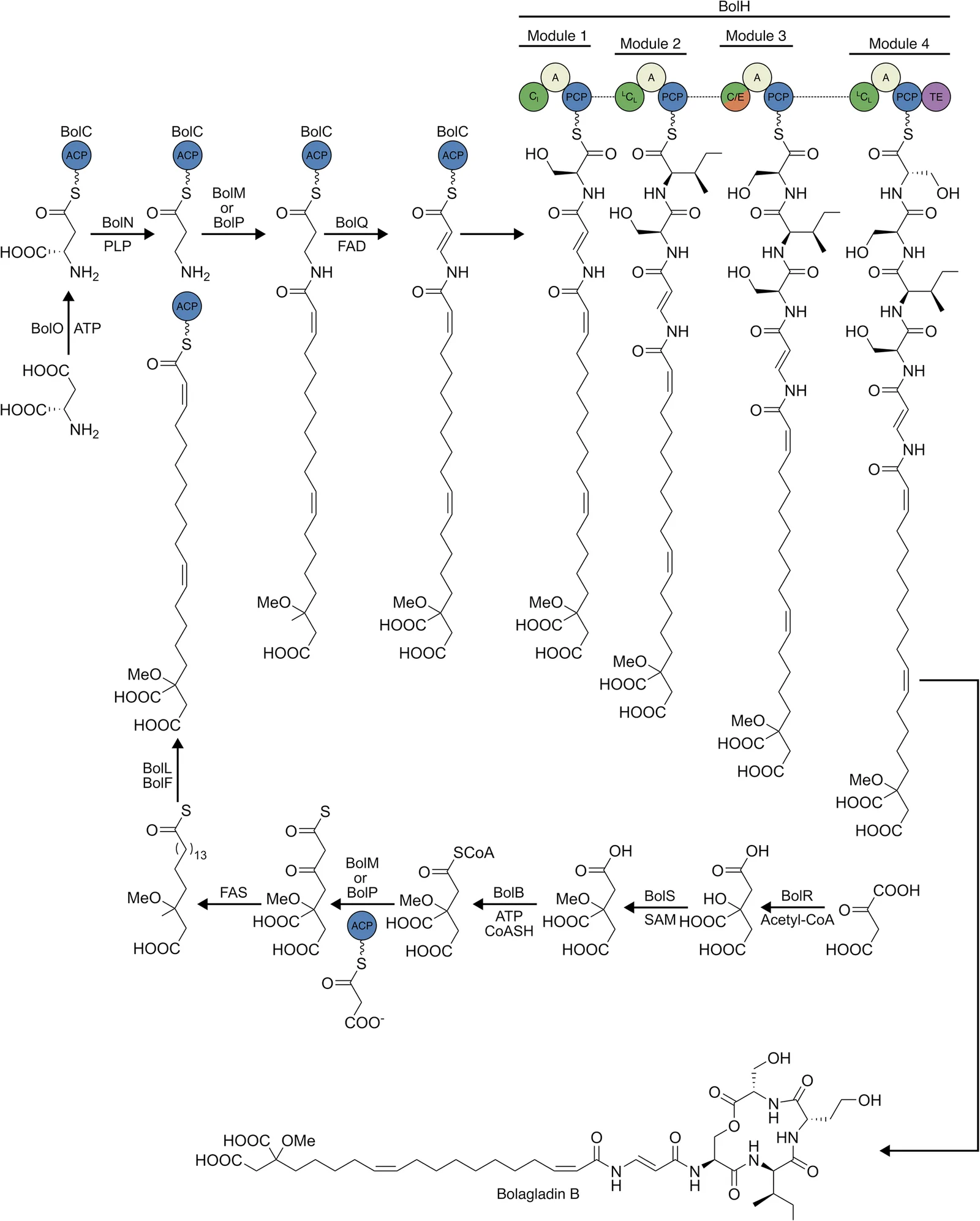
Proposed pathway for bolagladin biosynthesis. Domain and module organization for the bolagladin NRPS showing the proposed structure of each PCP-bound intermediate. Proteins and modules are labeled. Dashes indicate modules that are part of the same polypeptide. It is thought that the *O*-methylation of the citrate-derived starter unit occurs early, but this has not yet been confirmed. The final product depicted is bolagladin B. Other variants are also produced. Domain abbreviations: A, adenylation; ACP, acyl carrier protein; C/E; dual condensation/epimerization domain; C_l_, chain-initiating condensation; ^L^C_L_, condensation domain linking an l-amino acid to a peptide ending with an l-amino acid; FAS, fatty acid synthase; PCP, peptidyl-carrier protein; TE, thioesterase. Based on a figure from ref. 124 under the terms of the CC BY 4.0 International license.

There are contradictory reports on the bioactivity of the bolagladins from two different groups. In one report, bolagladin A and B each lack antimicrobial activity against *E. faecium*, *S. aureus*, *K. pneumoniae*, *A. baumannii*, *P. aeruginosa*, and *Enterobacter* spp. (also known as the ESKAPE pathogens), *Mycobacterium bovis*, or *C. albicans*.^124^ However, an independent study reported that bolagladin B inhibited the growth of the bacteria *B. subtilis*, *E. coli*, *E. faecalis*, *Mycobacterium vaccae*, and *S. aureus* and fungi *C. albicans* and *Penicillium notatum*. However, no MIC values were reported.^123^ Both bolagladin A and B were active in the chrome azurol S assay, indicating that they can chelate iron and may function as siderophores.^124^ The *bol* BGC is widely conserved across *B. gladioli*. However, there was no difference between the virulence of wild-type *B. gladioli* and an isogenic bolagladin biosynthesis mutant in a waxworm *Galleria mellonella* virulence model.^124^ It is still not know what role the bolagladins play in the CF airway or if they facilitate iron uptake by *B. gladioli*.

#### 2.2.2. Cepabactin

Cepabactin (20) is a hydroxamate siderophore that was first identified in phytopathogenic *B. cepacia* strains.^125^ This compound has since then also been confirmed to be produced by CF isolates of *B. cepacia*.^126^ Compared to other siderophores produced by *Burkholderia* spp. (see ornibactin and pyochelin, 2.2.6 and 2.2.8, respectively, below), the biosynthesis of cepabactin is poorly understood and there currently is no defined BGC.^127^ This compound chelates ferric iron in a 3-to-1 ratio, but exhibits relatively low iron affinity when compared to other siderophores. However, a specific Fe-binding constant has not been reported.^125,128^ In addition to its iron-chelating activity, cepabactin is structurally similar to two antibiotics, G1549 and BN227, which were discovered from *Pseudomonas alcaligenes*^125,129^ and *Pseudomonas* sp. BN227 (a strain that is similar to *B. cepacia*),^125,130^ respectively. Both G1549 and BN227 exhibit antibiotic activity against a wide range of microbes, including *S. aureus* and *P. aeruginosa*.^125,129,130^ Presumably, cepabactin plays a role in iron acquisition in the CF airway, but this has yet to be established. Further, cepabactin has been demonstrated to be imported by both *B. cenocepacia* and *P. aeruginosa* as a xenosiderophore,^131,132^ which may promote their persistence in the airways of pwCF.

#### 2.2.3. Enterobactin

Enterobactin (21) is a catecholate siderophore that is commonly produced by Gram-negative enteric bacteria, including *E. coli* and *Salmonella typhimurium*.^133^ However, enterobactin production was recently reported by *Rothia mucilaginosa*.^134^ This Gram-positive bacterium is found primarily in the oral cavity and upper respiratory tract, but is also a prevalent member of the CF airway microbiota.^63^ The *ent* BGC in *R. mucilaginosa* is not similar to the corresponding BGC in enteric bacteria,^134^ suggesting that this compound may have arisen convergently in these different bacterial lineages. Though the biosynthesis of enterobactin in *R. mucilaginosa* has not been explicitly described, it likely precedes by conversion of chorismate into 2,3-dihydroxybenzoic acid (DHB) followed by condensation to l-serine *via* an NRPS, and intermolecular cyclization of three DHB-serine to yield enterobactin.^134–136^ Presumably, enterobactin facilitates iron acquisition and is important for the prevalence of *R. mucilaginosa* in the CF airway, but this has yet to be demonstrated. Intriguingly, enterobactin inhibits the growth of oral *Streptococcus* spp., which are also common inhabitants of the CF airway, and inhibited production of staphyloxanthin (see 2.4.9 below) by *S. aureus*.^134^

#### 2.2.4. (−)-Fragin

During an investigation to identify an antifungal activity that was previously known to be produced by a CF isolate of *B. cenocepacia*, Jenul *et al.* identified a diazeniumdiolate-containing natural product named (−)-fragin (22).^137^ This compound was originally discovered from *Pseudomonas fragi* in 1967 and has since then been isolated from several bacteria, including members of the *Bcc* and *Pseudomonas* spp.^137,138^ The *ham* BGC for (−)-fragin biosynthesis is arranged into two diverging operons, *hamABCDE* and *hamFG*.^139^ The biosynthesis of (−)-fragin is still not fully understood, but is thought to proceed from adenylation and thiolation of l-valine by the NRPS-like HamD followed by reductive release of a valine-derived aldehyde. The 2-oxoglutarate-dependent mononuclear iron oxygenase HamA, *N*-oxygenase HamC, and PK cyclase/dehydrase HamE are predicted to convert the aldehyde into a dihydrosydnone *N*-oxide intermediate, which represents a branchpoint in the biosynthesis.^139,140^ A two-electron reduction of this intermediate results in production of the quorum sensing signal molecule valdiazen (see 2.3.5 below), while reductive transamination by HamG followed by acylation by HamF yields (−)-fragin (Fig. 11).^137^

**Fig. 11:**
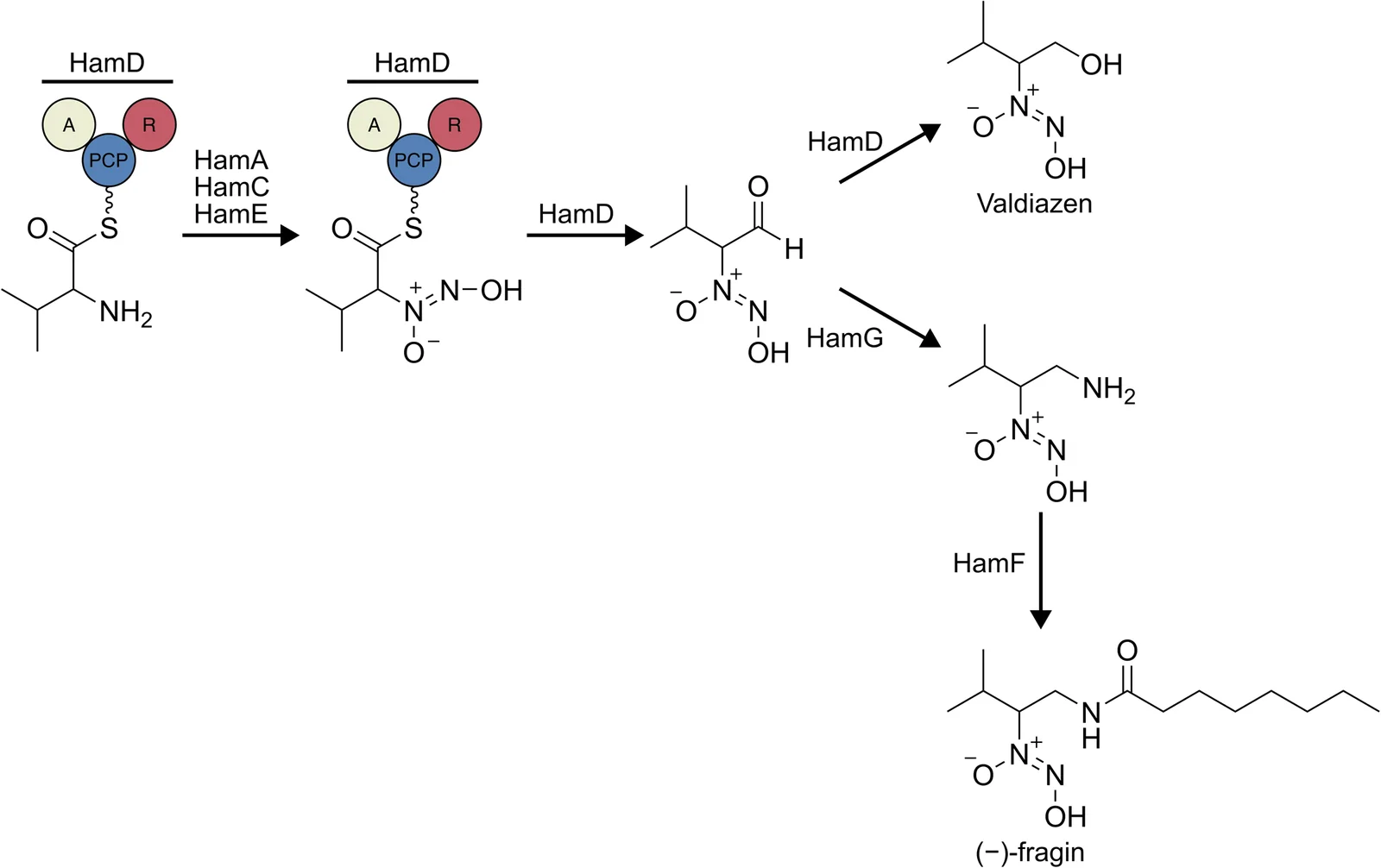
Proposed pathway for (−)-fragin and valdiazen biosynthesis. Domain abbreviations: A, adenylation; PCP, peptidyl carrier protein; R, reductase. Based on a figure from ref. 137 under the terms of the CC BY 4.0 International license.

(−)-Fragin inhibits the growth of Gram-positive bacteria and yeast, but is inactive against Gram-negative bacteria. Diazenium diolate compounds are known to chelate metal ions and the expression of *hamA* increases in iron-depleted cultures. However, Cu-fragin and Fe-fragin complexes lose their antifungal activity.^137^*Saccharomyces cerevisiae* mutants that are defective in metal homeostasis and response to oxidative stress were more sensitive to (−)-fragin-mediated inhibition, suggesting that the inhibitory activity of this natural product is due to metal chelation. However, Cu-fragin complexes retained activity against Gram-positive bacteria, suggesting an alternative mechanism of inhibition. Though the role that (−)-fragin plays in the CF airway is unknown, production of this compound is elevated in *B. cenocepacia* exposed to subinhibitory concentrations of the antibiotic trimethoprim, which is often used to treat infections in pwCF.^141^

#### 2.2.5. *N* ^α^-Methylcoprogen B

*N* ^α^-Methylcoprogen B (23) is a coprogen-type hydroxamate siderophore produced by the fungus *Scedosporium apiospermum*.^142,143^ The biosynthesis of this compound is not characterized in this organism. However, *N*^α^-methylcoprogen B production is dependent upon a homologue of *sidD* that encodes an NRPS involved in the biosynthesis of the hydroxamate siderophores fusarinine C and triacetylfusarinine C (see 2.2.12 below) in *A. fumigatus*.^143,144^ Mutants of *S. apiopsermum* that lack the *sidD* homologue are unable to grow under iron-limited conditions and exhibit loss of virulence in an immunosuppressed murine model of disseminated scedosporiosis.^143^ Though the role of *N*^α^-methylcoprogen B is not known in the CF airway, this compound can scavenge iron from one of the major siderophores, pyoverdine (see 2.2.9 below), produced by *P. aeruginosa*.^86^ This potential iron piracy from pyoverdine may help *S. apiopsermum* establish within the CF airway and explain why these two pathogens are rarely found in the same pwCF.^143^

#### 2.2.6. Ornibactin

Ornibactin (24) is a linear mixed hydroxamate-hydroxycarboxylate siderophore that was first characterized from a *B. cepacia* (previously *P. cepacia*) isolated from rhizosphere soil.^145^ As with cepabactin, production of ornibactin has been confirmed in CF isolates of *B. cepacia*.^126^ The biosynthesis of ornibactin is dependent upon the *orb* BGC. The first step of the biosynthesis involves *N*-hydroxylation of the *N*^*δ*^ of l-ornithine by the flavin-dependent monooxygenase PvdA.^25^ OrbK or OrbL is thought to then transfer a variable-length (C_4_, C_6_, or C_8_) β-hydroxy acid onto this *N*^*δ*^-hydroxy group, which is then loaded onto the OrbI NRPS.^145,146^ Subsequently, biosynthesis proceeds as expected for a linear NRPS. However, the assembly involves incorporation of an unusual l-hydroxyaspartate residue generated by the Fe(ii)/α-ketoglutarate-dependent dioxygenase OrbG followed by epimerization to form d-hydroxyaspartate followed by condensation with l-serine. The intermediate is then transferred to the NRPS OrbJ, which condenses an unusual *N*^*δ*^-formyl-*N*^*δ*^-hydroxyornithine residue, installed by the *N*^*δ*^-hydroxyornithine transformylase PvdF. Finally, chain-release is mediated *via* condensation with putrescine by OrbJ to yield ornibactin (Fig. 12).^127,146^

**Fig. 12:**
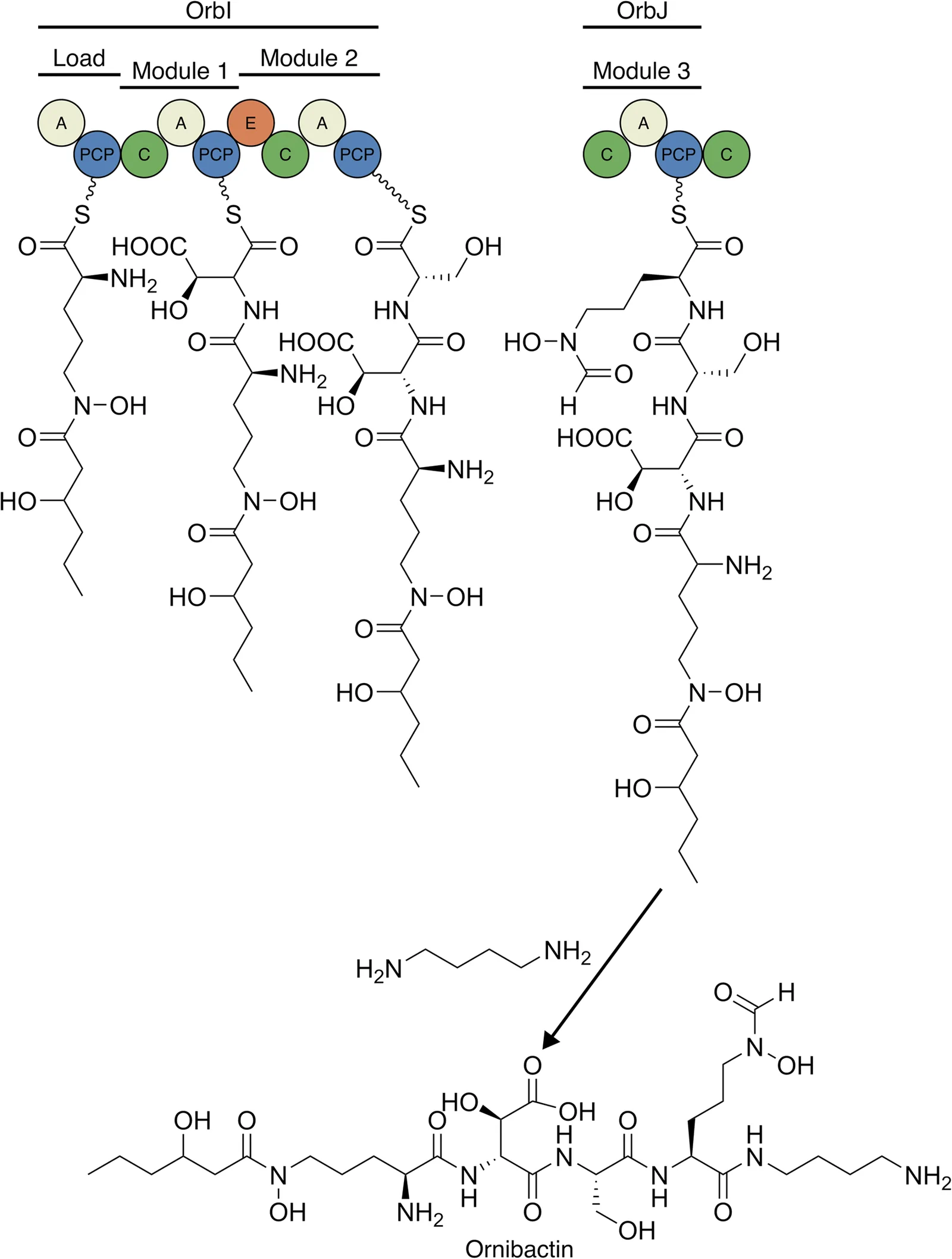
Proposed pathway for ornibactin biosynthesis. Domain and module organization for the ornibactin NRPS showing the proposed structure of each PCP-bound intermediate. Proteins and modules are labeled. Domain abbreviations: A, adenylation; C, condensation; E, epimerization; PCP, peptidyl-carrier protein.

Ornibactin biosynthesis and uptake are critical for colonization of the lung by *Burkholderia* spp. For instance, a *B. cepacia pvdA* transposon-insertion mutants exhibited defects in colonization and virulence in both acute and chronic lung infection models in mice and rats, respectively.^25^ Further, mutants of *B. cenocepacia* with a deletion of *orbA*, which encodes the ferric-ornibactin uptake system, exhibit reduced persistence in a rat lung infection model. Notably, this defect is not rescued by an alternative siderophore, pyochelin (see 2.2.8 below).^147^ Ornibactin may also be used by *Burkholderia* spp. to mediate interspecies interactions and modulate virulence. Ornibactin induces expression of genes in *P. aeruginosa* that are known to be activated under iron-limited conditions, including the virulence factors exotoxin A and PrpL protease.^148–150^ The specific role that ornibactin plays in the CF airway has yet to be determined. However, McAvoy *et al.* recently demonstrated production of the C_8_ form of ornibactin by multiple *Burkholderia* spp. and identified a novel C7 form of ornibactin and C6 analogues of ornibactin from CF strains of *Burkholderia dolosa*.^141^ Together, these studies suggest that ornibactin may contribute to colonization of the CF airway by *Burkholderia* spp. and contribute to virulence during coinfection with other pathogens.

#### 2.2.7. Pandorabactin A and B

Pandorabactin A (25) and B (26) are hydroxamate siderophore products of a highly conserved *pan* BGC found in both environmental and clinical isolates of the emerging CF pathogens *Pandoraea* spp.^151^ The starter unit for pandorbactin biosynthesis is a *N*^*δ*^-3-OH-butyrl-*N*^*δ*^-OH-l-ornithine that is formed by *N*-hydroxylation of l-ornithine by PanF followed by attachment of a rare 3-hydroxy-butyrl moiety by PanQ. After loading onto the PanI NRPS, the fatty acyl-adenylate ligase (FAAL) domain activates and transfers either a C_6_ or C_8_ hydroxy acid, for pandorabactin A and B, respectively, onto the free α-amino group of *N*^*δ*^-3-OH-butyryl-*N*^*δ*^-OH-l-ornithine, followed by typical NRPS extension to generate a cyclized hexapeptide core.^151^ During biosynthesis, the putative dioxygenase PanH likely hydroxylates l-aspartate residues to form l-*erythro*-3-OH-Asp residues (Fig. 13).^151^

**Fig. 13:**
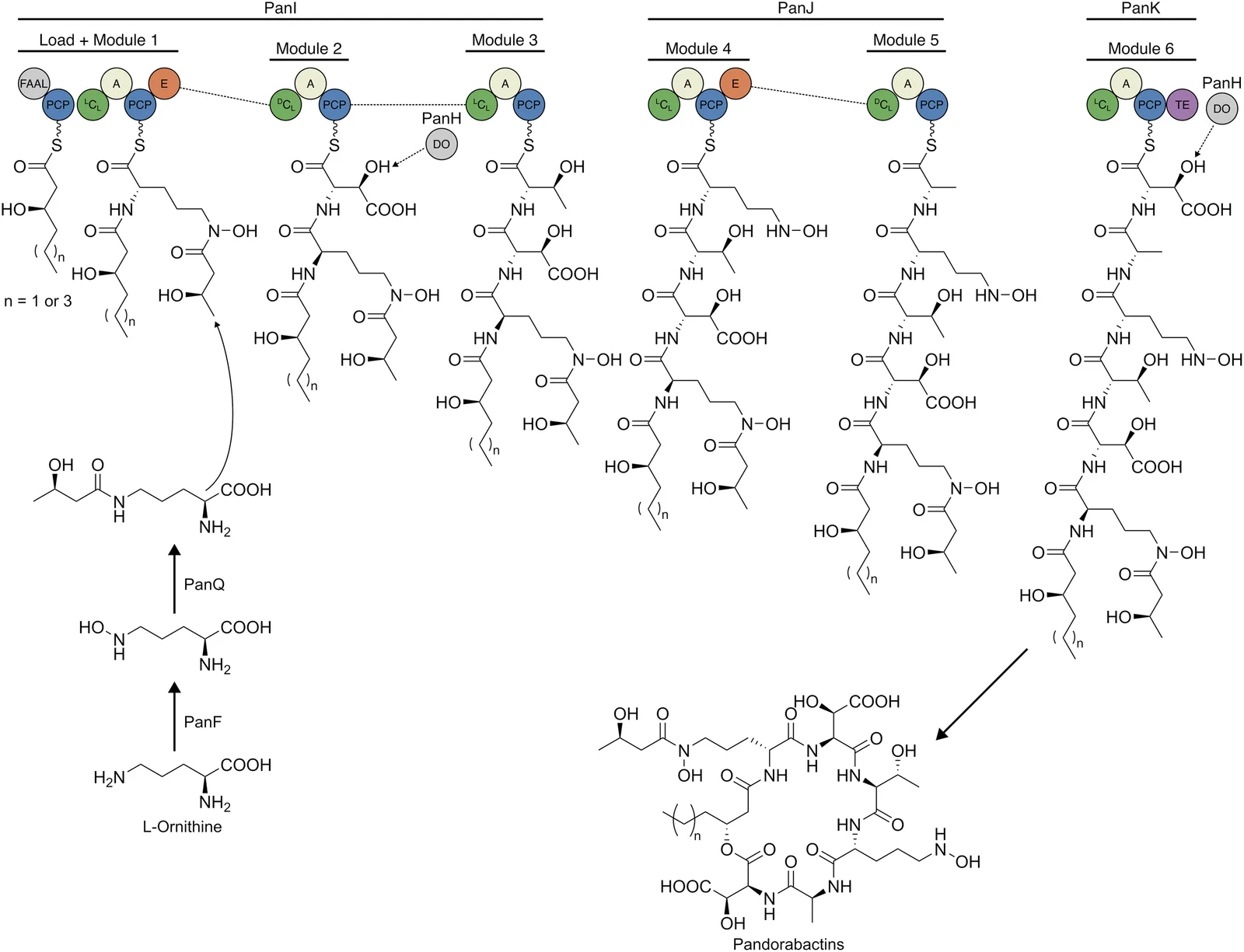
Proposed pathway for pandorabactin biosynthesis. Domain and module organization for the pandorabactin NRPS showing the proposed structure of each PCP-bound intermediate. Proteins and modules are labeled. Dashes indicate modules that are part of the same polypeptide. PanH is predicted to act at two different steps during biosynthesis. Domain abbreviations: A, adenylation; ^L^C_L_, condensation domain linking an l-amino acid to a peptide ending with an l-amino acid; ^D^C_L_, condensation domain linking an l-amino acid to a peptide ending with a d-amino acid; DO, dioxygenase; E, epimerization; FAAL, fatty acyl-AMP ligase; PCP, peptidyl-carrier protein; TE, thioesterase. Figured adapted from ref. 151 under the terms of the CC BY 4.0 International license.

The desferri-forms of pandorabactin A and B each are reported to possess weak antibiotic activity against *M. vaccae* (MIC: 14.0 µM) and *P. aeruginosa* (MIC: 13.5 µM), while the ferri-forms are inactive.^151^ Coculture between *Pandoraea sputorum* and *P. aeruginosa* stimulates production of pyoverdine (see 2.2.9 below) by the latter, suggesting that these siderophores may be involved in iron-mediated exploitation competition between pathogens. In support of this hypothesis, the *pan* BGC has been detected in 8/40 sputum metagenomes, and its presence correlates with the decreased abundance of ten species, with *Stenotrophomonas maltophilia* (MIC: 28 µM) and *Streptococcus mitis* most strongly affected. However, the pandorabactins were inactive against *S. mitis* and *S. pneumoniae*.^151^

#### 2.2.8. Pyochelin

Pyochelin (27, 28) is one of the two major siderophores produced by *P. aeruginosa*.^152,153^ This natural product is also produced by some *Burkholderia* spp.^117^ The biosynthesis of pyochelin is dependent upon the *pch* BGC.^154^ First, the isochorismate synthase PchA and isochorismate pyruvate lyase PchB generate salicylate from chorismate.^155,156^ This salicylate is then adenylated by PchD and loaded onto the NRPS initiation module PchE. The biosynthesis then proceeds with condensation and cyclohydration of one molecule each of l-cysteine by PchE and a second NRPS, PchF, to form thiazoline rings. Subsequently, an NADPH-dependent reductase PchG reduces the second thiazoline to thiazolidine. This reaction introduces a new stereocenter, and pyochelin exists as a mixture of both diastereomers, differing at C-4 of the thiazolidine ring. Finally, the thioesterase domain of PchF releases the intermediate, yielding pyochelin (Fig. 14).^157^ Pyochelin binds to ferric iron with a 2 : 1 stoichiometry with a stability constant of ∼10^5^ M^−1^, which is a lower affinity compared to the other siderophore, pyoverdine (see 2.2.9 below), produced by *P. aeruginosa*. Pyochelin can also chelate zinc and copper, as well as essential micronutrients such as cobalt, molybdenum, and nickel.^158,159^

**Fig. 14:**
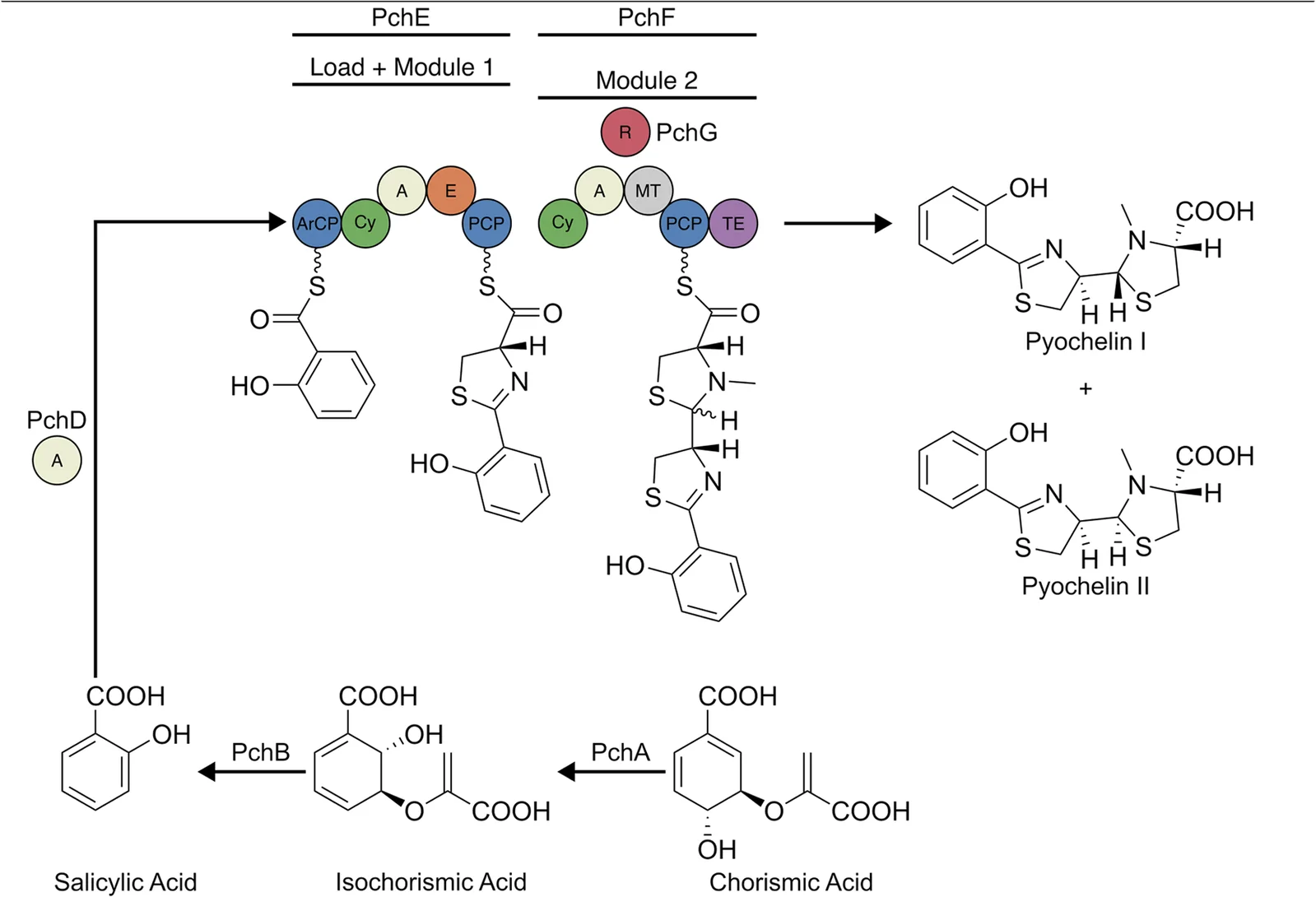
Pathway for pyochelin biosynthesis. Domain and module organization of the pyochelin NRPS showing the proposed structure of each PCP-bound intermediate. Proteins and modules are labeled. Pyochelin exists as a mixture of diastereomers, differing at C-4 of the thiazolidine ring. Proteins and modules are labeled. Domain abbreviations: A, adenylation; ArCP, aryl carrier protein; Cy, cyclization; E, epimerization; MT, methyltransferase; PCP, peptidyl-carrier protein; R, reductase; TE, thioesterase.

The role of pyochelin in *P. aeruginosa* physiology has been well-studied.^153,158,159^ Thus, we will focus on the roles of pyochelin in the CF airway. However, it is important to note, that while expression of the *pch* BGC has been detected in sputum samples from pwCF, pyochelin itself is generally at or below the level of detection (∼1 µM).^160,161^ This suggests that pyochelin is not the primary siderophore produced by *P. aeruginosa* and *Burkholderia* spp. for persistence in the CF airway. Beyond its roles in metal acquisition, pyochelin mediates interspecies interactions between *P. aeruginosa* and other members of the CF lung microbiota. At a concentration of 250 µM, pyochelin depletes iron and other metals to inhibit the growth of *A. fumigatus*.^162^ In contrast, at lower concentrations of 62–125 µM, pyochelin stimulates *A. fumigatus* growth and is an iron source for the fungi under iron-limited conditions.^162^ Further, pyochelin can also penetrate *A. fumigatus* cells, and exhibits cytotoxicity by inducing production of ROS.^162^ Similarly, by enhancing ROS production and disrupting cytoplasmic membrane integrity, pyochelin inhibits the growth of *Enterococcus* spp. (MIC: 9.7 µM) and *S. aureus* (MIC: 19.3 µM) *in vitro*.^163^ Production of enterobactin (see 2.2.3 above) by *E. coli* provides protection against pyochelin-induced ROS,^164^ demonstrating the importance of siderophores in defense against microbial antagonism, beyond functioning solely in iron acquisition. Iron-bound pyochelin can also catalyze the formation of hydroxyl radicals that could lead to oxidative damage. As a further mechanism of detoxification, *S. aureus* has been shown to use an methyltransferase called staphylococcal pyochelin methyltransferase to convert pyochelin into pyochelin methyl ester,^165^ which exhibits reduced iron affinity, and may help to facilitate coexistence between *S. aureus* and *P. aeruginosa.*^166^

Pyochelin also induces ROS in both human immune and pulmonary cells, thereby contributing to the virulence of *P. aeruginosa*.^167^ For instance, iron-bound pyochelin catalyzes formation of hydroxyl radicals, damaging pulmonary artery endothelial cells and airway epithelial cells.^147,167^ Intriguingly, iron-bound pyochelin, but not iron-bound pyoverdine, is capable of generating hydroxyl radicals, suggesting pyochelin can directly contribute to oxidative stress and cause host tissue damage.^168^ In *Burkholderia* spp., though pyochelin production alone is not sufficient for growth under iron-limited conditions *in vitro*, production of this compound enhances virulence.^147^ In a chronic pulmonary infection model of rats, the addition of exogenous pyochelin results in increased virulence of *B. cepacia* pyochelin-negative bacterial mutants.^169^ Together, these findings highlight the multifaceted role that pyochelin plays in the CF airway.

#### 2.2.9. Pyoverdine

Pyoverdine (29) is the other of the two major siderophores produced by *P. aeruginosa*.^152,153^ The *pvd* BGC for pyoverdine biosynthesis is conserved across *P. aeruginosa*, but there is variation in the adenylation domains of the NRPSs, which results in a strain-specific composition of the peptide chain. The pyoverdines are mixed catechol–hydroxamate siderophores that contain six to twelve amino acids, a C_4_ or C_5_ α-ketoacid derived from the tricarboxylic acid cycle, and a chromophore derived from 2,3-diamino-6,7-dihydroxyquinoline, which is responsible for fluorescence.^170,171^ Over 100 variants of this siderophore have been described from *Pseudomonas*, and even different strains of the same species produce different variants.^172,173^ We will focus our discussion on the pyoverdine variants produced by *P. aeruginosa* strain PAO1, but the principles are similar for other strains.

The precise order of events involved in peptide chain assembly, chromophore formation, and α-ketoacid incorporation in pyoverdine biosynthesis are unresolved.^174^ We will describe each in turn. The biosynthesis of the peptide chain is initiated by the NRPS PvdL using myristic acid or myristoleic acid as a starter unit followed by typical NRPS extension.^175^ PvdL then extends this starter unit with l-glutamate, d-tyrosine, and l-2,4-diaminobutyrate (l-Dab). The latter two residues form a tetrahydropyrimidine ring that serves as the chromophore precursor. Subsequently, the chain is elongated by the NRPSs PvdI, PvdJ, and PvdD to produce the acylated ferribactin precursor.^176^ The l-Dab residues in this precursor are produced by the l-2,4-diaminobutyrate:2-ketoglutarate 4-aminotransferase PvdH and the two l-N5-formyl-N5-hydroxyornithine residues are produced after hydroxylation and formylation of l-ornithine by the l-ornithine N5-oxygenase PvdA and the formyltransferase PvdF, respectively.^177–179^ The ferrinbactin precursor is then exported from the cytoplasm to the periplasm by the ATP binding cassette (ABC) transporter PvdE,^180^ where the acyl chain is removed by the Ntn hydrolase PvdQ.^181^ Next, the tyrosinase-like PvdP catalyzes oxidative cyclization to form the mature fluorescent chromophore.^182^ PvdO is implicated in a further oxidation step involved in chromophore maturation, but its biochemical activity is not yet established.^183^ The l-Glu residue is transformed to yield various sidechains by an, as of yet, uncharacterized mechanism. Together, these events yield mature pyoverdine (Fig. 15).

**Fig. 15:**
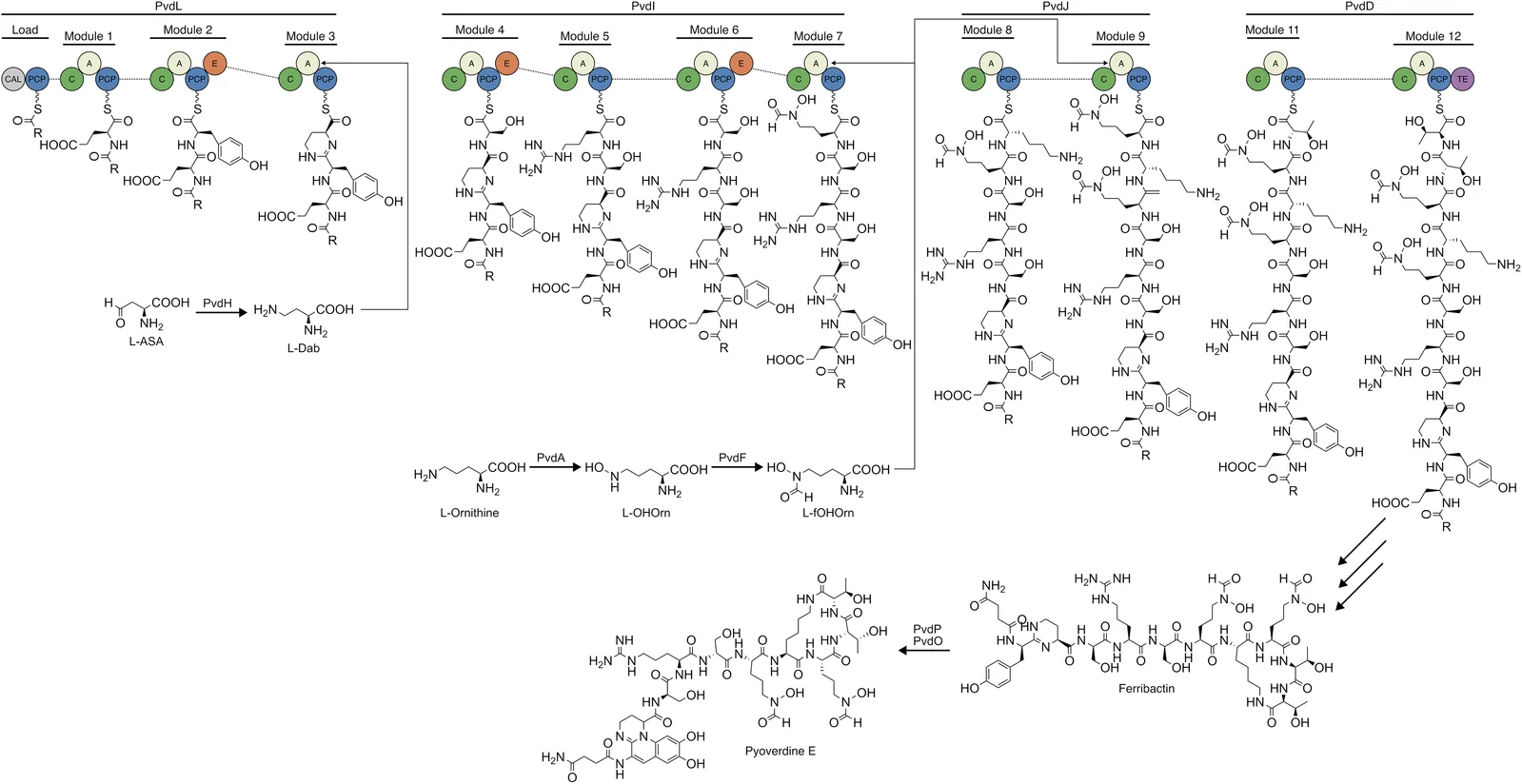
Proposed pathway for pyoverdine biosynthesis. Domain and module organization of the pyoverdine NRPS showing the proposed structure of each PCP-bound intermediate. Proteins and modules are labeled. Dashes indicate modules that are part of the same polypeptide. The tetrahydropyrimidine ring is depicted being installed during assembly line-mediated extension, but it is unclear when this modification is formed. The final product depicted is pyoverdine E, which is produced by *P. aeruginosa* strain PAO1. Different pyoverdine variants are produced by different *Pseudomonas* species and strains. Domain abbreviations: A, adenylation; C, condensation; CAL, co-enzyme A ligase; E, epimerization; PCP, peptidyl-carrier protein.

Pyoverdine is a well-studied molecule with established roles in *P. aeruginosa* growth and physiology.^150,184,185^ Therefore, we will focus on the roles pyoverdine plays in the CF airway, where it facilitates colonization, interactions between *P. aeruginosa* and other members of the microbiota, and virulence.^152,184^ Pyoverdine is critical for colonization of the airways by *P. aeruginosa*, as this pathogen depends on iron acquisition from the host for its survival.^152^ The host sequesters iron using lactoferrin, ferritin, transferrin, hemoglobin, and other heme-containing proteins.^152,186^ Strains of *P. aeruginosa* that produce pyoverdine and pyochelin are capable of removing iron from lactoferrin and transferrin to promote their own growth.^152,187^ Among these siderophores, pyoverdine exhibits a higher affinity for iron (*K*_f_ = 10^24^ M^−1^) compared to pyochelin (*K*_f_ = 10^5^ M^−1^).^188,189^ Under conditions of strong iron-limitation, *P. aeruginosa* tend to upregulate pyoverdine production, while repressing pyochelin production.^189,190^ Accordingly, pyoverdine biosynthesis mutants grows more poorly in the lungs than either wild-type *P. aeruginosa* or pyochelin biosynthesis mutants.^152^

Similarly to pyochelin, pyoverdine mediates interactions between *P. aeruginosa* and *A. fumigatus*.^191^ But unlike pyochelin, pyoverdine is not used as an external ferrochelator by *A. fumigatus*.^162^ Consistent with its function as a siderophore, pyoverdine inhibits *A. fumigatus* growth by depriving it of iron.^191^ Isolates of *P. aeruginosa* from pwCF exhibit a direct correlation between pyoverdine production and antifungal activity.^191^ Cell-free supernatants from *P. aeruginosa* inhibit hyphal growth and branching of *A. fumigatus*, whereas supernatants from a pyoverdine biosynthesis mutant have reduced inhibitory activity.^192^ Under microaerophilic and anaerobic conditions, due to a decrease in the ratio of ferric to ferrous iron, pyoverdine production is reduced and *A. fumigatus* exhibits better survival.^188,191^ During interactions with *Scedosporium* spp., pyoverdine also acts as a growth inhibitor,^86^ but *S. apiopsermum* extract iron from pyoverdine using *N*^α^-methylcoprogen B (see 2.2.5 above) helping the fungus compete against *P. aeruginosa*. Competition for iron between *P. aeruginosa* and *C. albicans* also occurs within mixed biofilms and increases pyoverdine production. This competition may also lead to increased virulence, as iron-regulated proteases, including AprA, PasP, and PrpL, degrade human serum proteins and disrupt tight junctions.^193,194^

Consistent with its activity against fungi, pyoverdine also mediates interactions with other bacteria found in the airways of pwCF. For instance, pyoverdine inhibits the growth of *Burkholderia* spp. and *Pandoraea* spp. isolates from pwCF *in vitro*.^86^ Interactions between *P. aeruginosa* and *Streptococcus* spp. are generally beneficial to the latter, but elevated siderophore production by *P. aeruginosa* can inhibit streptococcal growth.^195^ Furthermore, pyoverdine, along with pyochelin (see 2.2.8 above) and HQNO (see 2.1.6 above), can act as both a growth inhibitor^58^ and activator^196^ of *S. aureus*. Thus, pyoverdine supports *P. aeruginosa* persistence in the iron-limited CF airway and mediates its interactions with other microbes. Beyond facilitating iron acquisition, pyoverdine contributes to the virulence of *P. aeruginosa* through multiple mechanisms (see 2.4.7 below for a separate discussion on virulence).

#### 2.2.10. Staphyloferrins

Staphyloferrin A (30) and B (31) are a pair of hydroxycarboxylate siderophores produced by *S. aureus* and other staphylococci that are synthesized *via* two distinct NRPS-independent (NIS) BGCs, denoted *sfna* and *sbn*, respectively.^197–199^ In contrast to the *sfna* BGC, the *sbn* BGC is limited to a smaller number of staphylococcal species.^197^ The biosynthesis of staphyloferrin A is initiated by SfnaD, which catalyzes the condensation of d-ornithine and citrate. Subsequently, SfnaB condenses this intermediate with a second citrate to yield staphyloferrin A (Fig. 16A).^198^ Production of staphyloferrin B begins with ATP-dependent activation of l-serine by SbnI to generate *O*-phospho-l-serine (OPS).^200^ Then, SbnA condenses l-glutamate and OPS in a PLP-dependent reaction to form *N*-(1-amino-1-carboxy-2-ethyl)-glutamic acid. Subsequently, oxidative hydrolysis of this intermediate by SbnB produces l-2,3-diaminopropionic acid (l-DAP) and α-ketoglutarate, which are two of the precursors for staphyloferrin B.^201^ The NIS synthetases SbnE catalyzes condensation of citrate and l-DAP, after which SbnH decarboxylates an l-Dap-containing intermediate to generate a diaminoethane moiety.^202^ The NIS synthetases SbnF and SbnC then catalyze sequential condensation of an additional l-DAP and α-ketoglutarate to yield staphyloferrin B (Fig. 16B).^202^

**Fig. 16:**
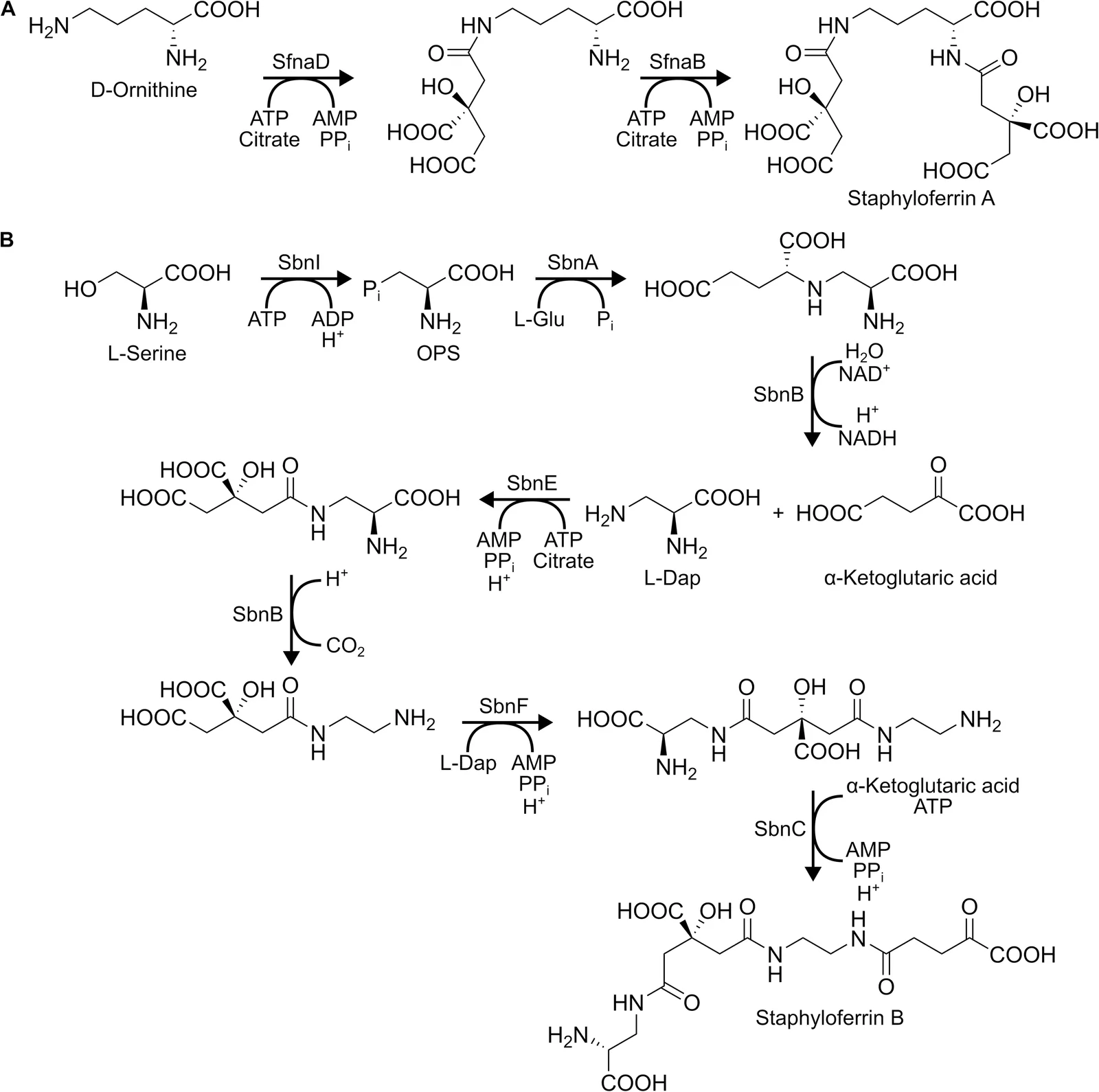
Pathway for biosynthesis of (A) staphyloferrin A and (B) staphyloferrin B.

Intriguingly, both staphyloferrin A and B are citrate-derived, but production of the primary citrate synthase CitZ is downregulated during iron starvation.^203^ However, *sbnG* encodes a novel citrate synthase that enables production of staphyloferrin B, independent of the citric acid cycle.^203^ This suggests that staphyloferrin B is produced under iron limiting conditions, potentially supporting the initial colonization of *S. aureus* in the airways of pwCF.^203^ Indeed, staphyloferrin B biosynthesis genes are upregulated in *S. aureus* from CF sputum.^204^ Furthermore, staphyloferrin B biosynthetic genes are upregulated in clinical isolates of coagulase-negative staphylococci, rather than the non-clinical isolates, suggesting that the compound might play a role in pathogenicity.^205,206^ However, in both murine abscess models and human lung adenocarcinoma cell line models, mutants that are unable to produce the staphyloferrin A importers exhibited growth defects, whereas mutants for the staphyloferrin B importer were indistinguishable from wild-type *S. aureus*.^207^ While this finding indicates that staphyloferrin A import is important during persistence, there also remains the possibility that *S. aureus* encodes other yet-to-be identified transporters of staphyloferrin B.^208^ Overall staphyloferrins are important for the *S. aureus* colonization and virulence.

#### 2.2.11. Staphylopine and pseudopaline

Staphylopine (32) is a broad-spectrum metallophore produced by *S. aureus* that chelates cobalt, copper, iron, nickel, and zinc.^209,210^ The biosynthesis of staphylopine is dependent upon the *cnt* BGC.^210^ First, l-histidine is converted to d-histidine by the histidine racemase CntK.^211^ Subsequently, CntL transfers an aminobutyrate moiety from *S*-adenosylmethionine to d-histidine.^210^ Finally, the opine dehydrogenase CntM performs a NADPH-dependent reductive condensation of pyruvate with the intermediate to yield staphylopine (Fig. 17).^210^ Though staphylopine chelates multiple metals, this natural product functions primarily as a zincophore. Within the host, *S. aureus* uses staphylopine to compete for zinc with the immune effector protein calprotectin. This protein functions as part of a host-mediated defense mechanism against pathogens by binding zinc with a high affinity and contributing to nutritional immunity.^209^ Under the zinc-limiting conditions imposed by the host, staphylopine is induced, which enables *S. aureus* to acquire this metal and grow. Accordingly, staphylopine is capable of facilitating Zn acquisition in murine infection models. Further, expression of *cntF* and *cntM* has been detected in expectorated sputa, suggesting that this compound might play a key role for the growth and virulence of *S. aureus* in the airways of pwCF.

**Fig. 17:**
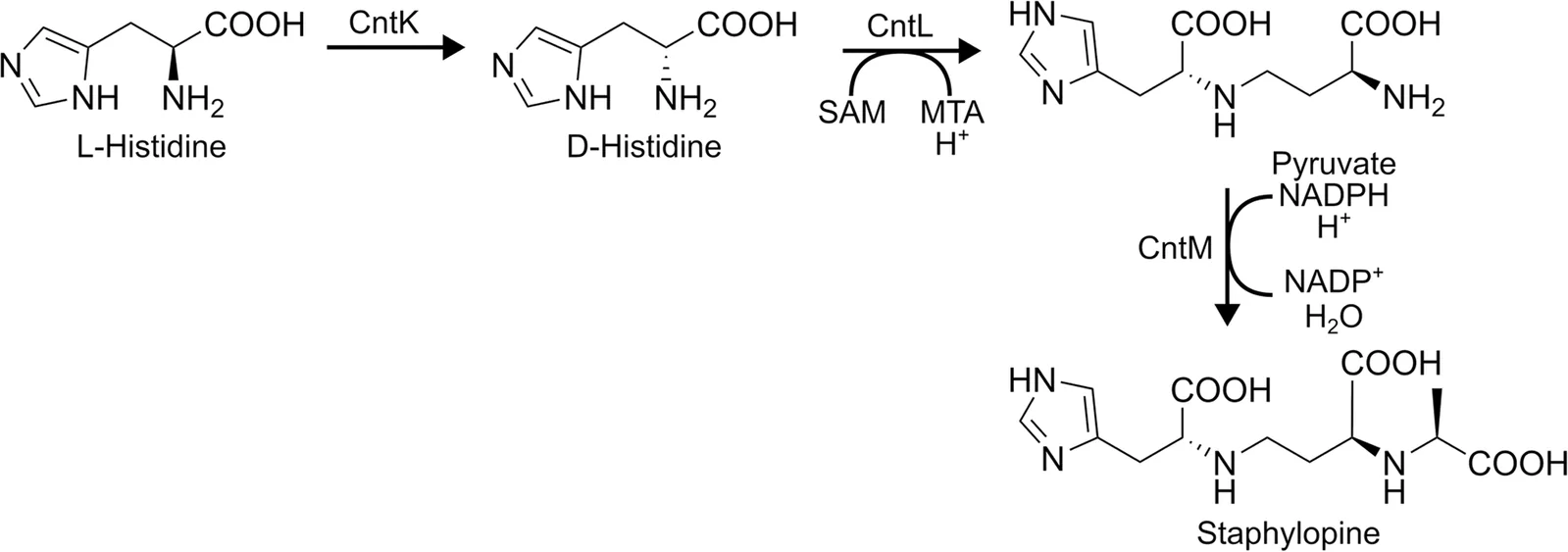
Pathway for biosynthesis of staphylopine.

A homologous BGC for biosynthesis of natural products similar to staphylopine have been reported in other bacterial pathogens.^210,212^ In *P. aeruginosa*, this compound is called pseudopaline (33), which is an epimer of staphylopine that contains an l-histidine residue, instead of d-histidine, condensed with an aminobutyrate moiety.^212^ A *cntL* mutant of *P. aeruginosa* exhibits defects in biofilm formation and increased sensitivity to tobramycin. In a murine pulmonary infection model, animals infected with a pseudopaline biosynthesis mutant had lower bacterial burden and mortality, suggesting that this natural product plays a vital role in *P. aeruginosa* virulence and persistence.^213^

#### 2.2.12. Triacetylfusarinine C

Triacetylfusarinine C (also called triacetylfusigen) (34) is a hydroxamate siderophore produced by *A. fumigatus* and other fungi. The biosynthesis of this siderophore is dependent on the *sid* BGC and begins with hydroxylation of l-ornithine by the N5-monooxygenase SidA.^214^ Next, the transacetylase SidF acetylates N5–OH-l-ornithine using anhydromevalonyl-CoA as a donor.^215^ The NRPS-like SidD catalyzes condensation of three molecules of N5-anhydromevalonyl-N5-hydroxyornithine to generate the cyclic compound fusarinine C.^216^ Finally, the acetyltransferase SidG is thought to acetylate the three primary amine groups of fusarinine C to yield triacetylfusarinine C (Fig. 18).^217^

**Fig. 18:**
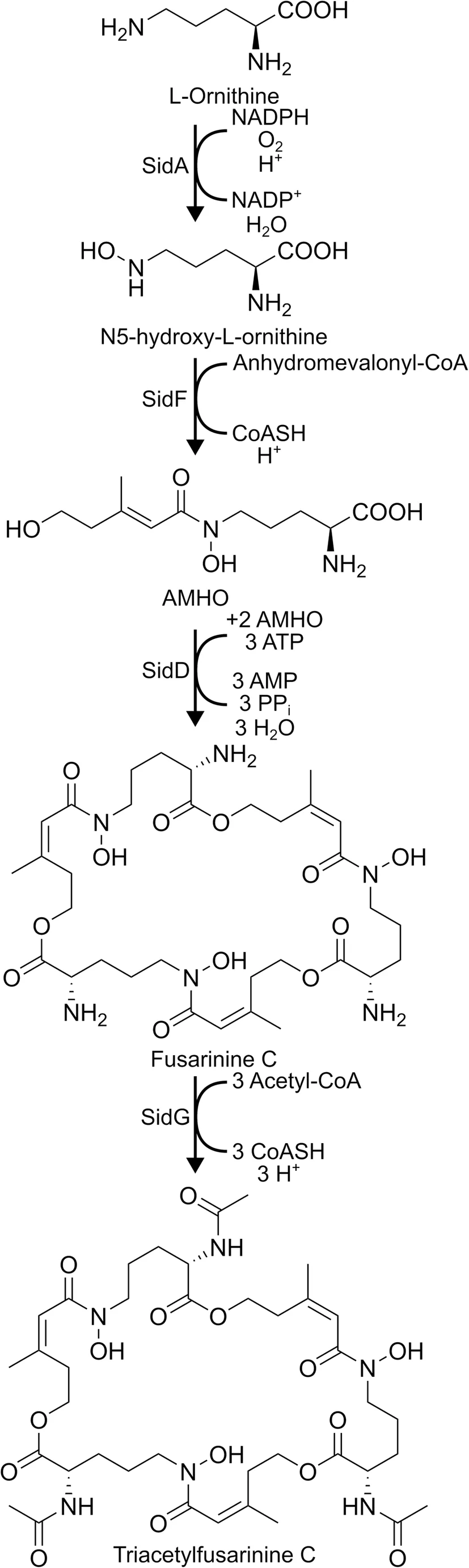
Pathway for biosynthesis of triacetylfusarinine C.

Though a specific role for triacetylfusarinine C in the CF airway has not been documented, this siderophore is crucial for pulmonary colonization by *A. fumigatus*. For instance, *mirB* deletion mutants of *A. fumigatus* are unable to import iron-bound triacetylfusarinine C and exhibit attenuated virulence in a murine model of aspergillosis. In contrast, *mirD* mutants, which are unable to import fusarinine C had no such defect.^218^ These *mirB* mutants are also unable grow on blood, suggesting that triacetylfusarinine C is critical for acquiring iron from transferrin.^218,219^ Notably, this siderophore accumulates in the lungs of rats infected with *A. fumigatus* and can be detected within the urine of people with pulmonary aspergillosis,^220,221^ indicating their potential to reach high concentrations within hosts. Further, triacetylfusarinine C may be involved in interactions with *P. aeruginosa* and other members of the CF microbiota. The phenazine 1-HP produced by *P. aeruginosa* (see 2.1.10 above) chelates iron and induces iron starvation in *A. fumigatus*. In response to 1-HP, *A. fumigatus* produces triacetylfusarinine C.^104^ In contrast, in mixed species biofilms, *P. aeruginosa* reduces the production of several *A. fumigatus* natural products, including triacetylfusarinine C.^222^ Thus, this compound can play a variety of roles in resource competition in the airways of pwCF. As siderophores play an important role in the virulence and persistence of several bacterial pathogens in the CF airway, triacetylfusarinine C may similarly contribute to the virulence of *A. fumigatus*.

### 2.3. Signals

Members of the CF airway microbiota produce natural products that function in intraspecies quorum sensing and interspecies communication (Fig. 19). Here, we will focus on the known signaling molecules that are produced by members of the CF microbiota or have been detected in the CF airway. For well-established signaling molecules, we describe their biosynthesis, mechanisms of signaling, and relevant downstream targets.

**Fig. 19:**
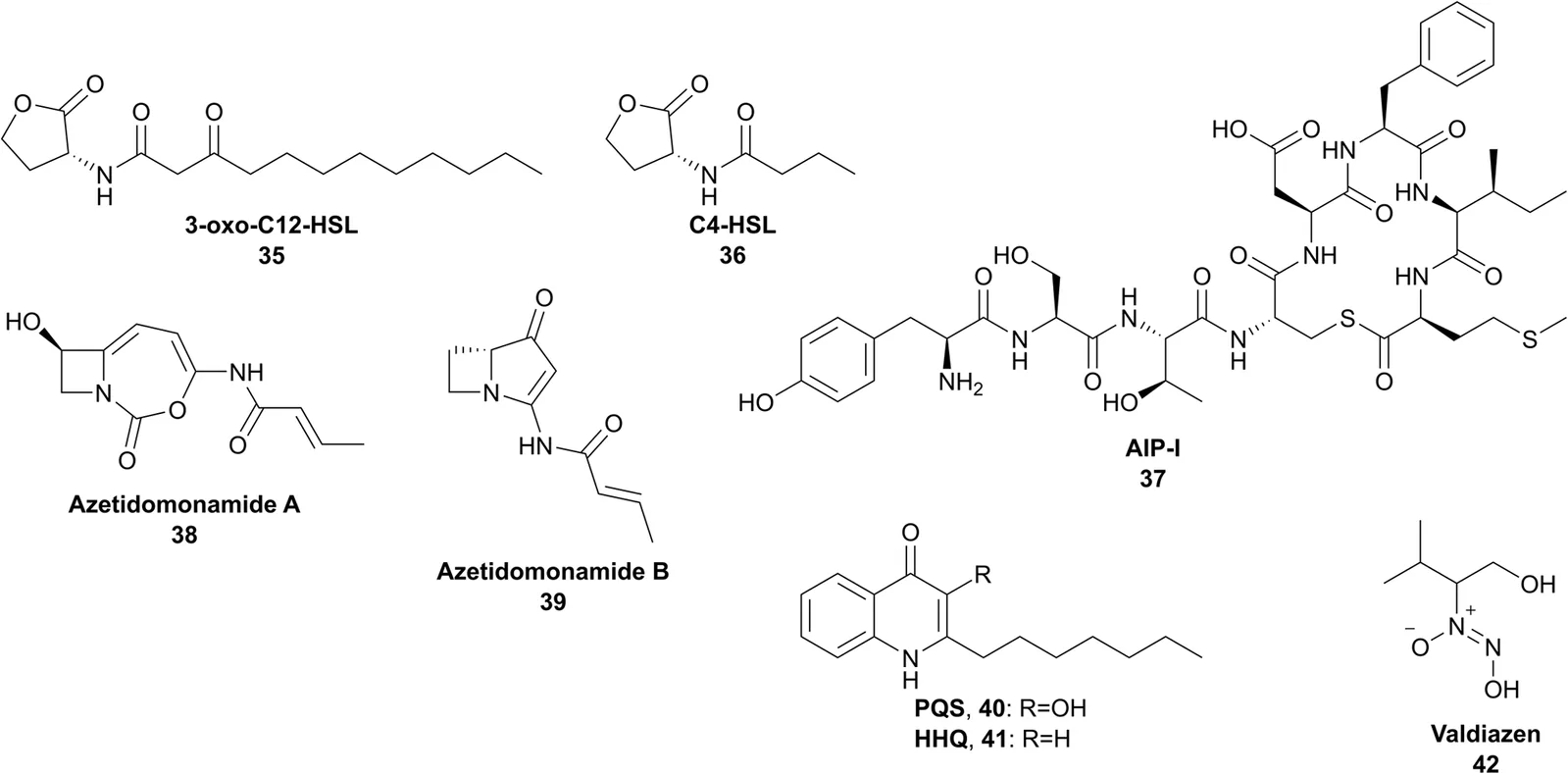
Structures of signaling molecules produced by CF pathogens. Structure 37 represents AIP-1 from *S. aureus*, but other AIP variants are produced by *Staphylococcus* spp.

#### 2.3.1. *N*-Acylhomoserine lactones

Quorum sensing is a phenomenon in which bacteria regulate gene expression based on cell density and perform various population-level activities (recently reviewed).^223,224^ In quorum sensing, bacteria produce and release diffusible signal molecules that increase in extracellular concentration concomitantly with increases in cell density. When a minimum threshold stimulatory concentration of the molecule is reached, the signals reenter cells, where they alter gene expression.^225^ The paradigmatic example of quorum sensing molecules are the *N*-acylhomoserine lactones (AHLs).^226^ The two AHLs produced by *P. aeruginosa* are *N*-3-oxododecanoyl homoserine lactone (3-oxo-C12-HSL) (35) and *N*-butyryl homoserine lactone (C4-HSL) (36), which are controlled by the LasI/LasR and RhlI/RhlR systems, respectively. The biosynthesis of 3-oxo-C12-HSL begins with the AHL synthase LasI, which uses the alpha-amino group of SAM to attack the thioester bond of an acyl carrier protein loaded with a 3-oxododecanoic acid derived from fatty acid biosynthesis. Subsequently, this acyl-SAM intermediate undergoes intramolecular cyclization, forming the homoserine lactone ring and releasing methylthioadenosine as a byproduct (Fig. 20A). The biosynthesis of C4-HSL is similar, but RhlI uses SAM to attack an acyl carrier protein loaded with butanoic acid (Fig. 20B). Due to their amphipathic nature, 3-oxo-C12-HSL and C4-HSL are able to pass through the membrane, where they interact with their cellular targets, LasR, and RhlR, respectively, and elicit changes in gene expression.^227^ Importantly, the presence of AHLs have been detected in sputum from pwCF colonized by *P. aeruginosa* and *B. cepacia* using both mass spectrometry and engineered *lux* biosensors.^228–230^ As, the LasI/LasR and RhlI/RhlR systems are well-characterized and recently reviewed,^231,232^ we will provide a broad overview of the downstream targets of these systems and recent literature on the role of AHLs in mediating interspecies interactions in the CF airway and regulating virulence.

**Fig. 20:**
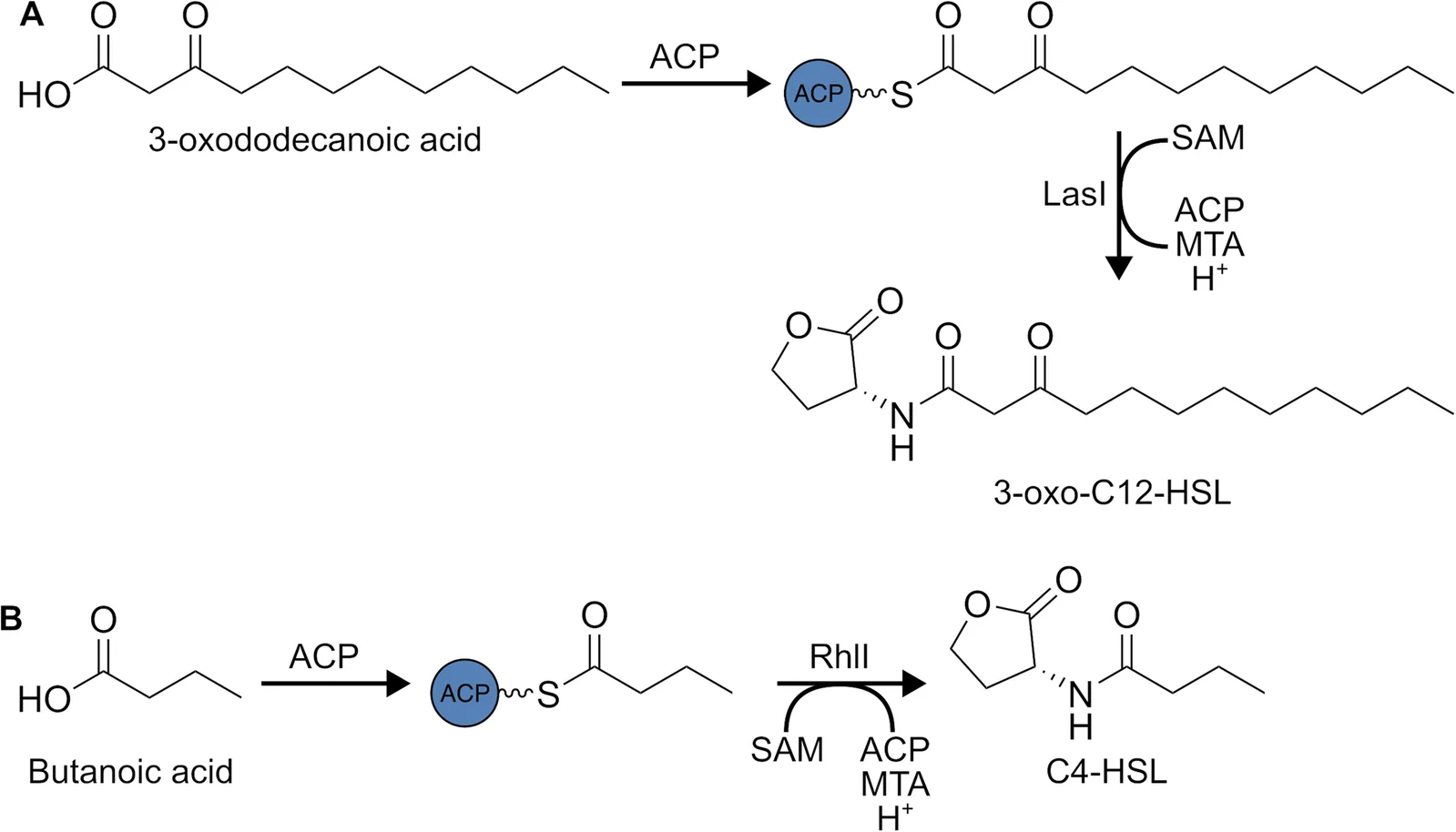
Pathway for biosynthesis of (A) 3-oxo-C12-HSL and (B) C4-HSL.

In addition to acting as signaling molecules, AHLs mediate interspecies interaction through antibiotic activity. For example, cell-free supernatants containing AHLs inhibit growth and hyphal formation of *C. albicans* when added to growing fungal cultures.^233^ At 200 µM, 3-oxo-C12-HSL is capable of suppressing filamentation of *C. albicans*.^234^ AHLs from *P. aeruginosa* also inhibit growth and biofilm formation of *A. fumigatus*.^235^ The LasI/LasR quorum sensing is important for this inhibition, as growth of *A. fumigatus* exhibited increased growth during coculture with *P. aeruginosa lasI* and *lasR* mutants compared to coculture with the wild-type strain.^235^ 3-Oxo-C12-HSL also activates production of the *Pseudomonas* quinolone signal, another quorum sensing natural product (see 2.3.4 below). Physiologically relevant concentrations of 3-oxo-C12-HSL can modulate *S. aureus* behavior.^236^ In mixed species biofilms with *S. aureus*, *P. aeruginosa* can produce more than 600 µM of 3-oxo-C12-HSL.^237^ This AHL inhibits growth (75 µM) and production of the virulence factors exotoxin and fibronectin-binding protein production (10 µM) in *S. aureus*.^236^

3-Oxo-C12-HSL and C4-HSL are required for colonization of the lungs of adult mice by *P. aeruginosa*.^238^ Further, several virulence factors are also regulated by AHL-mediated quorum sensing. For instance, the virulence factors elastase and rhamnolipids (see 2.4.8 below) are regulated by LasR and RhlR, respectively.^239,240^ 3-Oxo-C12-HSL and C4-HSL activate expression of genes for production of hydrogen cyanide, alkaline protease-like virulence factors, and phenazines (see 3.1.10 above).^241,242^ Apart from virulence, quorum sensing in *P. aeruginosa* is required for twitching motility and biofilm formation.^238,243,244^ 3-oxo-C12-HSL can impact leukocytes and induce production of IL-8 in respiratory epithelial cells.^236,245^ In pwCF, interleukin 8 (IL-8) induction and neutrophil infiltration are part of the inflammatory milieu associated with airway disease.^238,246^ 3-oxo-C12-HSL can also activate immune responses in lymphocytes^247^ and can act as an inducer of inflammation *in vivo*.^238,243,244^ Furthermore, quorum sensing regulates the expression of several antibiotic resistance genes and may contribute to the development of multidrug resistance.^248^

Thus, AHLs play a multifaceted role in the survival and virulence of *P. aeruginosa* in the CF airway. Beyond *P. aeruginosa*, other bacteria that colonize the CF airway have either been experimentally confirmed to produce AHLs, or possess AHL BGCs, though the functions of these cognate molecules remain relatively unexplored.

#### 2.3.2. Autoinducing peptides

Autoinducing peptides (AIPs) (37) are cyclic peptides produced by *S. aureus* and other staphylococci that act as quorum sensing signals.^249^ The biosynthesis of AIPs follows the standard for RiPP biosynthesis. The precursor peptide AgrD is synthesized by the ribosome and contains an N-terminal leader, the core peptide, and C-terminal follower sequences.^250,251^ The N-terminal leader guides the precursor peptide to the membrane-associated protease AgrB, which catalyzes intramolecular thiolactone formation between the side chain of a conserved l-cysteine residue and the α-carboxyl group of a variant C-terminal residue. This reaction releases the C-terminal follower sequence.^251,252^ Following cyclization, the thiolactone-containing intermediate is processed by another membrane-associated peptidase MroQ, which removes the N-terminal leader sequence to yield AIP, which is likely exported *via* AgrB.^253^ Upon accumulation in the extracellular environment, the thiolactone moiety of AIP binds to the two-component signaling system histidine kinase AgrC, triggering phosphorylation of the response regulator AgrA.^251,254^ Phosphorylated AgrA then activates expression of the P2 promoter RNAII and the P3 promoter RNAIII^254^ that drives downstream virulence gene expression including leukotoxins, phenol soluble modulins, α-toxin, and δ-toxin.^249,255^

#### 2.3.3. Azetidomonamide A and B

Although *P. aeruginosa* is among the most well-studied bacterial species, the release of the *P. aeruginosa* PAO1 reference genome in the year 2000 revealed the presence of six NRPS BGCs, yet only pyochelin (see 2.2.8 above), pyoverdine (see 2.2.9 above), and l-2-amino-4-methoxy-*trans*-3-butenoic acid were known to be produced by this pathogen.^256^ Transcriptomic analysis of a spontaneous colistin-resistant *P. aeruginosa* isolate revealed increased expression of one of these uncharacterized BGCs, which is highly conserved in *P. aeruginosa* genomes, but the cognate natural product was unknown.^257,258^ Hong *et al.* identified the azetidine-containing compounds azetidomonamide A (38) and B (39) as the major products of this BGC, which they subsequently named *aze*. The biosynthesis of the azetidomonamides is still not fully understood, but it is thought to proceed from intramolecular cyclization of *S*-adenosyl methionine (SAM) by the SAM-dependent lyase AzeJ to form a rare azetidine-2-carboxylic acid, which is subsequently adenylated and incorporated into the growing peptide chain by the AzeB NRPS.^257^ Next, on-line cyclization is predicted to occur through nucleophilic attack of a serine-derived dehydroalanine residue by the thioesterase domain and enabled by electron delocalization from the enamine nitrogen contributed by the crotonyl starter unit.^257^ The monooxygenase AzeC then catalyzes a Baeyer-Villiger oxidation, leading to ring expansion. Subsequent ring tailoring then yields azetidomonamide A, while an alternative AzeC-dependent oxidative ring expansion followed by decarboxylation and dehydration yields azetidomonamide B (Fig. 21).^257^

**Fig. 21:**
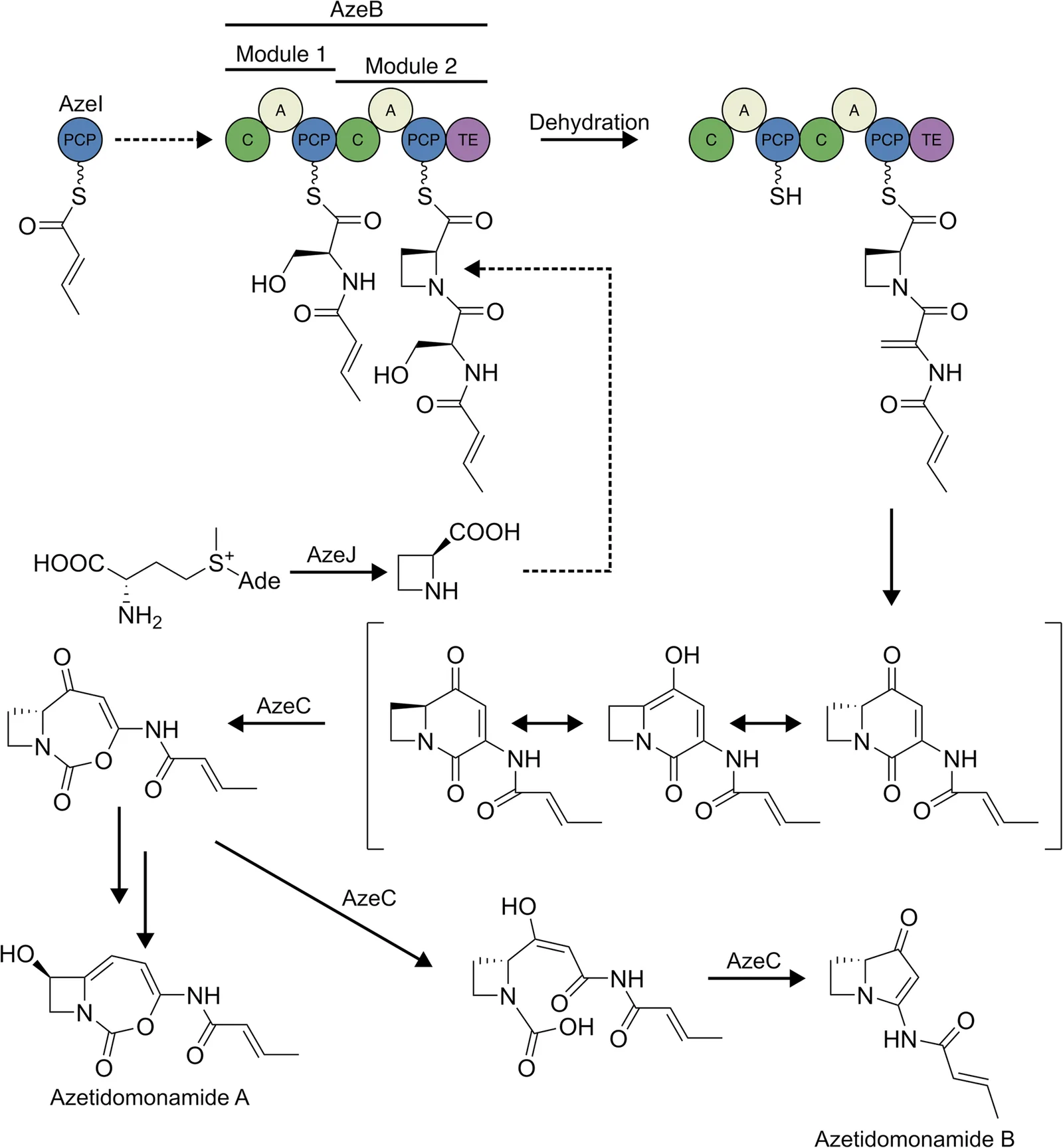
Proposed pathway for azetidomonamide A and B biosynthesis. Domain and module organization of the azetidomonamide NRPS showing the proposed structure of each PCP-bound intermediate. Proteins and modules are labeled. The proposed mechanism for post-NRPS tailoring reactions are shown below the assembly line. Short-lived intermediates are shown in brackets. Domain abbreviations: A, adenylation; C, condensation; PCP, peptidyl-carrier protein. Based on a figure from ref. 257 with permission from John Wiley and Sons.

Purified azetidomonamide A and B each lack antibiotic activity against *E. coli*, *M. luteus*, and *S. aureus*. However, an *azeB* mutant exhibited a growth-delay phenotype *in vitro* and reduced virulence in a *G. mellonella* model.^257^ Later, using an engineered *P. aeruginosa* PAO1 strain with titratable *aze* expression, Ernst *et al.* observed that expression of this BGC was coregulated with the production of the reddish-brown pigment pyorubin.^259^ This phenotype prompted further investigation into other quorum sensing-regulated and virulence behaviors in *P. aeruginosa*. Azetidomonamide A increased biofilm mass, pigment production, and alkyl quinolone (see 2.3.4 below) levels, while decreasing production of pyoverdine.^259^ Deletion of *lasI* or *rhlI* prevents production of azetidomonamides, establishing a link between these compounds and traditional AHL-mediated quorum sensing (see 2.3.1 above).^259^ Further, overexpression of *aze* led to production of eight additional natural products, whose identities are unknown.^259^ The specific pathways downstream of the azetidomonamides and their role in virulence in the CF airway remain unknown.

Intriguingly, the structurally related natural product clipibicyclene was recently shown to irreversibly inhibit the bacterial ClpP protease, *via* covalent conjugation to an active site serine residue.^260^ However, whether azetidomonamide A similarly targets ClpP or related cellular targets, and whether such activity contributes to *P. aeruginosa* virulence in CF airway remains to be determined.

#### 2.3.4. *Pseudomonas* quinolone signal

*Pseudomonas* quinolone signal (PQS, also called 2-heptyl-3-hydroxy-4-quinolone) (40) is a signaling molecule produced by *P. aeruginosa* that functions with AHLs as part of the quorum sensing cascade (see 2.3.1 above).^261^ The LasI/LasR system controls PQS production by activating expression of *pqsA* and the transcriptional activator encoding gene *pqsR*,^240^ whereas RhlI/RhlR system negatively regulates PQS production by repressing *pqsR*.^261^ The *pqs* BGC that encodes the production of PQS consists of the *pqsABCDE* operon and *pqsH*.^262,263^ This BGC is also responsible for the production of more than fifty additional 2-alkyl-4-quinolones (AQs), in addition to PQS and its biosynthetic precursors.^264^ For instance, 2,4-dihydroxyquinoline (DHQ) is produced *via* a short-lived intermediate synthesized by PqsD,^265^ while PqsL produces alkyl quinolone *N*-oxides, such as HQNO (see 2.1.6 above) from PQS precursors (Fig. 22).^266^ The biosynthesis of PQS first proceeds with activation of anthranilate by addition of coenzyme A by PqsA.^267,268^ PqsD next adds malonyl-CoA to form the intermediate 2-aminobenzoylacetate-CoA (2-ABA-CoA).^269^ It was previously thought that PqsE then hydrolyzes 2-ABA-CoA to intermediate 2-aminobenzoylacetate (2-ABA),^268,270^ but *pqsE* is not required for PQS synthesis.^271,272^ Subsequently, PqsBC catalyzes condensation of octanoyl-CoA with 2-ABA to form 2-heptyl-4-quinolone (HHQ) (41). It is important to note here that PqsBC can also utilize other fatty acyl-CoA substrates, and is at least partially responsible for the structural diversity of AQs produced by *P. aeruginosa*.^268^ Finally, PqsH hydroxylates HHQ to yield PQS (Fig. 22).^273^

**Fig. 22:**
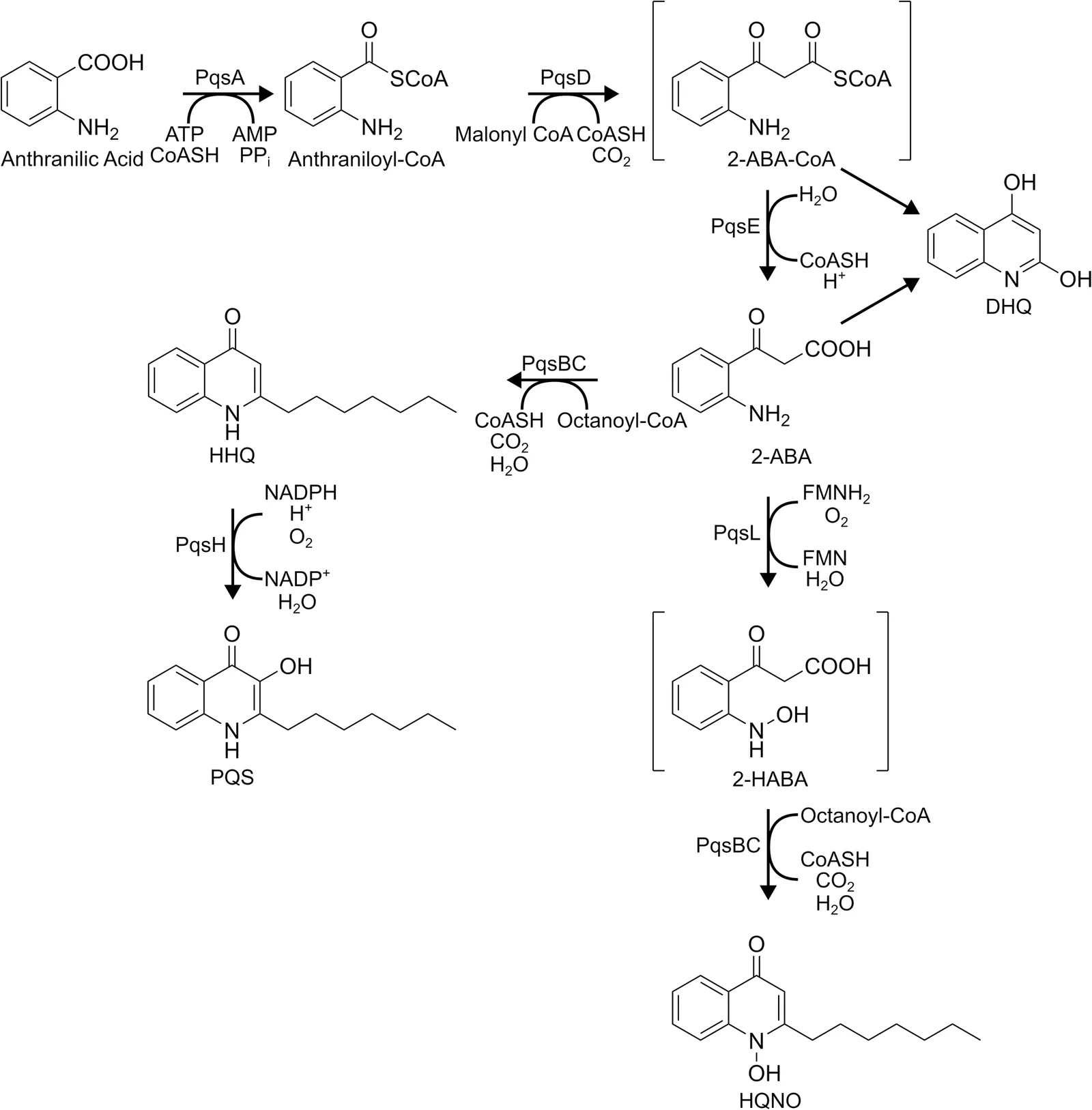
Pathway for biosynthesis of PQS and derivatives. Short-lived intermediates are shown in brackets.

PQS is produced by *P. aeruginosa* in the airways of pwCF and has been detected in sputum, bronchoalveolar lavage fluid, and end-stage lungs removed during transplant in pwCF.^274^ AQs are also hypothesized to contribute to the pathogenicity and dominance of *P. aeruginosa* in the airways of pwCF.^275^ Consistent with this hypothesis, PQS production is elevated in *P. aeruginosa* isolates from asymptomatic children with CF, suggesting that this signaling molecule may be critical during early stages of infection.^276^ The signaling activity of PQS depends on its ability to dissolve in aqueous solution and diffuse freely.^261^ PQS is a lipophilic molecule with low aqueous solubility of ∼5 µM.^262^ The biosurfactant rhamnolipids (see 2.4.8 below) increase the solubility of PQS in aqueous solution,^261^ though the relationship between the two is complex. RhlR inhibits the *pqs* BGC, while PqsE positively regulates rhamnolipid production. PQS thus controls *rhl* quorum sensing and rhamnolipid output, thereby ensuring its own solubility.^261^ Once solubilized, PQS acts as a signaling molecule for several virulence genes, and can act as a virulence factor in its own right (see 2.4.5 below). For instance, PQS and HHQ are required for phenazine production (see 2.1.10 above).^109,275^ PQS also induces production of pyocyanin (see 2.1.10 above) and activates RhlR through PqsE.^277–279^ HHQ, can also act as a signaling molecule, though it primarily functions as a precursor that is converted to PQS.^264^ Both HHQ and PQS can function as ligands of Multiple virulence factor regulator (MvfR) or PqsR and activate expression of the PQS and phenazine biosynthetic genes.^280–282^

Beyond its role in quorum sensing, PQS can mediate interspecies interactions among members of the CF airway microbiota. For instance, PQS chelates iron, and can induce production of siderophores, such as pyoverdine and pyochelin.^264,277^ However PQS does not act like a siderophore itself, as it cannot deliver iron into the bacterial cell. This iron chelation impacts the growth of other bacteria by depriving them of iron.^262,283^ PQS further reduces *B. cenocepacia* virulence by targeting the regulator ShvR, which regulates the quorum sensing signals *cis*-2-dodecenoic acid and AHLs in *B. cenocepacia*.^284,285^ HHQ (10 µM) inhibits biofilm formation in both *B. subtilis* and *C. albicans*,^286^ and growth and motility of several *Vibrio* spp. (MIC: 10 µM) *in vitro*. PQS can also inhibit the respiratory chain of other bacteria, such as *E. coli*. Furthermore, the swarming motility of Gram-negative bacteria is reduced in presence of PQS.^286^ Several microbes have evolved mechanisms to inactivate PQS. For instance, *C. albicans* produces farnesol to decreases the production of PQS by inhibiting the PqsR-mediated transcriptional activation of *pqsA*.^287,288^ Similarly, *A. xylosoxidans* can inactivate PQS by converting it to the inactive compound 2-heptyl-2-hydroxy-1,2-dihydroquinoline-3,4-dione.^289^ Thus, PQS plays variable roles that impact the growth and persistence of *P. aeruginosa* in the airways of pwCF.

#### 2.3.5. Valdiazen

Valdiazen (42) is a signaling molecule that was discovered in *B. cenocepacia* and activates expression of the *ham* BGC to promote its own biosynthesis and the biosynthesis of (−)-fragin (Fig. 11) (see 2.2.4 above).^137^ Valdiazen regulates expression of over 100 genes, including metal transporting ATPases, an extracytoplasmic sigma factor that regulates the *orn* BGC, and pyochelin (see 2.2.8) biosynthesis.^137,290^ As valdiazen is diffusible, it can induce gene expression in adjacent cells, and because it promotes its own biosynthesis, it functions as a novel quorum sensing molecule. Like other diazeniumdiolate-containing molecules, valdiazen has high affinity for ferric iron, but its quorum sensing activity is not dependent on iron chelation.^290^ In addition to *Burkholderia* spp., valdiazen is produced by other bacteria that can colonize the airways in pwCF, including *Pseudomonas* spp. and *Pandorea* spp.^137^ Recently, Crawford *et al.* demonstrated valdiazen as the autoinducer of *Pseudomonas virulence* factor in insect and plant pathogenic strains of *Pseudomonas* spp.^291^ This finding suggests that valdiazen could contribute to expression of virulence factors within the CF airway, but this is yet to be demonstrated.

### 2.4. Toxins and other potential virulence factors

Several natural products produced by *A. fumigatus*, *Burkholderia* spp., and *P. aeruginosa*, *S. aureus* function either as toxins or virulence factors and may contribute to pathogenesis in the CF airway (Fig. 23). For virulence factors that are already established in the CF airway, we describe their activities, their impact on the host, and their relevant half maximal inhibitory concentration (IC_50_) values, when known. In addition, several natural products that act as virulence factors in other contexts have been detected directly in sputa, produced by CF strains, or identified through the presence of their BGCs in CF isolates. In these cases, we speculate on the role of these natural products in the CF airway based on what is known about their activity in other settings.

**Fig. 23:**
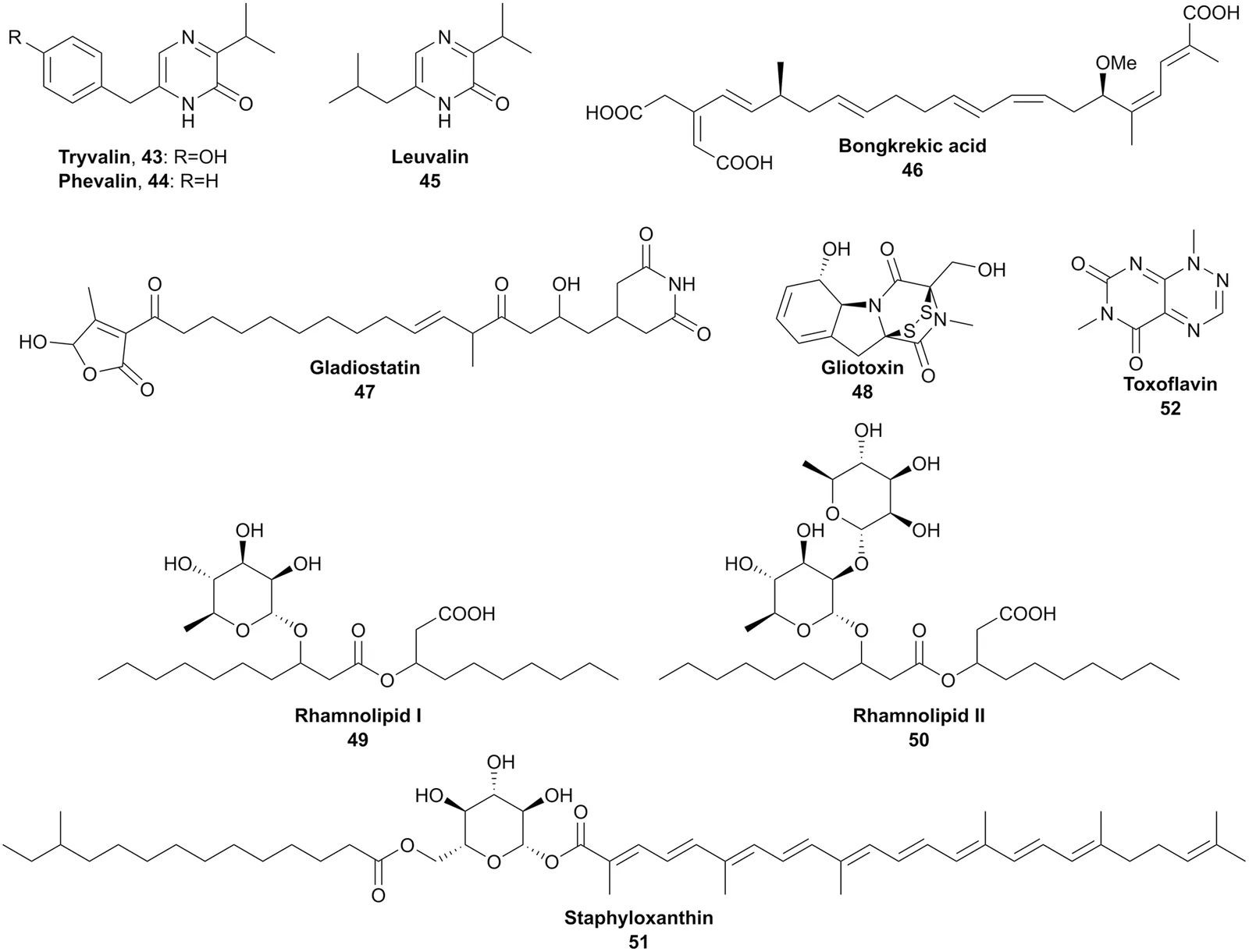
Structures of toxins and virulence factors produced by CF pathogens.

#### 2.4.1. Aureusimines

All sequenced genomes of *S. aureus* and some other staphylococci encode for the *ausBC* BGC. This NRPS BGC is responsible for the production of three cyclic dipeptides formed through the condensation of l-tyrosine or l-phenylalanine, and less frequently, l-leucine, with l-valine to form tryvalin (also called aureusimine A) (43), phevalin (also called aureusimine B) (44), and leuvalin (45), respectively (Fig. 24). Together, these monoketopiperazines natural products are referred to as the aureusimines.^292^ The availability of external amino acids influences the relative abundances of each of the aureusimines produced by *S. aureus*.^293^ Under *in vitro* culture conditions that mimic the nutrient availability in nasal secretions, phevalin is the predominant aureusimine, followed by tryvalin and leuvalin, even though the corresponding adenylation domain of the NRPS exhibits high specificity for l-tyrosine.^293^ When these precursors are unavailable, *S. aureus* performs *de novo* biosynthesis and directs these newly synthesized amino acids specifically toward aureusimine biosynthesis, instead of protein translation.^293^ This finding suggests that aureusimines may have essential biological functions in mediating interactions with the host. Indeed, purified phevalin has been shown to strongly inhibit the protease calpain (IC_50_: 1.3 µM) and to alter gene expression in human keratinocytes.^294,295^ Furthermore, *S. aureus* mutants unable to produce aureusimines exhibit reduced intracellular toxicity toward HeLa cells.^296^ However, the impact of these compounds in the CF airway currently remain unknown.

**Fig. 24:**
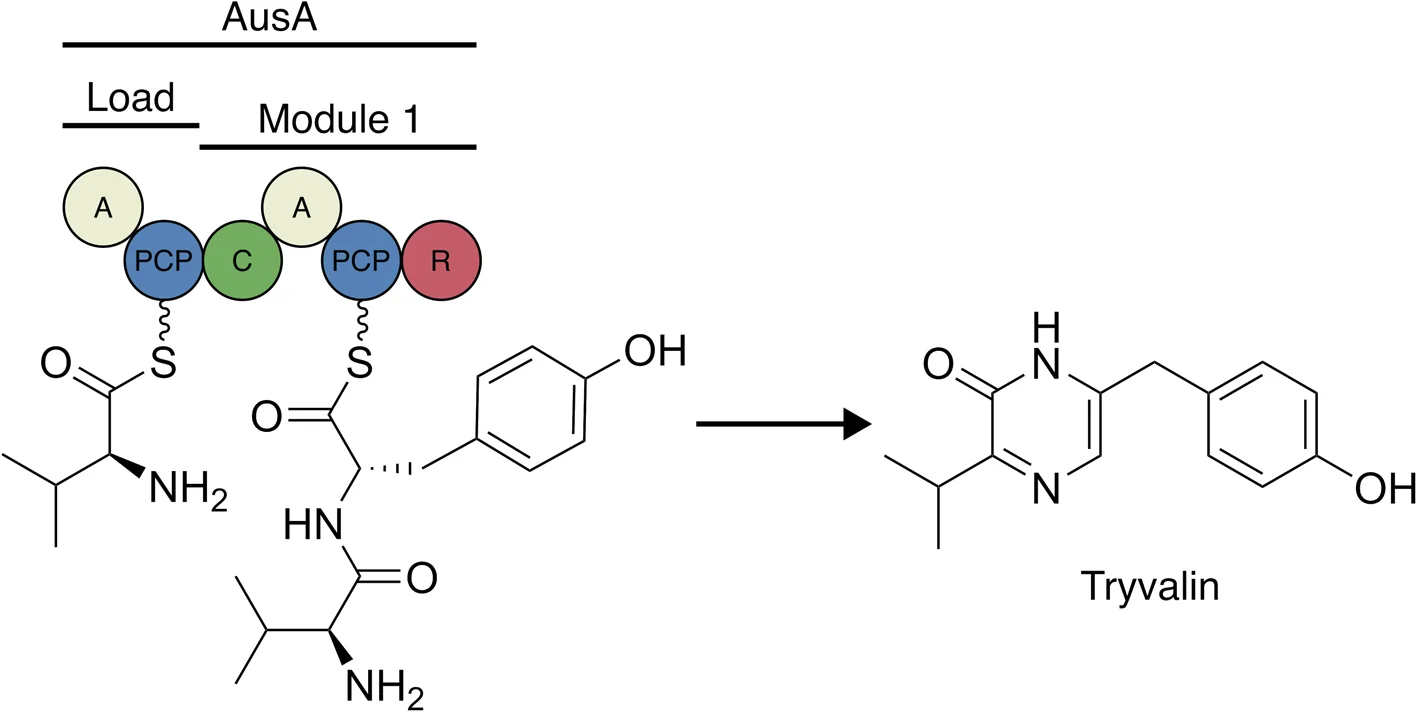
Pathway for tryvalin biosynthesis. Domain and module organization of the aureusimine NRPS showing the proposed structure of each PCP-bound intermediate. Proteins and modules are labeled. Tryvalin is shown as a representative aureusimine. Domain abbreviations: A, adenylation; C, condensation; PCP, peptidyl-carrier protein; R, reductase.

#### 2.4.2. Bongkrekic acid

Bongkrekic acid (46) is a respiratory toxin commonly associated with fermented coconut or corn contaminated by *B. gladioli* pathovar *cocovenenans* (recently reviewed).^297^ This toxin is a conformation-specific competitive inhibitor of the mitochondrial ADP/ATP carrier, that prevents ATP release from the mitochondrial matrix.^298^

Bongkrekic acid is synthesized by a modular *trans*-acyltransferase (*trans*-AT) PKS encoded by the *bon* BGC. During assembly of the PK backbone, the BonF–BonI β-branching enzyme cassette installs an *endo*-β-methyl branch and a carboxymethyl β-branch near the beginning and end of the PK, respectively.^299^ Later during assembly, the *O*-methyltransferase BonM installs a methionine-derived methyl group. Finally, the cytochrome P450 monooxygenase BonL performs a six-electron oxidation of a terminal methyl to yield the final tricarboxylic acid product (Fig. 25).^300^ A survey of sequenced *B. gladioli* CF isolates found the *bon* BGC is present within 25 genomes and further PCR confirmed its presence within 13 of 122 additional *B. gladioli* CF isolates tested, suggesting that this toxin is present among human isolates.^301^ Furthermore, Jones *et al.* demonstrated bongkrekic acid production from 12 *B. gladioli* isolates cultured in synthetic cystic fibrosis sputum medium.^301^ Together, these data indicate that bongkrekic acid has the potential to contribute to the virulence of *B. gladioli* in CF. In addition to acting as a respiratory toxin against human cells, bongkrekic acid exhibits broad-spectrum antifungal activity, including against *Aspergillus* spp.,^300,302^ suggesting that this compound could influence the composition of the CF airway mycobiome.

**Fig. 25:**
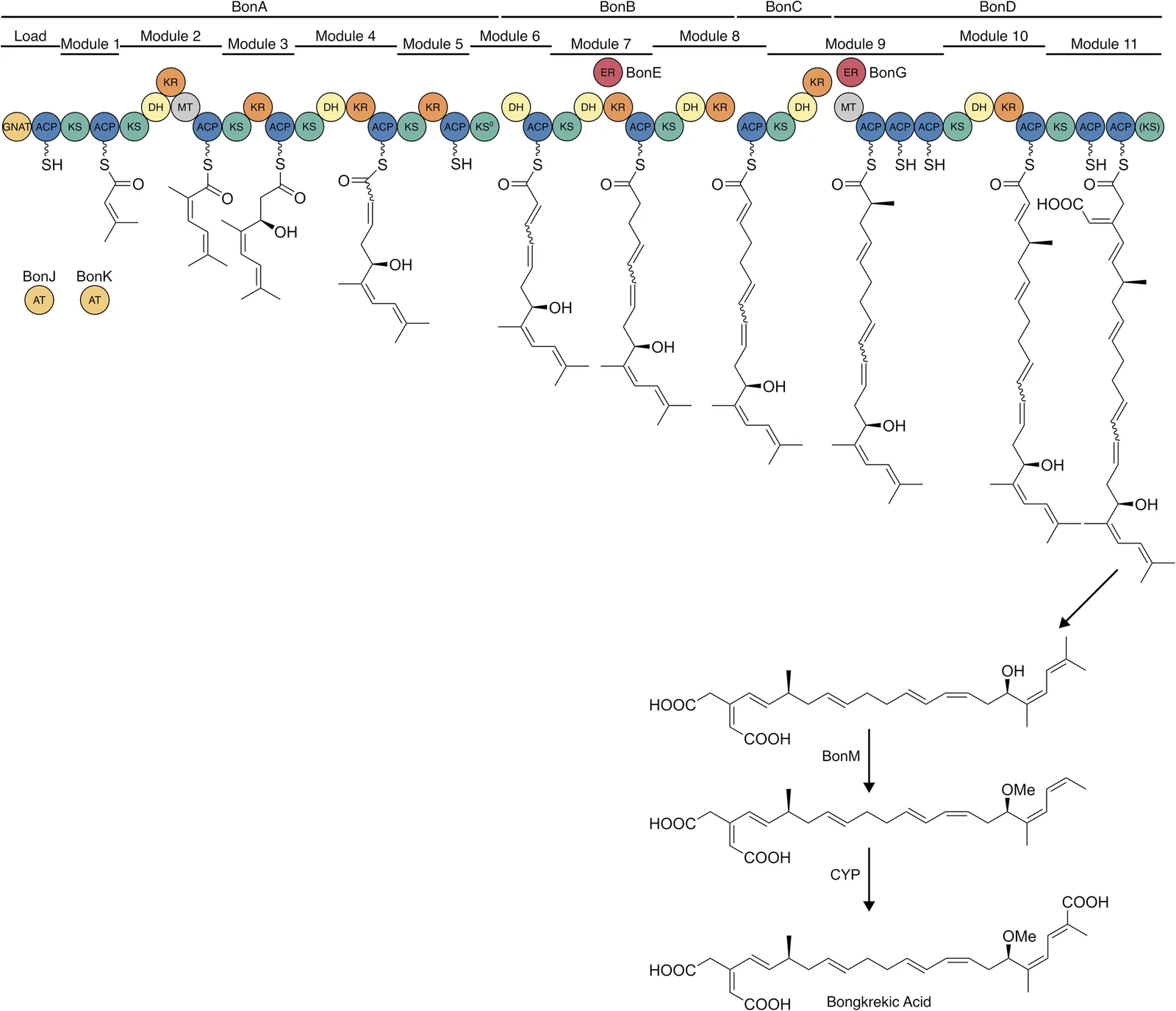
Proposed pathway for bongkrekic acid biosynthesis, Domain and module organization of the bongkrekic acid *trans*-AT PKS showing the proposed structure of each ACP-bound intermediate. Proteins and modules are labeled. The proposed mechanism for post-PKS tailoring reactions are shown below the assembly line. Domain abbreviations: ACP, acyl carrier protein; AT, acyltransferase; DH, dehydratase; ER, enoyl reductase; GNAT, GCN5-related *N*-acetyltransferase-like domain; KR, ketoreductase; KS, ketosynthase; MT, methyltransferase. Based on a figure from ref. 300 with permission from Elsevier.

Intriguingly, coculture with the mucoralean fungus *Rhizopus microspores* increases bongkrekic acid production by *B. gladioli* pathovar *cocovenenans*.^303^ Further, pwCF can be colonized by mucor, but the influence of these fungi on morbidity and mortality is currently not well-understood.^304^ It is conceivable that coinfecting mucor fungi could increase bongkrekic acid production by *Burkholderia* spp. in the CF airway. Alternatively, other types of coinfecting fungi could also influence bongkrekic acid production. These possibilities remain to be tested within the context of CF.

#### 2.4.3. Gladiostatin

The genomes of *B. gladioli* BCC0238 and BCC1622 encode a BGC for a *trans*-AT PKS designated as *gds* that is similar to BGCs responsible for production of a class of natural products called glutarimides.^305^ Instead of releasing the polyketide chain with a thioesterase domain, Nakou *et al.* identified an unusual AfsA-like domain at the C-terminus of the last PKS module called a phosphorylated butenolide synthase (PBS) domain. This PBS domain releases the PK chain by condensing a molecule of dihydroxyacetone phosphate to form a phosphorylated butenolide intermediate, which undergoes rearrangements to form gladiostatin (47) (Fig. 26).^305^

**Fig. 26:**
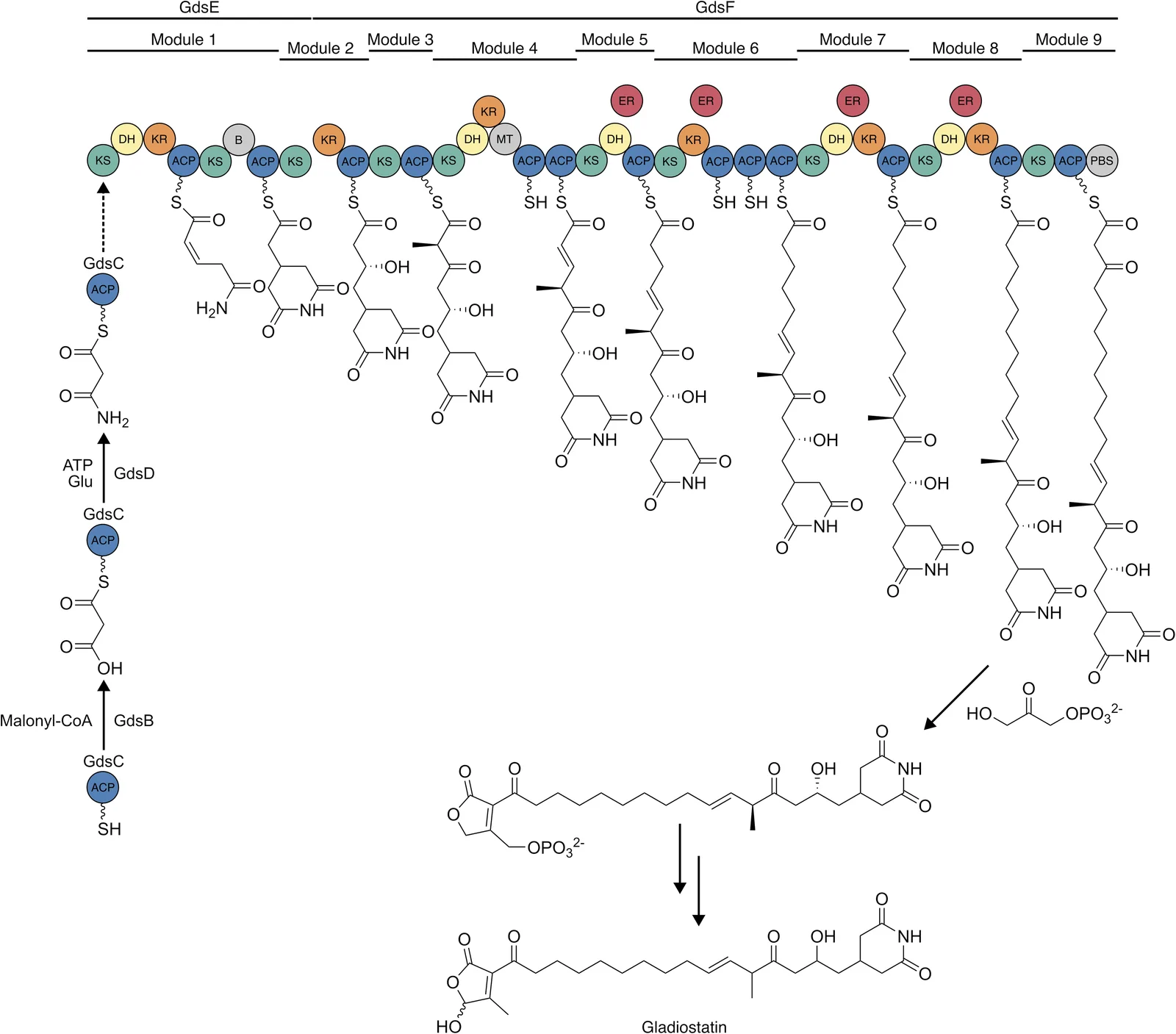
Proposed pathway for gladiostatin biosynthesis. Domain and module organization of the gladiostatin *trans*-AT PKS showing the proposed structure of each ACP-bound intermediate. Proteins and modules are labeled. Domain abbreviations: ACP, acyl carrier protein; B, branching; DH, dehydratase; ER, enoyl reductase; KR, ketoreductase; KS, ketosynthase; MT, methyltransferase; PBS, phosphorylated butanolide synthase. Figured adapted from ref. 306 under the terms of the CC BY 4.0 International license.

Glutarimide natural products are known to possess antifungal and anticancer activity.^307^ Accordingly, gladiostatin is inactive against bacteria and *C. albicans*, but was moderately active against *S. cerevisiae* (MIC: 7.9 µM).^305^ Gladiostatin was active against cancer cell lines derived from colon (IC_50_: 0.82 µM), ovary (IC_50_: 0.24 to 1.4 µM), and pancreas (IC_50_: 0.57 µM) tissues. However, gladiostatin was inactive against a lung cancer cell line,^305^ suggesting some specificity for its anticancer activity. Although the gladiostatin producers were isolated from the CF airway, the role of this natural product in the system remains unknown. It remains possible that gladiostatin is active against other fungi that can colonize the CF airway or exhibits cytotoxicity against the lung epithelium.

#### 2.4.4. Gliotoxin

Gliotoxin (48) is an epipolythiodioxopiperazine natural product produced by *Aspergillus* spp. and other fungi. This compound is produced by a NRPS that condenses l-serine and l-phenylalanine to form a 2,5-diketopiperazine core.^308,309^ A key feature of gliotoxin is the presence of a transannular disulfide bridge installed by the oxidoreductase thioredoxin GliT, which oxidizes a dithiol moiety derived from two molecules of glutathione (Fig. 27).^310^ The oxidized and mature gliotoxin is membrane permeable and converted back into the dithiol form within the reducing environment of a target cell, where it can then engage in redox cycling and exert its cytotoxic effects *via* ROS.^311^ GliT also provides self-resistance to gliotoxin by keeping the toxin in its oxidized form within the producer.^312^ The literature on gliotoxin is extensive. Thus, we will focus on the potential roles of gliotoxin in the CF airway that have been recently uncovered.

**Fig. 27:**
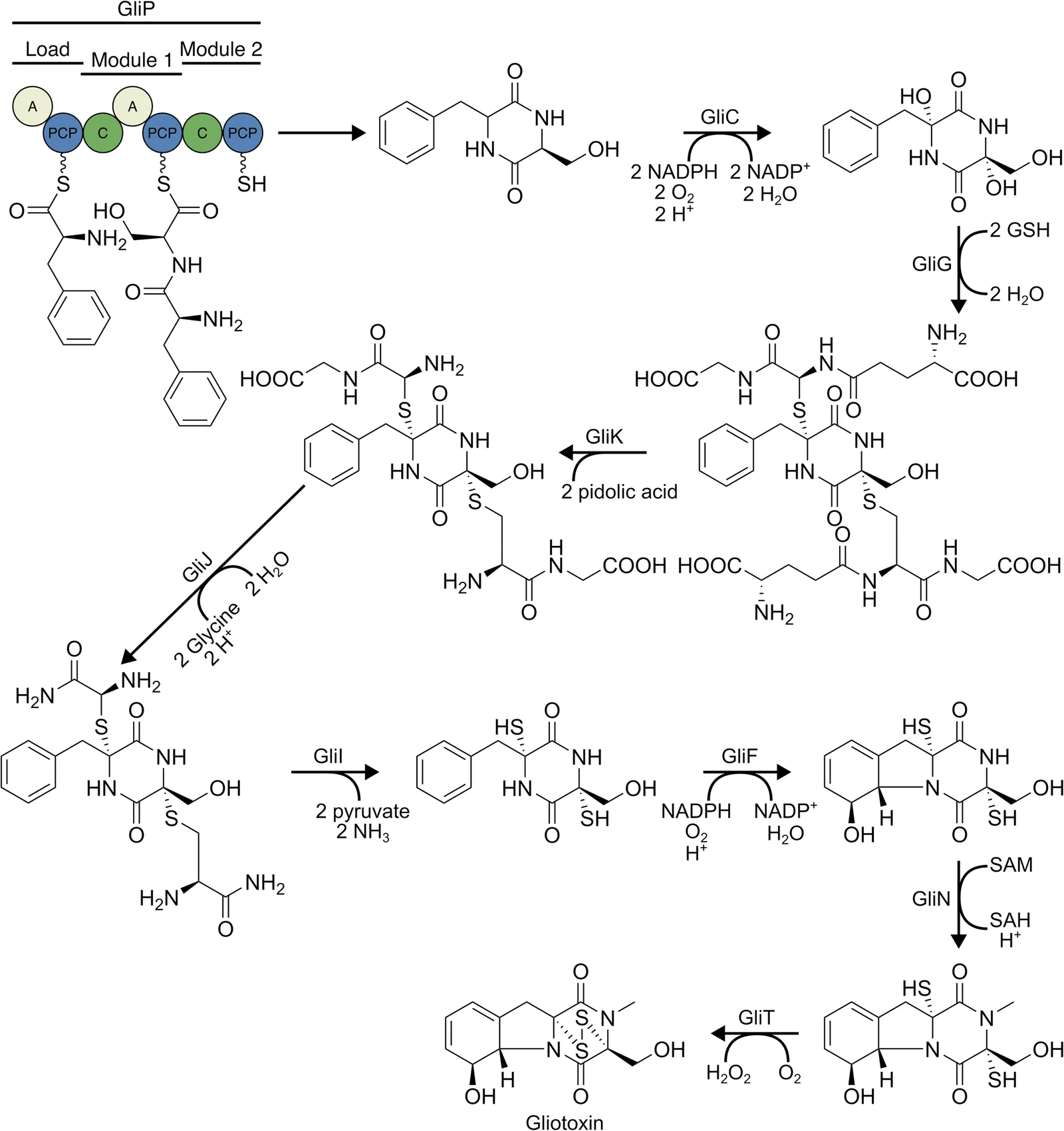
Proposed pathway for gliotoxin biosynthesis. Domain and module organization of the GliP NRPS showing the proposed structure of each PCP-bound intermediate. Protein and modules are labeled. The diketopiperazine core of gliotoxin is formed during chain release from GliP. Domain abbreviations: A, adenylation; C, condensation; PCP, peptidyl-carrier protein.

The genes for gliotoxin biosynthesis are expressed by *A. fumigatus* during murine lung infection and the compound has been detected within the bronchoalveolar lavage (BAL) fluid of pwCF.^313,314^ Gliotoxin disrupts the tight junctions between CF bronchial epithelial cells, which results in loss of barrier integrity, and induces transient chloride ion secretion.^315,316^ Intriguingly, the virulence of *A. fumigatus* may be increased in pwCF, as the presence of a functional CFTR bestows moderate protection against gliotoxin.^315,316^ It has also been suggested that gliotoxin and the inflammatory milieu interact to mediate cytotoxicity. For instance, BAL fluid from pwCF that is *Aspergillus*-culture positive kills human bone-marrow derived mesenchymal stromal cells (hMSCs). Similarly conditioned medium from *A. fumigatus* kills these hMSCs, but medium from a gliotoxin biosynthesis mutant has no effect.^314^

Gliotoxin is increasingly recognized as an important mediator in interactions between *A. fumigatus* and *P. aeruginosa*. During infection, these pathogens can form mixed species biofilms where they compete for limited resources and gliotoxin is important for *A. fumigatus* persistence.^222,317^ For instance, *A. fumigatus* produces gliotoxin during zinc starvation. When gliotoxin enters the reducing environment of the *P. aeruginosa* cytoplasm, it is converted to dithiol gliotoxin, which acts as a high-affinity chelator, and starves the cell for zinc.^318,319^ In response, *P. aeruginosa* produces DnoP (formerly PA4170), which oxidizes the dithiol moiety of epipolythiodioxopiperazines and dithiolopyrrolone natural products and abrogates their activity.^45^ This mechanism arose *via* convergent evolution and DnoP can functionally substitute GliT for gliotoxin biosynthesis and resistance in *A. fumigatus*.^45^ Furthermore, as another potential resistance mechanism, *P. aeruginosa* produces as-of-yet unknown factors that decrease gliotoxin biosynthesis by *A. fumigatus*.^222,320^ Gliotoxin also mediates interspecies interactions between *A. fumigatus* and other members of the CF airway microbiota. This compound inhibits biofilm formation of *B. multivorans*, *S. aureus*, and *S. maltophilia*. However, the specific mechanism underlying these interactions are still unknown.^317^ Together, these studies highlight the importance of gliotoxin as a toxin in the CF airway and as a modulator of the microbiota.

#### 2.4.5. PQS and PQS pathway products

In addition to its role as a signaling molecule, PQS (see 2.3.4 above) activates production of several virulence factors in *P. aeruginosa*, including elastase, lectins, and pyocyanin (see 2.4.6 below).^263,278,321,322^ In a murine infection model a *pqsA* mutant exhibits reduced virulence compared to wild-type strains.^262,323^ Accordingly, production of many of these virulence factors by *P. aeruginosa* is diminished when quinolone production is inhibited, but is restored upon addition of exogenous PQS.^324^ Exposure to purified PQS induces production of ROS in human lung and bronchial epithelial cells, and in macrophages, leading to apoptosis.^325^ Normal human bronchial epithelial cells are sensitive to PQS in concentration as low as 1.9 µM. Further, PQS-induced ROS production impairs production of heme oxygenase-1, an enzyme that is normally induced during oxidative stress to protect cells from hyperoxia.^325,326^ Thus, this cytotoxicity may promote *P. aeruginosa* persistence and chronic infection in the CF airway.^324^ Intriguingly, PQS interferes with quorum sensing in *Burkholderia* spp. and inhibit its virulence (see 2.4.4 above).^284^

DHQ (see 2.3.4 above) is one of the most abundant non-alkyl quinolones produced by the PQS pathway (Fig. 22).^265,322^ DHQ biosynthesis can occur under anaerobic conditions, such as those that can occur in the airways of pwCF during chronic infection. However, under these same conditions, PQS production is reduced due to lack of oxygen.^322,327^ Accordingly, DHQ has been detected directly from *ex vivo* sputum from pwCF with a positive culture history of *P. aeruginosa* infection.^322^ Mutants of *P. aeruginosa* that are unable to produce quinolones exhibit virulence defects in *Caenorhabditis elegans* nematode infection models, and produce less pyocyanin, but mutants that can only produce DHQ and HQNO exhibit similar virulence as the wild type.^322^ PQS, DHQ, and other quinolones produced by *P. aeruginosa* can act as cytotoxic agents and virulence factors, impacting the CF microbiota and directly contributing toward disease.

#### 2.4.6. Pyocyanin

Pyocyanin is a redox-cycling phenazine (see 2.1.10 above) that is formed by the sequential action of the SAM-dependent methyltransferase PhzM, which converts PCA into 5-methyl-PCA, and the flavin-dependent monooxygenase PhzS that catalyzes hydroxylative decarboxylation and redox modification of 5-methyl-PCA to yield pyocyanin (16) (Fig. 7). This redox-active natural product generates ROS and increases the virulence of *P. aeruginosa*.^328,329^ Pyocyanin accepts electrons from NAD(P)H and transfers them to oxygen, generating the superoxide anion (O_2_˙^−^) and H_2_O_2_.^329^ Most human cells including epithelial cells employ various mechanisms to limit ROS exposure, including production of glutathione (GSH). In the presence of ROS, GSH is oxidized by glutathione peroxidase to glutathione disulfide (GSSG), which is reduced back to GSH by glutathione peroxidase. GSH/GSSG redox cycling limits cellular exposure to H_2_O_2_ and protects the cells from cytotoxicity.^329^ However, in pwCF, GSH levels are reportedly decreased, because GSH can be directly and rapidly oxidized by pyocyanin, even in the absence of cell-derived reducing equivalents. GSH directly transfers electrons to pyocyanin and promotes the formation of various types of ROS. Thus, in the presence of pyocyanin, GSH acts both as an antioxidant and as a ROS promoter.^329^ Pyocyanin is detected in sputum (up to 100 µM) and pulmonary secretions (up to 130 µM) from pwCF, where it can alter epithelial cell function and modulate redox-sensitive signaling events. Pyocyanin readily penetrates biological membranes and can inhibit cell respiration and cell growth. At a high concentration (100 µM), pyocyanin acts as a proinflammatory agent by disrupting the bronchial epithelial cells and impairing ciliary function.^330–332^ An imbalance in protease–antiprotease activity is detected in the airways of pwCF, and pyocyanin contributes to this imbalance by inactivating the α_1_-protease inhibitor, which is known to modulate serine protease activity in lung.^22,333^ Pyocyanin further increases IL-8 secretion and suppresses Regulated upon Activation, Normal T cell Expressed, and Secreted (RANTES) by inhibiting tumor necrosis factor alpha (TNF-α) in human airway epithelial cells.^334^ In addition, pyocyanin inhibits catalase activity, which allows oxidative damage to accumulate in the airway epithelium.^22,335^

Pyocyanin is required by *P. aeruginosa* for its growth and persistence during infection, as pyocyanin biosynthesis mutants exhibit fitness defects and are cleared readily compared to wild-type strains.^22,336^ Pyocyanin contributes to the rapid killing of *C. elegans* in an infection model and the infection of *P. aeruginosa* in different hosts can depend on the production of the molecule.^22^ Pyocyanin aids in *P. aeruginosa* iron acquisition by reducing ferric iron to ferrous iron and releasing it from transferrin.^337^ Key components of the citric acid cycle like aconitase are negatively affected by pyocyanin, thereby inhibiting ATP production.^328,338^ In addition, pyocyanin inhibits human V-ATPase activity in lung epithelial cells.^339,340^ V-ATPase is known to maintain the calcium level required for proper functioning of ciliary motility, which is disrupted by pyocyanin.^339^ Thus, pyocyanin is a multifunctional virulence factor that enables *P. aeruginosa* to modulate host defense by promoting oxidative stress and enhancing its persistence.

#### 2.4.7. Pyoverdine

In addition to acting as a siderophore (see 2.2.9 above), pyoverdine is a virulence factor and is directly cytotoxic toward eukaryotic cells. Pyoverdine can kill macrophages, contribute directly to the mortality of *C. elegans* in infection models, and increases virulence by *P. aeruginosa* in murine burn wound, endocarditis, and pneumonia models.^150,184,341–343^ These effects may result from pyoverdine directly damaging host mitochondria.^344^ Pyoverdine promotes its own production using an autoregulatory circuit and production of two additional virulence factors, exotoxin A (ToxA) and the serine protease PrpL.^345^ Pyoverdine further regulates rhamnolipid production (see 2.4.8 below).^346^ This regulation occurs through a signaling cascade where ferripyoverdine interacts with a membrane-associated receptor complex consisting of FpvA–FpvR–TonB1 to activate the alternative sigma factor PvdS, which in turn promotes expression of pyoverdine biosynthesis, *toxA*, and *prpL*.^345,347,348^ The concentration of pyoverdine in sputa from pwCF ranges 0.48–1.55 µM, which exceeds the threshold concentration of 0.6 µM to stimulate the FpvA–FpvR–TonB1 complex.^345,349^ Thus, *in vivo* these virulence factors are produced contributing to the virulence of the *P. aeruginosa*. Accordingly, the virulence of *P. aeruginosa* decreases when pyoverdine biosynthesis is inhibited.^150,184,350^ Pyoverdine biosynthesis mutants of *P. aeruginosa* grew poorly in the lungs of immunosuppressed mice, even though they could still produce the alternative siderophore pyochelin (see 2.2.8 above), suggesting that the former is more critical for establishing lung infections. Together, the virulence factors activated by pyoverdine enhances the virulence of *P. aeruginosa* and are critical for colonization and interactions with other microbes, highlighting the potential role that these siderophores may play beyond iron acquisition.

#### 2.4.8. Rhamnolipids

Rhamnolipids are rhamnose-containing glycolipid biosurfactants produced by *P. aeruginosa*, which are formed at the convergence of two precursor pathways.^331,351^ On one branch, glucose-6-phosphate is converted to glucose-1-phosphate by the phosphoglucomutase AlgC.^352^ Next, RmlABCD converts glucose-1-phosphate into dTDP-l-rhamnose, which serves as the rhamnosyl donor.^352,353^ On the other branch, RhlA catalyzes the formation of 3-(3-hydroxyalkanoyloxy)alkanoic acids (HAAs) that serve as rhamnosyl acceptors using β-hydroxy fatty acids derived from primary metabolism.^351,354^ Subsequently, the HAAs are glycosylated by RhlB using dTDP-l-rhamnose to yield rhamnolipid I (l-rhamnosyl-3-hydroxydecanoyl-β-hydroxydecanoate) (49),^355^ which is then glycosylated by RhlC using dTDP-l-rhamnose to yield rhamnolipid II (di-rhamnolipid; l-rhamnosyl-l-rhamnosyl-3-hydroxydecanoyl-β-hydroxydecanoate) (50) (Fig. 28).^351,356^

**Fig. 28:**
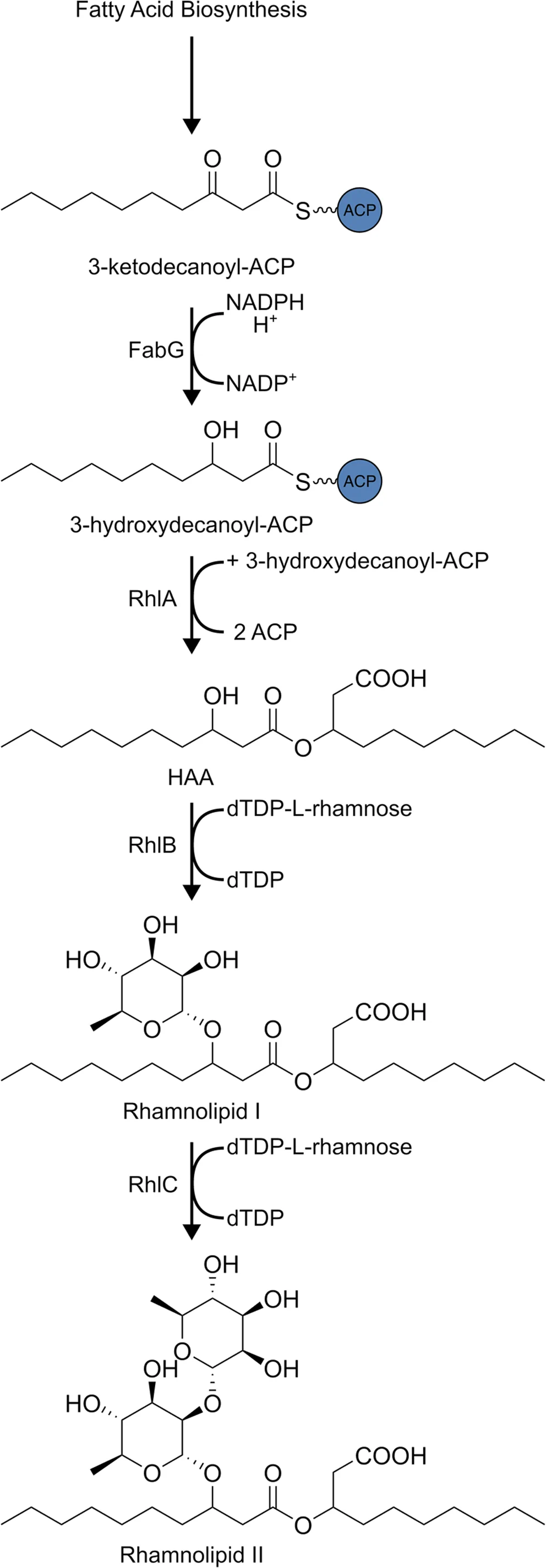
Pathway for rhamnolipid biosynthesis.

The rhamnolipids contribute to the virulence of *P. aeruginosa* through multiple mechanisms that are relevant to the airways of pwCF.^357,358^ These compounds can alter transepithelial resistance and disrupt tight junctions that normally prevent pathogens from accessing the basolateral domain of host epithelial cells thus aid in *P. aeruginosa* infiltration of the epithelium.^357,359,360^ Rhamnolipid micelles can also cause membrane damage in human lung epithelial cells.^358^ In addition, rhamnolipids cause ciliostasis, stimulate mucus secretion, and inhibit epithelial ion transport.^331,361^ In the airways of pwCF, where mucociliary clearance is already impaired, these effects may compound to worse disease. The concentration of rhamnolipids in the sputum of pwCF reaches up to 16 µM, which is sufficient to reduce mucociliary transport *in vivo* and ciliary beat frequency of the human respiratory epithelium *in vitro*.^331,362^ Further, rhamnolipids causes structural changes in human monocyte-derived macrophages, enabling *P. aeruginosa* to resist phagocytosis and pulmonary clearance.^363,364^ Rhamnolipids further also induce rapid necrosis of polymorphonuclear leukocytes *in vitro* and *in vivo*, compromising immune defense in the CF airways.

Rhamnolipids also mediate interspecies interactions between members of the CF airway microbiota. For instance, these compounds are critical for maintaining the biofilm architecture of *P. aeruginosa* and can help block colonization by other pathogens. These compounds can disrupt the biofilm of the fungi *C. albicans*.^365^ A mixture of rhamnolipid I and II exhibited activity against several bacteria and fungi, including *K. pneumoniae* (MIC: 0.78 to 1.0 µM) and *S. aureus* (MIC: 49.2 to 63.4 µM). Further, in coculture with *Streptococcus constellatus*, rhamnolipid I directly reduces established biofilms, and autoinducer 2 produced by *S. constellatus* can activate rhamnolipid biosynthetic genes in *P. aeruginosa*.^366^ Rhamnolipid levels are also increased in mixed *A. fumigatus*–*P. aeruginosa* biofilms cultured under hypoxic conditions, reflective of the CF airway.^222^ This increased rhamnolipid production may serve to inhibit the growth of *A. fumigatus* by disrupting β-1,3-glucan synthase.^367^ Together, these activities suggest that rhamnolipids contribute to the persistence of *P. aeruginosa* in the CF airway by simultaneously impairing mucociliary clearance, suppressing the innate immune response, and inhibiting competitors.

#### 2.4.9. Staphyloxanthin

Staphyloxanthin (51) is a glucosyl diester of diaponeurosporenoic acid and pentadecanoic acid.^368^ This carotenoid is responsible for the characteristic yellow pigment of *S. aureus* that is the namesake of this organism. Though pigment production is not universal, the *crt* BGC for staphyloxanthin are widely conserved across *Staphylococcus* spp.^369^ The biosynthesis of staphyloxanthin proceeds from head-to-head condensation of two molecules of farnesyl diphosphate by CrtM to form dehydrosqualene.^368,370^ Next, CrtN performs successive reduction to form 4,4′-diaponeurosporene and CrtP oxidizes a terminal methyl group to form 4,4′-diaponeurosporenoic acid. Finally, CrtQ and CrtO attach glucose and pentadecanoic acid, respectively, to form staphyloxanthin (Fig. 29).^368,371^ The fatty acid tethers staphyloxanthin to the cytoplasmic membrane, where this compound regulates membrane fluidity and susceptibility to antimicrobial peptides.^372,373^

**Fig. 29:**
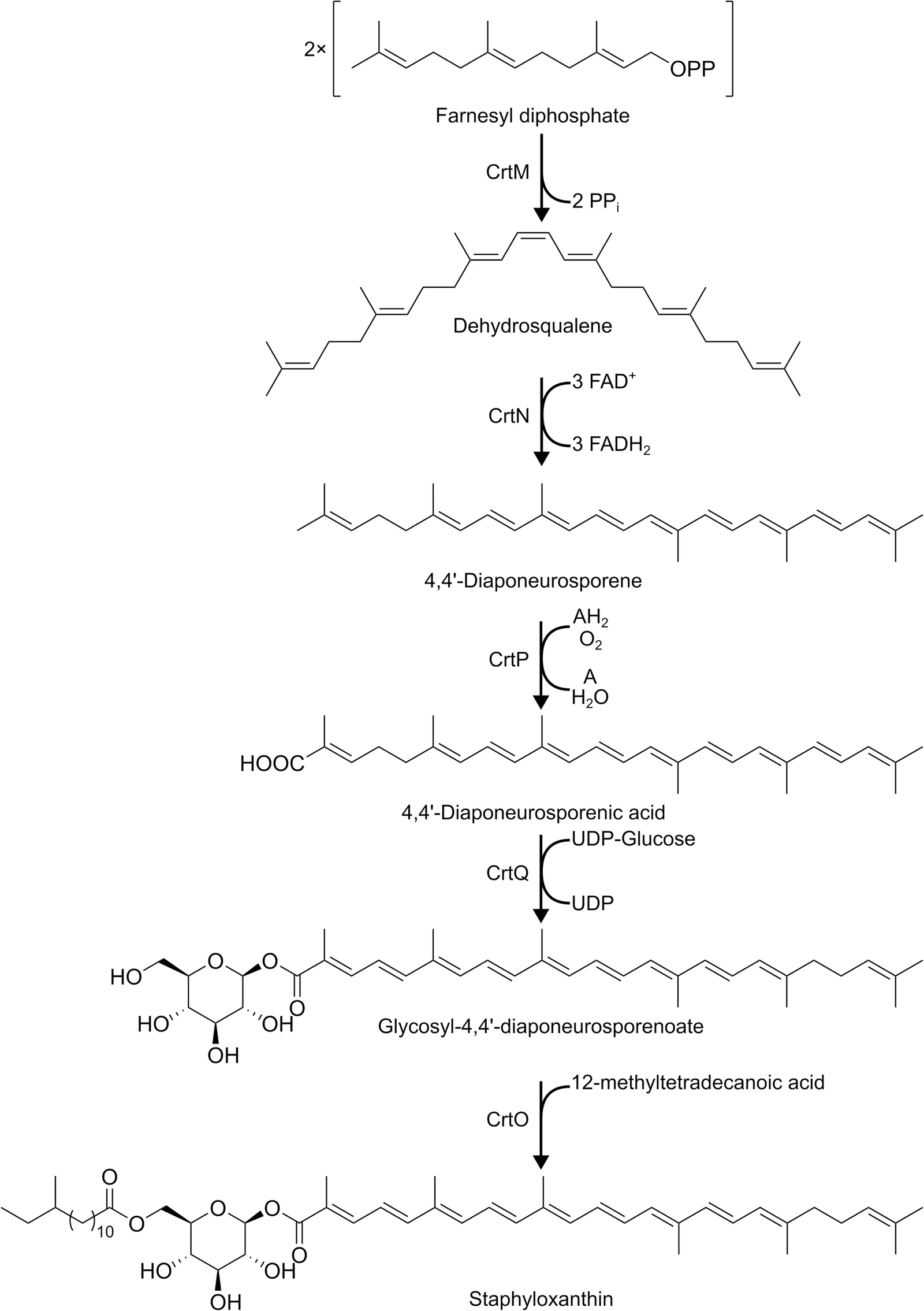
Proposed pathway for staphyloxanthin biosynthesis. The identity of the electron donor (AH_2_) used by CrtP has not been specifically identified. It is also not currently known how 12-methyltetradecanoic acid is activated for incorporation into the final product. Abbreviations: OPP, diphosphate.

The conjugated bonds of staphyloxanthin act as an antioxidant and protects *S. aureus* from reactive oxygen and chlorine species, including those produced by myeloperoxidase within neutrophils.^374–376^ Though in the latter case, it is suspected that the biosynthetic intermediate 4,4′-diaponeurosporenoic acid is responsible primarily for protection against hypochlorous acid.^376^ Regardless, total carotenoid levels are correlated with *S. aureus* survival in response to ROS and staphyloxanthin biosynthesis mutants are more susceptible to killing by H_2_O_2_ and neutrophils.^376–378^ Indeed, *S. aureus* isolates that produce lower levels of staphyloxanthin also exhibit a more pronounced stress response when treated with H_2_O_2_.^377^ There is currently no direct evidence that staphyloxanthin contributes to *S. aureus* virulence in CF. However, neutrophils are the most abundant immune cell found within the CF airway.^379^ Outside of this context, staphyloxanthin production has been correlated with the virulence of *S. aureus* in non-healing diabetic foot ulcers and other skin wounds, but in other contexts there is no correlation between pigmentation and virulence.^377,380,381^ The production of staphyloxanthin is under control of the sigma B regulon and mutations in these genes can abrogate its production.^377,382^ Notably, *S. aureus* isolates from chronic infections frequently possess mutations in the regulator gene *rbsU*,^383^ which may suggest that staphyloxanthin is more important during early infection in the CF airway when inflammation is high.

Compounds that reduce staphyloxanthin production by inhibiting CrtM or CrtN have been proposed as potential antivirulence drugs, but many of these compounds are cytotoxic at their inhibitory concentrations.^384–387^ However, Yu and Zhang *et al.* recently discovered that the FDA-approved antihistamine clemizole directly inhibits staphyloxanthin production with an IC_50_ value of 103 nM, and is not cytotoxic or bactericidal at concentrations up to 10 µM.^381^ These authors also showed that clemizole is as effective as vancomycin at treating murine burn wounds infected with *S. aureus*.^381^

In addition to acting as a virulence factor, staphyloxanthin is also involved in interspecies interactions with *P. aeruginosa*. HQNO (see 2.1.6 above) stimulates staphyloxanthin production by *S. aureus*.^378^ This pigment protects *P. aeruginosa* from H_2_O_2_ and neutrophil-mediated killing.^388^ As mentioned above, these bacteria frequently cooccur within the CF airway, suggesting that *S. aureus* has the potential to protect *P. aeruginosa* during chronic infection, in addition to providing them with a source of lactate (see 2.1.6 above). Of note, clemizole does inhibit staphyloxanthin production stimulated by *P. aeruginosa*, and could potentially be used to treat infections in people with CF.^381,388^

#### 2.4.10. Toxoflavin

Toxoflavin (also called xanthothricin) (52) is an azapteridine-containing natural product produced by multiple species of *Burkholderia*. The biosynthesis of toxoflavin largely mirrors riboflavin biosynthesis and begins with ToxB catalyzing the conversion of guanosine triphosphate (GTP) into 2,5-diamino-6-ribosylamino-4(3*H*)-pyrimidinone 5′-phosphate. ToxE then catalyzes deamination of this intermediate to form 5-amino-6-ribosylamino-2,4(1*H*,3*H*)-pyrimidinedione 5′-phosphate. After dephosphorylation, ToxC is proposed to use glycine or a derivative to generate the triazine ring. ToxD subsequently catalyzes oxidative cleavage of the ribityl sidechain to generate the azapteridine intermediate 1,6-didemethyltoxoflavin. Finally, ToxA catalyzes stepwise *N*-methylation to yield toxoflavin (Fig. 30).^389,390^ This compound is traditionally considered to be a phytotoxin produced by plant pathogenic serovars of *B. gladioli* and *B. glumae*.^391^ However, the genomes of many *B. gladioli* strains isolated from the airways of pwCF encode the *tox* BGC, and toxoflavin production has been observed in synthetic cystic fibrosis sputum medium, suggesting that the compound may also be produced *in vivo*.^301^ Toxoflavin was first identified as an antibiotic,^392^ but its effectiveness differs across studies, and it tends to exhibit weak antibacterial activity (reported MICs are typically ≥10 µM).^393–395^ However, exposure to toxoflavin does cause significant changes in the transcriptional response of *E. coli*, particularly affecting oxidative phosphorylation.^395^ This transcriptional reprogramming is consistent with the redox cycling activity of toxoflavin. Cells treated with toxoflavin increase oxygen uptake and produce H_2_O_2_ and other ROS and the inhibitory effects can be rescued with antioxidants.^393,396,397^

**Fig. 30:**
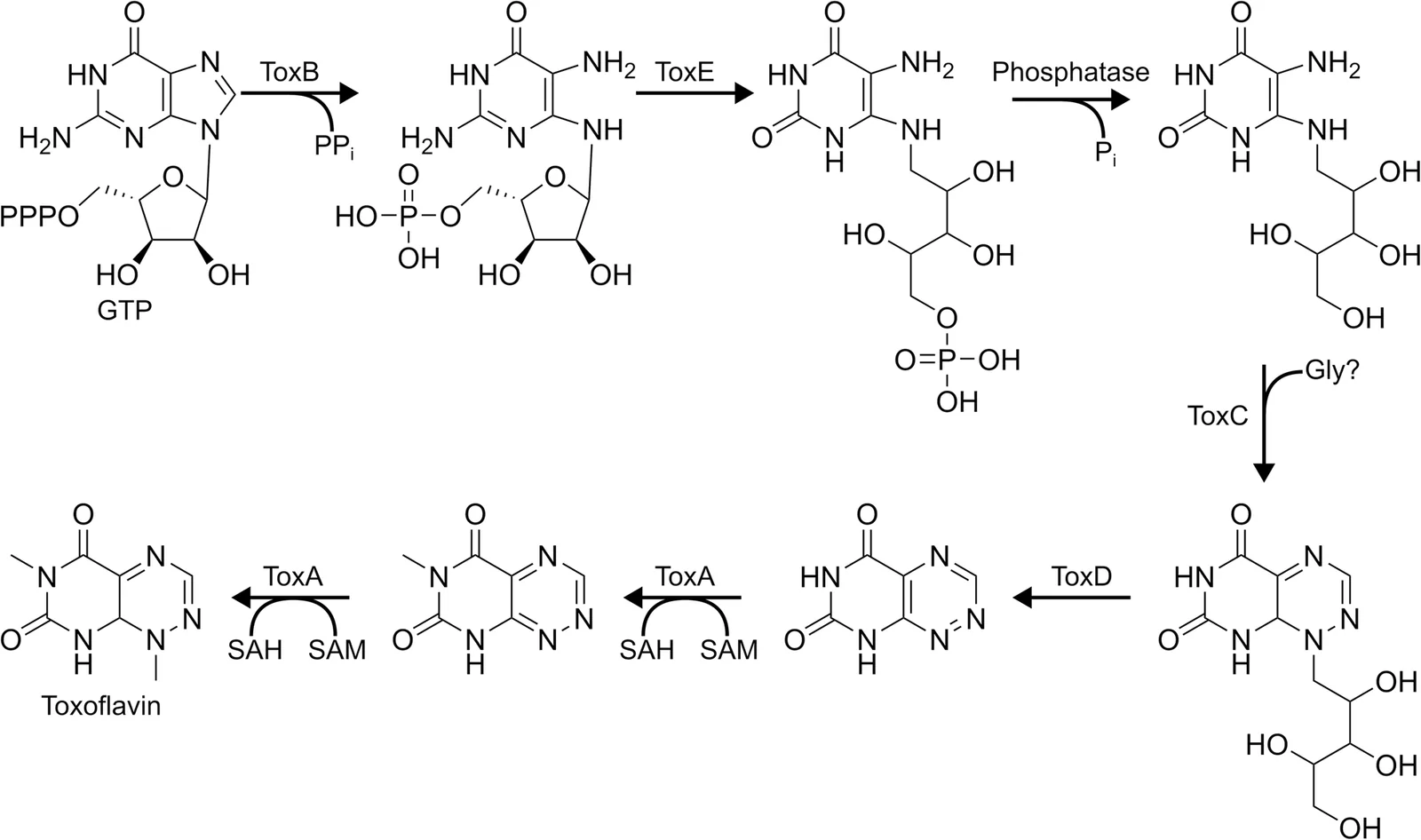
Proposed pathway for toxoflavin biosynthesis. The identity of the phosphatase is not currently known. Glycine is proposed to be involved in generation of the triazine ring. Abbreviations: OPPP, triphosphate.

Toxoflavin inhibits a range of cellular targets in eukaryotic cells, including Tcf-β-catenin complexes,^398^ the class III histone deacetylases SIRT1/2,^399^ selenoprotein thioredoxin reductase,^396^ inositol-requiring enzyme 1α,^400^ and separase,^401^ and it has been suggested that this compound could possess anticancer activity. Accordingly, toxoflavin and analogues have been shown to inhibit different human cancer cell lines, including lung-derived lines, with low micromolar IC_50_ values and are active against xenograft tumor models.^397,399,401^ These compounds cause dsDNA breaks, cell cycle arrest, and apoptosis.^397,400,401^ In each of these cases, the activity of toxoflavin was mediated by ROS production. However, given its strong redox cycling, toxoflavin has been proposed as an assay interference compound in library screens.^402^ Nevertheless, molecular docking suggests that toxoflavin and its analogues D43 and walrycin B bind to their respective targets.

In addition to its potential anticancer activity, toxoflavin contributes to interspecies bacterial interactions, which may be relevant to the CF airway. For instance, coculture of the *B. cepacia* complex with toxoflavin-producing *B. gladioli* increases survival of the former when treated with several antibiotics, including ciprofloxacin,^403^ which is commonly prescribed for pulmonary exacerbations.^403^ In contrast, toxoflavin may also exert some bactericidal activity (see above). Similarly to staphyloxanthin (see 2.4.9 above) production, strains of the phytopathogen *Pantoea ananatis* that produce carotenoids are more resistant to toxoflavin, presumably because the carotenoids act as antioxidants to detoxify H_2_O_2_.^404^ Although the precise role toxoflavin plays in the CF airway is not well-understood, the preponderance of evidence suggests that this compound has a potential role in the virulence or ecology of *Burkholderia*.

## 3. Conclusions and outlook

The clinical and ecological significance of natural products in many human diseases remain poorly understood. However, there is a rich history underlying the natural products produced by bacterial and fungal pathogens of the CF airway. These compounds exhibit a wide range of biological activities, ranging from antibiotics and signals that influence interspecies interactions, siderophores that facilitate nutrient acquisition from the host, and virulence factors that contribute to pathogenesis. This wealth of information makes the CF airway microbiome a well-suited system to uncover fundamental mechanisms governing how natural products impact microbiome structure and function that will extend broadly across hosts and diseases.

The pathogens highlighted throughout this article are the best characterized inhabitants of the CF airway. However, advances in culture-independent approaches have revealed the CF airway is colonized by a microbiota that includes other commensal bacteria and opportunistic pathogens found within the upper respiratory tract and oral cavity.^63,405^ These microbes can enter the lower airways, where they establish long-term communities in the airways and lungs of pwCF.^406^ The composition of the CF airway microbiota has been linked to differential disease outcomes, and recent microbiome-wide association studies suggest that bacterial interactions are strong predictors of declining lung function in CF.^407^ To colonize the CF airway, microbes must attach to the epithelium, accommodate or evade the host immune system, cope with decreased access to freely available nutrients, and interact with other resident microbes. As exemplified above, microbes produce bioactive natural products to mediate interspecies interactions (Fig. 31).^408^ We previously determined that BGCs are present within bacterial genera that are capable of colonizing the airways of pwCF (Fig. 32).^13^ Given the wealth of compounds already described from CF pathogens, we anticipate that there is a rich future using the CF airway microbiota as a novel source for natural product discovery. These compounds could represent the next antibiotics or targets for the development of anti-virulence strategies.

**Fig. 31:**
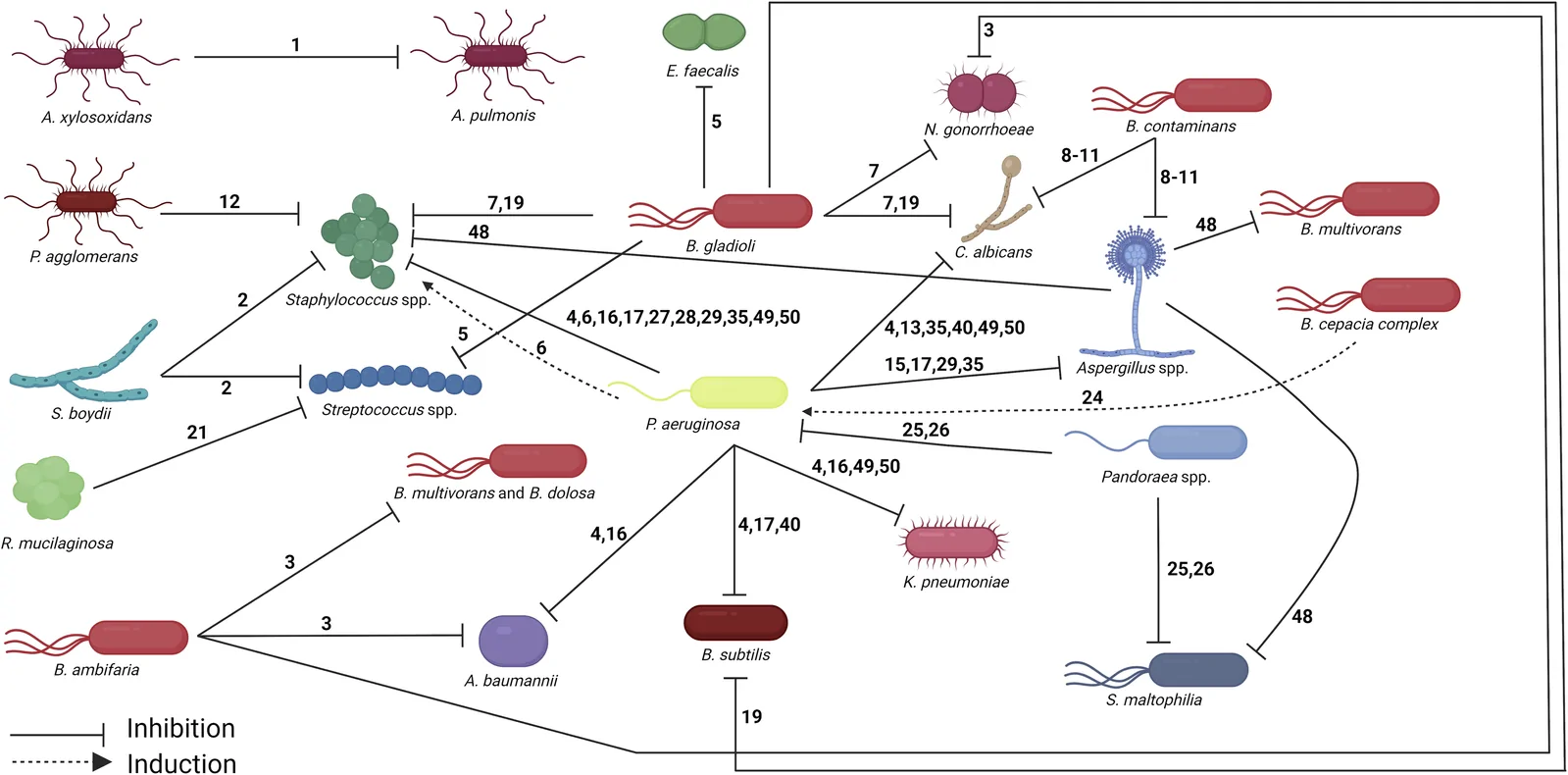
Interspecies interactions are widespread among the CF airway microbiota. Solid and dashed arrows indicate inhibition and activation of natural product production, respectively. The numbers above each line correspond to natural products described in text: 1, achromonodin-1; 2, boydone A; 3, enacyloxin IIa; 4, fluopsin C; 5, icosalide A1; 6, HQNO; 7, gladiolin; 8–11, occidiofungin; 12, pantocin A; 13, PCA; 15, 1-hydroxyphenazine; 16, pyocyanin; 17, pyrrolnitrin; 19, bolagladin B; 20, cepabactin; 24, ornibactin; 25, pandorabactin A; 26, pandorabactin B; 27, 28, pyochelin; 29, pyoverdine; 35, 3-oxo-C12-HSL; 40, PQS; 48, gliotoxin; 49, rhamnolipid I; 50, rhamnolipid II. Figure was generated using BioRender.

**Fig. 32:**
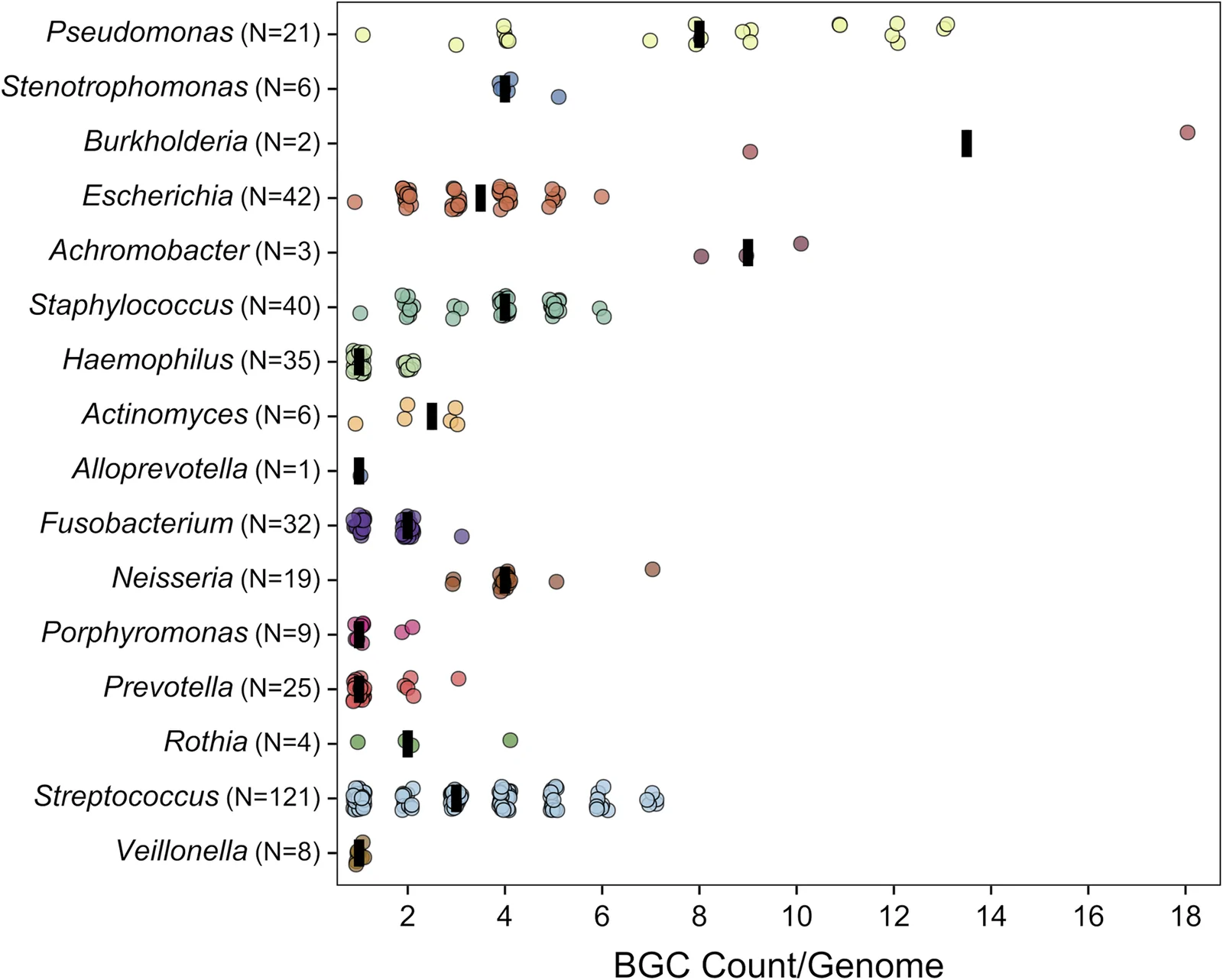
The CF airway microbiota possesses biosynthetic potential for natural product production. Plot showing the number of BGCs per genome for aerodigestive tract bacteria genera that can also colonize the airways of pwCF. Each point represents a genome from a single strain of the corresponding genus. The vertical black bars represent the median BGC count per genome. This analysis only includes genomes where no fragmented BGCs were detected. Data for this figure was from ref. 13 under the terms of the CC BY 4.0 International license.

## 4. Conflicts of interest

There are no conflicts of interest to declare.

## Data Availability

No primary research results, software or code have been included and no new data were generated or analysed as part of this review.
